# CNV analysis in Chinese children of mental retardation highlights a sex differentiation in parental contribution to *de novo* and inherited mutational burdens

**DOI:** 10.1038/srep25954

**Published:** 2016-06-03

**Authors:** Binbin Wang, Taoyun Ji, Xueya Zhou, Jing Wang, Xi Wang, Jingmin Wang, Dingliang Zhu, Xuejun Zhang, Pak Chung Sham, Xuegong Zhang, Xu Ma, Yuwu Jiang

**Affiliations:** 1 Department of Pediatrics, Peking University First Hospital, Beijing, China; 2 National Research Institute of Family Planning, Beijing, China; 3 MOE Key Laboratory of Bioinformatics, Bioinformatics Division and Center for Synthetic and Systems Biology, TNLIST/Department of Automation, Tsinghua University, Beijing, China; 4 Department of Psychiatry and Centre for Genomic Sciences, Li Ka Shing Faculty of Medicine, The University of Hong Kong, Hong Kong SAR, China; 5 Department of Medical Genetics, The Capital Medical University, Beijing, China; 6 Shanghai Institute of Hypertension, Shanghai, China; 7 Institute of Dermatology and Department of Dermatology at No.1 Hospital, Anhui Medical University, Heifei, Anhui, China

## Abstract

Rare copy number variations (CNVs) are a known genetic etiology in neurodevelopmental disorders (NDD). Comprehensive CNV analysis was performed in 287 Chinese children with mental retardation and/or development delay (MR/DD) and their unaffected parents. When compared with 5,866 ancestry-matched controls, 11~12% more MR/DD children carried rare and large CNVs. The increased CNV burden in MR/DD was predominantly due to *de novo* CNVs, the majority of which (62%) arose in the paternal germline. We observed a 2~3 fold increase of large CNV burden in the mothers of affected children. By implementing an evidence-based review approach, pathogenic structural variants were identified in 14.3% patients and 2.4% parents, respectively. Pathogenic CNVs in parents were all carried by mothers. The maternal transmission bias of deleterious CNVs was further replicated in a published dataset. Our study confirms the pathogenic role of rare CNVs in MR/DD, and provides additional evidence to evaluate the dosage sensitivity of some candidate genes. It also supports a population model of MR/DD that spontaneous mutations in males’ germline are major contributor to the *de novo* mutational burden in offspring, with higher penetrance in male than female; unaffected carriers of causative mutations, mostly females, then contribute to the inherited mutational burden.

Rare copy number variations (CNVs) are well established as a risk factor in a range of neurodevelopmental disorders (NDD)^1^,^2^,^3^,^4^. Chromosome microarray (CMA) has been recommended as the first-tier diagnostic test for newborns with developmental delay and congenital anomalies^5^. Technically, it has evolved from detection of submicroscopic chromosome changes^6^, to recent high-resolution analysis of single gene disruptions^7^,^8^,^9^. The clinical utilities of CMA results on patient management have also been demonstrated^10^,^11^.

Despite its widespread use in molecular diagnosis, challenges remain for implementing the CMA workflow. One is technological: how to maximize the sensitivity for variants discovery? Compared with array comparative genomic hybridization (CGH), single nucleotide polymorphism (SNP) arrays may not be optimized for signal-to-noise ratio reflecting copy number changes^12^,^13^. But the disadvantage could be offset by increased probe densities and exploiting the allelic specific information^14^. Nevertheless, accurate discovery of small CNVs has been demonstrated difficult for most platforms^15^. The improvements in both analytical methods and technology will be needed.

The second issue is interpretational: how to distinguish pathogenic from benign CNVs^16^,^17^? The inheritance status may not be used as a single criterion for evaluation: *de novo* occurrence may not necessarily imply pathogenicity^18^; and the role of inherited CNVs should not be underestimated^19^, but their interpretations are complicated by the incomplete penetrance and/or variable expressivity. For clinical interpretation, an evidence-based review approach has been recommended^20^. For this task, it is crucial to incorporate the findings from latest research.

Most of the published CNV studies in NDD were conducted in European populations. Studies in additional population can be valuable. They allow an assessment of potential population difference in global CNV burden and locus-specific associations, which are still not fully clear. As many rare variants including CNVs are population specific, findings from non-European populations may also help prioritize candidate genes by discovering new mutations^21^.

In the present study, we performed a comprehensive analysis of SNP array data of 287 Chinese children of mental retardation and/or developmental delays (MR/DD) and their unaffected parents. To shed light on the pathogenic roles of *de novo* and inherited CNVs, the CNV burdens in both children and their unaffected parents were compared against ~6,000 ancestry-matched controls. Pathogenic CNVs were identified by carefully reviewing evidence from databases and literature. We compared the findings with previous reports in European populations; highlighted gender differences; and documented several cases of pathogenic and benign CNVs that may help refine the genome-wide dosage sensitivity map.

## Results

We analyzed CNVs in 287 children (189 males, 98 females) referred to our department for DNA testing with a general diagnosis of MR/DD. Their phenotypes encompassed a wide range of NDD including congenital malformation, craniofacial dysmorphology, cardiac/neurological defects, epilepsy/seizure, autism, hyperactivity, etc. All of them showed normal karyotype and were negative for the test of subtelomeric aberrations. Whenever possible, the DNA samples from their parents were also genotyped; and a total of 510 of them passed quality control (QC) for CNV analysis (Supplementary Table S1). CNVs identified in this clinical cohort were compared to 5,866 adult controls not ascertained for NDD.

**Figure 1:**
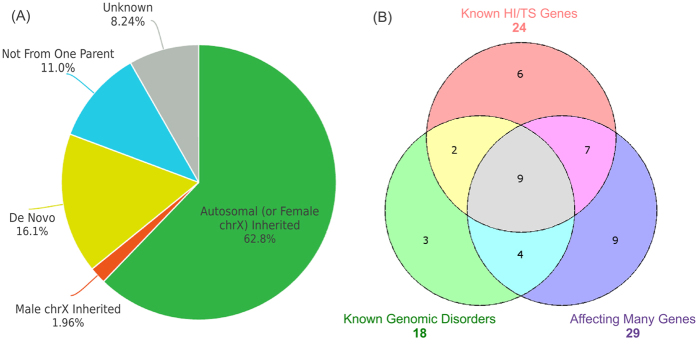
The yield of ultra-rare CNVs from SNP array analysis. (**A**) A total of 255 ultra-rare CNVs were identified in 287 patients with MR/DD; the proportions of different inheritance status are shown in a pie chart. “Not from one parent”: in parent-offspring pairs, the child’s CNV was not found in the available parent; “unknown”: undetermined inheritance as neither parent was available for testing. (**B**) Pathogenicity of CNVs was evaluated based on three criteria: associated with known genomic disorders, deleting haplo-insufficient (HI) or duplicate triplo-sensitive (TS) genes, and affecting large number of genes (details given Materials and Methods). A total of forty CNVs in patients were evaluated as pathogenic. The number of CNVs fulfilling those overlapping criteria is displayed as a Venn diagram.

We mainly focused on ultra-rare CNVs with population frequency <0.1%. A total of 255 CNVs (124 gains and 131 losses) were identified in 173 (60.2%) affected children, and 368 CNVs (177 gains and 191 losses) in 300 (58.4%) parents. 203 CNVs (88 gains and 115 losses) identified in parents were not found in patients. The proportions of male and female carriers of ultra-rare CNVs were similar to the gender composition (P > 0.2, chi-square test) (Supplementary Table S2). The sizes of CNV in patients ranged from <10 kb to >30 Mb (median 137 kb, inter-quartile range (IRQ): 62–380 kb), significantly larger than CNVs found in parents only (median 84 kb, IRQ: 40–167 kb; P = 2.2E-05, Mann-Whitney). The full list of annotated ultra-rare CNVs was given in Supplementary Table S8 and S9. The inheritance for more than 80% ultra-rare CNVs in patients could be determined (Fig. 1A, Supplementary Table S4). We identified and validated 41 *de novo* CNVs in 39 patients, including 2 patients each carrying 2 *de novo* CNVs.

### CNV Burdens

We first compared the burden of ultra-rare and large CNVs between MR/DD patients and controls (Table 1). All comparisons were restricted to CNVs in autosomes. Overall, 18.5% of the patients carried CNVs >= 500 kb compared to 6.4% in controls (OR: 3.32, P = 1.69E-11, Fisher’s Exact Test). The increased CNV burden was mainly due to large deletions, which were carried by 10.1% patients compared to 1.1% controls (OR: 10.18, P = 4.38E-17); whereas the burden of duplication was only modest (8.4% vs. 5.3%, OR: 1.64, P = 0.032). The CNV burden was slightly higher in female (20.4%) than male patients (17.5%), but not statistically different (OR: 1.21, P > 0.5). Likewise, CNVs overlapping the coding sequences (CDS) of at least 8 genes were also significantly enriched in patients (12.5%) compared to controls (1.7%; OR = 8.52, P = 1.04E-18), with a higher enrichment of deletions (OR = 19.7) than duplications (OR = 3.61), and similar between female (13.3%) and male patients (12.2%). The 10~12% increase in CNV burden of patients compared to controls was consistent across different CNV size thresholds (Supplementary Table S6).

As some previous studies^19^,^22^,^23^ defined rare CNVs by frequency of <1%, we also tested the burden of low frequency but not ultra-rare (0.1~1% population frequency) CNVs (Supplementary Table S7). There was a deficit of CNVs in this frequency range as compared with ultra-rare CNVs, because most rare variants including CNVs are private. The proportion of individuals carrying CNV >500 kb was similar between patients (3.1%) and controls (3.5%). Only one such CNV affecting at least 8 genes was found in patients (0.3%), not statistically deviating from the control rate of 0.7% (P > 0.5). The lack of enrichment of low frequency CNVs suggests that most high-penetrant large pathogenic CNVs should be found in the ultra-rare CNVs.

**Figure 2:**
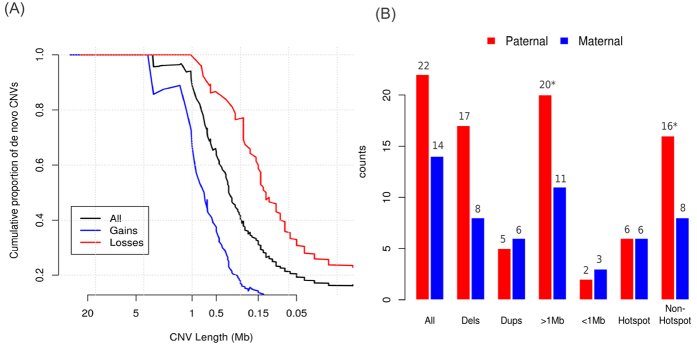
Properties of *de novo* CNVs. (**A**) Cumulative proportion of *de novo* CNVs as a function of physical sizes. More than 90% of all ultra-rare CNVs greater than 1 Mb occur *de novo*; and large deletions are more likely *de novo* as compared with duplications of similar sizes. (**B**) Parental-of-origin for different classes of *de novo* CNVs. The majority of *de novo* CNVs arise on the paternal allele. The observed paternal bias was mainly driven by the CNVs larger than 1 Mb and/or outside known hotspots for non-allelic recombination (*p < 0.1, by exact binomial test against equal chance).

If a portion of large pathogenic CNVs found in patients were inherited, then we would expect to find an increased burden in their parents. We then analyzed the CNV burden in the parents of MR/DD patients. Overall, 7.1% of the parents carried CNVs >500 kb and 2.4% carried CNVs affecting at least 8 genes, both of which were slightly higher but not statistically different from controls (6.4% with CNV >500 kb, 1.7% with CNVs >= 8 genes). The result suggests that the increased CNV burden observed in MR/DD patients are predominantly due to *de novo* ones. Indeed, most large CNVs found in patients occurred *de novo* (Fig. 2A; Supplementary Figure S6). E.g., Over 90% of CNVs larger than 1 Mb or affecting at least 10 genes were *de novo*. And larger deletions were more likely occurred *de novo* than duplications of similar sizes or similar number of affected genes; suggesting that large deletions are less tolerated. Using parental genotypes and allelic-specific signals, we inferred the parental of origin for 36 *de novo* CNVs. Consistent with previous findings^24^,^25^, the majority (n = 22, 61.1%) of them arose on the paternally derived alleles. The paternal bias was mostly due to large deletions and/or CNVs outside rearrangement hotspots (Fig. 2B).

Although the parents as a whole did not have significantly increased CNV burden, when stratified by gender, we observed 4.3% of the mothers carried CNVs affecting at least 8 genes, a significant 2.7 fold increase compared to control females (1.6%; OR = 2.82, P = 4.64E-03). Mothers also carried marginally increased number of deletions >500 kb (2.3%) compared with control females (1.1%; OR = 2.07, P = 0.128). We observed similar trend at other thresholds of CNV size/gene content (Supplementary Table S6). The increase in CNV burden in the mothers was not due to underestimation of the CNV burden in controls or platform difference; because we have controlled for the effects of platform difference (Materials and Methods). In addition, the large CNV burden in patients’ fathers cannot be distinguished from control males; and male and female controls had similar CNV burdens. When comparing between the mothers and fathers that were genotyped by the same platform, there was a gradual increase among mothers with gene-affecting CNVs with increasing number of genes (Supplementary Table S11).

### Pathogenic CNVs

Using the criteria detailed in Materials and Methods, we identified 40 pathogenic CNVs in 39 patients (26 males, 13 females), including one patient with two such variants (Supplementary Table S10). The number of CNVs fulfilling each pathogenic criterion is shown as a Venn diagram in Fig. 1B.

**Figure 3:**
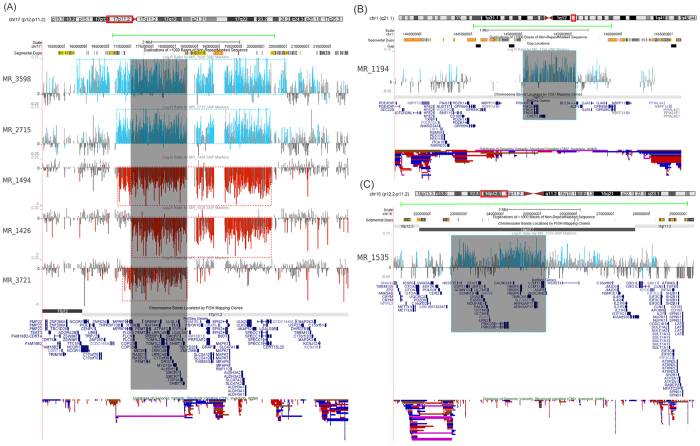
Atypical CNVs associated with known genomic disorders. (**A**) Five *de novo* CNVs (two duplications and three deletions) in the Smith-Magenis/Potocki-Lupski syndrome region. The minimal overlapping region encompasses two known haploinsufficient genes *RAI1* and *SHMT1*. (**B**) An atypical 1q21.1 duplication inherited from mother. The left boundary is uncertain due to gaps and segmental duplications in that region. Previous studies suggested a dosage sensitive gene *HYDIN2* in that region be the causative gene^26^,^29^. We tested the copy number of this gene using qRT-PCR, but did not find copy number gain of this gene. (**C**) A *de novo* 16p12.1 duplication partially overlaps the region of the known 16p11.2-p12.1 deletion/duplication syndrome, including *PLK1* gene. The duplication is proximal to 16p12.1 deletion syndrome^50^. In all cases, green lines above indicate the typical boundaries of genomic disorder-associated CNVs.

We found a total of 19 CNVs associated with known genomic disorders (Table 2). Most of them occurred *de novo*, except three (Xq28, 16p11.2, and 1q21.1) were inherited from mothers. CNVs at 16p11.2, 1q21.1 and 22q11.2 are known to have reduced penetrance and also occur at very low frequencies in controls. Mothers who carried 16p11.2 and 1q21.1 CNVs had reported stillbirth in their previous pregnancies. Fifteen of these CNVs were located in the hotspots of non-allelic homologous recombination (NAHR), which have typical boundaries defined by the flanking segmental duplications. We found three smaller CNVs showing atypical boundaries within NAHR hotspots (Fig. 3), which may help refine the candidate genes. As a proof of principle, we found a smaller 1.4 Mb *de novo* deletion carried by patient MR_3721 who showed classical sign of Smith-Magenis syndrome. The deletion co-localized with two other deletions and two duplications at the same locus; their smallest overlapping region contained the known disease genes *RAI1* and *SHMT1*(Fig. 3A). The recurrent 1q21.1 microdeletion/duplication have been associated with variable pediatric phenotypes including intellectual disability, autism, congenital heart defects, etc^26^. We identified a smaller 500 kb duplication in a prenatal case (MR_1194) with congenital absence of abdominal wall. The duplication contained three genes including *CHD1L* and *PRKAB2* (Fig. 3B). A previous study using patient-derived lymphoblast cell lines have documented functional anomalies of *CHD1L* and *PRKAB2* in 1q21.1 deletion syndrome^27^. A small deletion containing *CHD1L* has also recently been implicated in autism spectrum disorder^28^. The left boundary of this duplication was uncertain due to an assembly gap of human genome. Previous studies suggested *HYDIN2* gene within the gap be the causative gene^26^,^29^. We performed quantitative real-time polymoerase chain reaction (qRT-PCR) experiments and excluded the copy number gain of *HYDIN2*. However, we cannot exclude the increased copy number of other genes in this gap. The pathogenic effect of this duplication may also be due to functional alternations other than increased gene dosage (see Discussion). We also identified a male patient (MR_1535) carrying a *de novo* 2.3 Mb duplication located within 16p11.2-p12.1 deletion syndrome^30^ locus (chr16:22.75–25.02 Mb, hg18). The reciprocal duplication of 16p11.2-p12.1 deletion has only been recently established as a genomic disorder, with only a handful reported cases^30^,^31^,^32^. Our patient was 2 years old at diagnosis, showed speech delay, nystagmus, and mild craniofacial dysmophism (ptosis). The duplicated region contains 20 genes including *PLK1* (Fig. 3C), which has been suggested as the most likely dosage sensitive gene at this locus^32^. Close inspection of array signals (Supplementary Figure S5) revealed two nearby duplications, which most likely reflect a patient-specific inversion at this region.

Besides the CNVs associated with genomic disorders, we also identified 15 CNVs (11 deletions, 4 duplications) affecting large number of genes. All of them occurred *de novo* in patients. Known haploinsufficient (HI) genes were found in 6 deletions. In four cases, patients’ phenotypes well matched to the syndromic features caused by haploinsufficiency of *PAX6* (Aniridia, MIM:607108), *TCF4* (Pitt-Hopkins syndrome, MIM:610954), *FBN1* (skeletal/joint malformations, limb deformity; MIM:154700), and *TWIST1* (Saethre-Chotzen syndrome, MIM:101400). Four large duplications overlapped known HI genes including *ZEB2* (MIM:605802), *MBD5* (MIM:611472), *SALL4* (MIM:607343), *ENG* (MIM:131195); but evidence for the triplosensitivity remains to be established.

**Figure 4:**
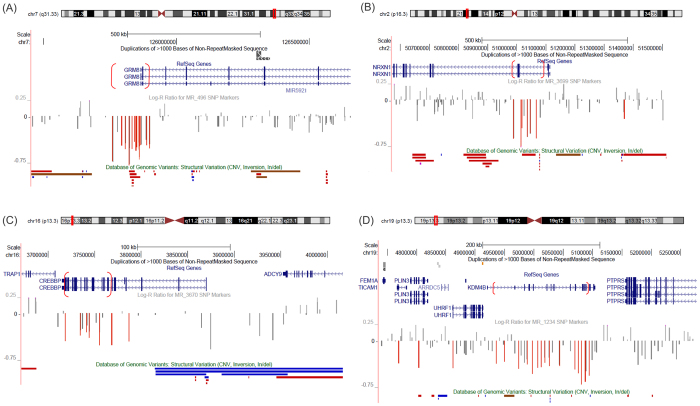
Small deletions that disrupt coding exons of a single disease gene. (**A**) *GRM8*, (**B**) *NRXN1*, (**C**) *CREBBP*, and (**D**) *KDM4B*. The approximate CNV boundaries are shown as parentheses superimposed on gene structures. Deletions of *GRM8* and *NRXN1* were maternally transmitted; deletions of *CREBBP* and *KDM4B* occurred *de novo*.

In addition to very large CNVs or CNVs associated genomic disorders, we were also able to identify several small deletions (<500 kb and affecting <= 3 genes) (Table 3). These include single-gene exonic deletions in *NRXN1*, *GRM8*, *CREBBP* (Rubinstein-Taybi syndrome, MIM:180849), *KDM4B*, and *DMD* (Fig. 4). Three CNVs including duplication of *PPP3CC*, deletions involving *DYNC1I1* and *DLGAP2* were considered likely pathogenic, which have been previously reported in a few NDD patients.

### Female Protective Model

**Figure 5:**
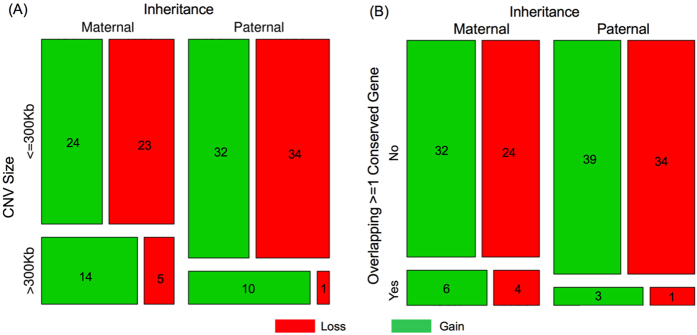
The maternal transmission bias of ultra-rare CNVs in patients. The mosaic plots show the number of autosomal CNVs stratified by inheritance status (maternal or paternal), size (>or <=300 kb), and type (gain or loss). The area of each rectangular partition of the square is proportional to the number of CNVs fall in each classification. (**A**) CNVs larger than 300 kb tend to be transmitted from mothers as compared with fathers (p < 0.05 by Fisher’s Exact Test (FET)). (**B**) Maternally transmitted CNVs are also more likely to overlap with the coding exons of evolutionary conserved genes, which are defined by the top 10% of residual variation intolerance score^88^ (p < 0.1 by FET).

Interestingly, we noted that all inherited pathogenic CNVs were identified in male patients and maternally inherited (Tables 2 and 3), only two of which were on chromosome X. Among ultra-rare autosomal CNVs identified in patients, inheritance could be determined with high confidence for 143 of them. Overall, the maternal transmission rate was 46.2% (66 out of 143), which was not significantly different from the proportion of mothers in the parents (252 of 510) (OR = 0.83, P = 0.38). However, there was a marginal excess of maternally inherited CNVs that were >300 kb (OR = 2.33, P = 0.047) and CNVs that contains at least one evolutionary conserved gene (OR = 3.16, P = 0.059) (Fig. 5). No such bias was observed in common CNVs (data not shown).

To further explore the maternal transmission bias, we also examined rare inherited CNVs identified in a large clinical cohort of intellectual disability and/or multiple congenital anomalies from a published study^19^. Data on inheritance were available for 1065 CNVs, including 584 inherited autosomal CNVs in 529 patients. Overall, 330 (56.5%) of them were maternally inherited (P = 1.88E-03). Because parents were not genotyped in that study, rare CNVs might be subject to selection before parental testing. To account for potential ascertainment bias, we compared the inheritance rate at different CNV sizes, gene contents, and HI scores^33^ (Supplementary Table S12). Maternal inheritance gradually increase with the increase of HI score threshold, and was significantly enriched for CNVs with HI score >12 (25 maternal vs. 5 paternal; 85.3% maternal rate) than for CNVs with HI score <= 0 (56% maternal rate) (OR = 3.83, P = 4.9E-03). The maternally inheritance rate was also marginally higher for larger CNVs (>2.5 Mb) and CNVs affecting more genes (>25 genes). To further predict deleteriousness, we compiled 321 most likely dosage sensitive genes (harboring autosomal dominant disease-causing LoF mutations) implicated in developmental disorders from DDG2P database^34^. Significantly more maternally inherited CNVs overlap at least one such genes (41 maternal vs. 14 paternal, OR = 2.43, P = 4.28E-03). Therefore, potential pathogenic CNVs were enriched in maternally inherited alleles even after accounting for overall increase of maternal transmission.

We have demonstrated an increased CNV burden in the mothers of MR/DD patients compared to both controls and the fathers of the patients. It also suggests that in addition to transmitted CNVs, un-transmitted CNVs in mothers may also be enriched for pathogenic alleles. So we applied the same criteria to identify potential pathogenic CNVs in parents that are not found in patients. Six (likely) pathogenic CNVs were found (Table 4), including 16p13.11 deletion, deletions of *CUL3*, *BMP4*, *WHSC2*, *KANK1*, and a duplication of *CNTN4*-*CNTN6*. Notably, all of them were carried by the mothers.

The identification of pathogenic CNVs, both transmitted and un-transmitted, in unaffected mothers, can be explained if females are more tolerant than males to deleterious mutations. Under this hypothesis, manifestation of MR/DD for females would require a higher burden of deleterious mutations. Consistent with this, we found that pathogenic CNVs identified in female patients affected a significantly higher number of genes (median: 49) than those in male patients (median: 25; P = 0.022). The increased number of affected genes was not due to duplications affecting more genes than deletions, as only one pathogenic duplication was found in female patients.

### Additional Findings

In addition to CNV calling, SNP array data can be further exploited to identify long runs of homozygotes (ROH), constituent uniparental disomy (UPD), and mosaic structural abnormalities^35^.

By analyzing the Mendel errors in trios, one female patient (MR_1369) was found to have unusually large number (569) of Mendelian transmissions on chromosome 15. Over 99.9% of her genotype on this chromosome exactly matched to her mother without loss of heterozygosity, indicating that it was caused by maternal chromosome 15 heterodisomy. The patient’s phenotype confirmed the diagnosis of Prader-Willi syndrome. We also searched ROH segments, which may harbor recessive disease-causing mutations. By comparing to parental genotypes, the possibility of uniparental isodisomy was excluded for all segments >10 centi-Morgans (cM) in patients of trios. One male patient (MR_2559) was found to have total ROH length over 300 cM. The estimated kinship coefficient of his parents was ~0.13, consistent with first cousin mating. In addition, we found three other patients with total ROH greater than 50 cM, which might result from recent inbreeding. None of them carried pathogenic CNVs; neither did we found homozygous CNVs in their ROH segments. Further sequencing studies will be needed to identify the recessive mutations. A burden analysis showed that the proportion of patients with long ROH length was not significantly higher than that of parents and controls (Supplementary Table S13), which was expected since consanguineous families were not preferentially ascertained in this study. We did not find detectable mosacism in patients, but identified one mosaic segmental UPD from 11p15.3 to p-terminus in a parent with unknown origin (Supplementary Figure S8). Segmental parental UPD for 11p15.5 is a known cause of Beckwith-Wiedemann syndrome in 10–20% of patients^36^. The clinical significance of the finding was unclear.

Despite the low probe density (~300 k), we were able to identify a number of single gene disruptions in this study, including 3 single gene and 24 exonic deletions in patients (Supplementary Table S5). To opportunistically identify additional gene disrupting CNVs, we implemented a workflow of targeted rare CNV genotyping^37^ to search for small gene disrupting CNVs that may be missed by the standard calling algorithm (Supplementary Methods). Exon regions spanned by five informative markers in over 2000 candidate genes were interrogated to look for outliers in the signal intensities across the sample. Samples with consecutive probes of low or high intensities are candidate carriers of a rare CNV. This method achieved 85% sensitivity in recovering the ultra-rare CNVs identified in the clinical cohort (positive for 58 out of 68 CNVs that covered targeted regions; Table S8, Table S9). After a series of QC filter, prioritization and validation, we identified a small deletion in a male patient (MR_3590), disrupting three genes including the coding exon 1 of *HIRA* gene (Supplementary Figure S9) within the 22q11.1 deletion (DiGeorge) syndrome region. *HIRA* encodes for a histone chaperon, and is considered as a primary candidate for DiGeorge syndrome^38^. It has a predicted HI probability of 0.94^33^. No gene-disrupting CNV other than the typical 22q11.2 deletion was observed in controls; and loss-of-function (LoF) variants in this gene were also extremely rare (based on ExAC database). The patient showed mental retardation, speech delay, and hyperactivity. The clinical significance of this deletion remains to be evaluated with new mutations in additional patients.

## Discussion

### Implications for CNV Interpretation

We adopted three overlapping criteria for evaluating the pathogenicity of CNVs. Those criteria are dynamic since our knowledge about genomic disorders and gene dosage sensitivity are constantly updating. This is especially the case for CNV-disease associations outside NAHR hotspots, because rarity of the events entails the screening on clinical cohorts with very large sample sizes to establish their disease causality.

Among large deletions identified in patients, we found a female patient (MR_3067) with a 3.7 Mb deletion at 4q21 locus encompassing candidate genes *PRKG2* and *RASGEF1B*. The patient showed gross developmental delay since birth, speech impairment, and generalized tonic-clonic seizure since 2 years old. Although patients with overlapping deletions at 4q21 locus have been reported, the locus only gained genome-wide significance in recent studies^22^,^23^. Haploinsufficiency of *MBD5* gene was known to be responsible for the phenotypes of 2q23 micro-deletion syndrome^39^. The triplosensitivity of this same gene has only been suggested recently^40^. We identified a large duplication encompassing 2q23 deletion syndrome region in a female patient (MR_1191) with speech delay and dysmorphic craniofacial features.

The evaluation criteria were based on the presumed functional consequence of CNVs on affected genes. Throughout the study, we consider a CNV affects a gene if it overlaps its CDS region. While it may be obvious that deletion leads to haploinsufficiency, the consequences of duplication are less clear. In addition to increase the dosages of encompassing genes, duplication may also disrupt genes at its insertion site^41^, or create chimeric genes at breakpoints^42^. Several duplications that partially overlap the coding sequences known HI genes were noted, including a 190 kb *de novo* duplication overlapping *FBN1* and *SLC12A1*, and a 750 kb duplication overlapping *PDE4D* (Supplementary Table S8). The functional consequences cannot be determined until the breakpoints are resolved. Deletions that affect noncoding or regulatory sequences may also causes haploinsufficiency due to the changes in gene transcription or translation, but functional assays would be required for validation.

Additional factors are also considered in practice when evaluating the pathogenicity of CNVs, including inheritance, frequency in natural populations, and recurrence in patients, which are discussed below.

### Inheritance

We identified and validated a total of 41 *de novo* CNVs in 39 patients, 33 of them were judged as pathogenic and 2 likely pathogenic, reinforcing the notion that not all *de novo* CNVs are disease causing^18^. The remaining six *de novo* CNVs in five (1.7%) patients were of unknown clinical significance, four of which did not overlap any coding gene. The carrier rate is consistent with previous reported *de novo* CNV rates (1~2%) in healthy control trios^43^,^44^,^45^. While most of the pathogenic *de novo* CNVs were large, we did find two *de novo* exonic deletions. One notable example is *KDM4B*, a lysine-specific demethylase. The LoF variants in this gene and several members of the same family (*KDM3A*, *KDM5B*, *KDM6B*) have recently been associated with autism in a large scale genetic investigation^46^. Other members of the KDM family were also been identified as the disease genes for syndromic mental retardation, including *KDM5C* (MIM:314690) and *KDM6A* (MIM: 300128). We also identified a *de novo* duplication encompassing *PPP3CC*, which encodes a catalytic subunit of calcineurin, a protein phosphatase involved in the downstream regulation of dopaminergic signal transduction. Similar duplications were observed in at least two other patients with affective and cognitive disorders^47^,^48^. And the gene has recently been identified as a putative modulator of antidepressant response^49^. The *de novo* occurrences in the above cases add further support for the causal involvement of dosage sensitivity of *KDM4B* and *PPP3CC* in NDD.

Our study also confirms the pathogenic roles of inherited CNVs, not restricted to chromosome X, which are transmitted from asymptomatic parents because of incomplete penetrance. The penetrance of some pathogenic CNVs may be mediated by the presence of a second large CNV^50^,^51^, and/or due to gender-specific liability thresholds where females are predicted to carry a more penetrant risk variant load to be affected^52^. We found strong evidence to support for the latter. The findings of our study can be explained a simple transmission model of DD/MR which was previously proposed for autism^53^. Families with affected children can be classified into at least two types. Patients in low-risk families were caused by *de novo* mutations that mainly originate from mutations in paternal germline, with high penetrance in male and low penetrance in female offspring. The unaffected females carriers of the causative mutation in turn transmit the mutation in a dominant fashion to their offspring. It also suggests that inheritance of a CNV be incorporated into the review process to aid the evaluation its clinical significance.

### Population Frequency

In this study, we focused on ultra-rare CNVs, which had frequency <0.1% in natural population. They are expected to enrich for highly penetrant alleles that are under strong purifying selection. We also demonstrated that CNVs within 0.1~1% frequency range did not contribute to the large CNV burden in patients. However, we may have missed CNVs that only moderately increase the disease risk.

So we checked the population frequencies of CNVs associated with known genomic disorders (Supplementary Table S15). We found all except one (15q11.2 BP1-BP2 deletion) appeared <0.1% in controls, which is also consistent with previous reports^22^,^23^,^54^. Given CNVs outside NAHR hotspots are less frequent, applying population frequency filter of 0.1% would unlikely miss pathogenic CNVs with high penetrance. The 15q11.2 BP1-BP2 deletion is proximal to Prader-Willi/Angleman syndrome deletion separated by a genome assembly gap. The deletion has been associated with schizophrenia^55^, developmental delay^56^, congenital heart defects^57^, and cognitive abilities^58^ but with low penetrance. The frequency of the deletion in our control databases is 0.35%, similar to previous reports in WTCCC data^59^, but higher than some other studies^22^,^58^,^60^. We found two cases with the deletion; both also carried the Angelman syndrome deletion. Most likely they represent a single large deletion spanning from BP1 to BP3 (Supplementary Figure S10).

Theoretically, the population frequency of a disease-causing mutation is determined by the inheritance mode, selection coefficient, genetic drift, and locus-specific mutation rate. A relatively high population frequency of 15q11.2 deletion can be explained by a combination of its moderate penetrance^22^ and a high rate of rearrangement in NAHR hotspots. The association of this deletion with NDD in Chinese remained to be evaluated in future studies.

### Recurrence in Patients

Because of the stringent filtering procedure and a relatively small sample size, most of the ultra-rare CNVs identified in patients are private except for a few cases in NAHR hotspots. We also searched for overlapping events outside NAHR hotspots and identified several cases of overlapping CNVs and intersecting genes (Supplementary Figure S11).

**Figure 6:**
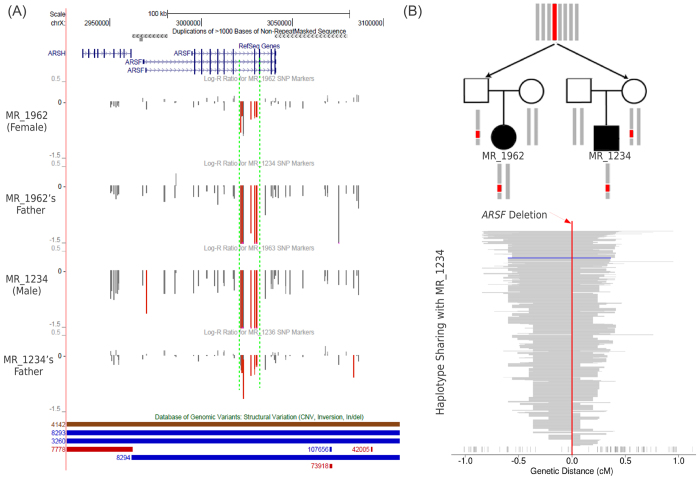
Shared haplotype background for a recurrent deletion in two patients. (**A**) The genome browser tracks show the gene structure, log-R ratios of SNP array probes, and CNVs from Database of Genomic Variants at this region. The deletion omitting the exon 8 of *ARSF* gene was identified in one male patient inherited from mother and in one female patient inherited from father, with identical boundary observed in both cases. The same deletion is absent in other unrelated samples from in-house and public databases. The deletion carriers are unrelated as confirmed by whole-genome SNP genotypes. (**B**) If the deletion originated from an ancestral mutational event several generations ago, then the mutation carriers are expected to share the same chromosome background identical by descent from the recent most common ancestor (shown in red). Because males are hemizygous on chromosome X, the haplotype background of this deletion can be determined. (C) To quantify the shared ancestry around the deletion locus, we statistically inferred 624 unrelated haplotypes from trios in the cohort. Haplotype sharing with the male patient who carried the deletion was visualized. The extent of haplotype sharing was measured by allele matching starting from the deletion point toward left and right until the first marker showing the mismatched allele. Each line represents an unrelated haplotype, sorted by the shared haplotype length. The second deletion haplotype in the female patient is highlighted in blue. SNPs used to define haplotypes are marked below. Positions were relative to the deletion locus in centiMorgan (cM). The genetic length of shared haplotype background of the deletion is about 0.75 cM.

Notably, we found an exonic deletion of *ARSF* at Xp22 shared by two patients: one was carried by a male patient inherited from his mother, the other was carried by female inherited from her father. The deletions found in two patients had exactly the same boundary (Fig. 6A). Both parents were unaffected. The same deletion was not observed in controls or public databases. Since most CNVs outside NAHR hotspots were generated from replication-based mechanisms^61^ presumably with very low mutation rates, rare CNVs with recurrent boundaries outside hotspot regions are most likely generated by ancestral mutational events (Fig. 6B). Haplotype sharing analysis shows that the two deletions shared a 0.75 cM haplotype identical by descent (Fig. 6C). It suggests that the deletion is likely a local polymorphism, not subject to purifying selection. The absence of this CNV in controls may be caused by the lack of probe coverage at this locus. In an early resequencing study of X-linked mental-retardation pedigrees, the *ARSF* gene was initially nominated as a candidate but was later rejected because LoF variants were also observed in healthy males^62^.

While recurrent disruption of genes exclusive to patients was commonly used to lend further support on pathogenicity, our case suggests that caution should be exercise to interpret rare CNVs with recurrent boundaries outside NAHR hotspots.

### Comparison with Previous Studies

To compare the CNV burdens with previous studies^22^,^23^, we considered all low-frequency variants (<1% population frequency). For CNVs >500 kb, we found a total of 64 autosomal CNVs in 61 (21.3%) patients, compared to 592 such events in 571 (9.7%) in controls. For CNVs >750 kb, the carrier rate was 15.0% and 5.5% for cases and controls respectively. The excess of patients was 11.6% with CNVs >500 kb and 9.5% for CNVs >750 kb, which were close to but slightly lower than the previous report (13.5% with >500 kb, and 12.7% with >750 kb). This was probably due to the exclusion of patients with subtelomeric aberrations. Previous studies also report an increased CNV burden in patients with multiple congenital anomalies (MCA)^19^,^22^. Among 270 patients with detailed clinical information, 13.7% of them were considered having MCA (showing at least two signs of brain malformations, gross craniofacial dysmorphology, cardiac defects, and neurological deficits). And we observed slightly higher number of patients with MCA carrying CNVs affecting at least 8 genes (15.8%) as compared with patients without MCA (12.1%). But our sample size is limited to reach a significant conclusion (OR = 1.4, P > 0.5).

Including the case of chromosome 15 UPD, we found 41 pathogenic structural variants in 40 (14%) patients. If likely pathogenic CNVs were included, the number increases to 44 variants in 42 (14.6%) patients. We also identified pathogenic CNVs, including 8 transmitted and 4 un-transmitted, in 12 (2.4%) unaffected parents all of whom were patients’ mothers. The reported diagnostic yield range from 10% to 20%^63^, depending on many factors including sample ascertainment, genotyping platform, analytical methods, and evaluation criteria. The diagnostic yield of our study fall well within this range, and is higher than the observed large CNV burdens due to a number of small pathogenic CNVs (Table 3). Resolution can be further improved by using high-density exon targeted arrays^9^ or exome sequencing^64^.

A higher proportion of male MM/DD patients has long been noted in epidemiology studies^65^, which is also evident in our patient cohort with 46% excess of males patients. The increased proportion of male patients cannot be fully explained by hemizygous causal mutations on chromosome X^66^. A “female protective model” has been proposed to explain the male excess. While the simple model cannot capture every aspect of gender bias in NDD^67^, it has gained support from a number of studies, mostly in autism which showed highest male/female ratio (7:1). It has been demonstrated that: (1) female patients are more likely to have highly penetrant mutations than male patients^45^,^52^,^68^ (2) inherited disease-causing mutations are more likely transmitted from mothers^68^ and (3) mothers of the patients have higher burdens of deleterious mutations^69^. All of them were also supported by our data. We made a strong case for the maternal transmission bias of pathogenic alleles by showing that pathogenic or likely pathogenic CNVs with strong literature support identified in parents were all carried by mothers, only two of which were on chromosome X. This could also partly due to our ascertainment criteria that included only sporadic cases with healthy parents. Some previous studies also reported inherited pathogenic CNVs transmitted from mildly affected fathers^70^.

Burdens of inherited CNVs have also previously been investigated in autism by comparing transmitted CNVs between probands and unaffected sibs^44^,^71^, but only weak or no evidence of disease association was found. It is possible that most inherited rare variants are likely benign, only a few are disease causing; burden analysis has limited power if signals are buried in large amount of noise. Inherited CNVs of DD/MR in two other large data sets (ISCA, Signature Genomics) were also previously analyzed^68^. Consistent with our analysis of ref. 19, stronger maternal transmission bias stood out after the putative pathogenic CNVs were being enriched. Some studies also reported an overall maternal transmission bias of small exonic CNVs^64^, and higher CNV burden in females sampled from general populations^69^, both of which were not observed in our study. Future studies with increased resolution and expanded populations will be needed to evaluate these findings.

At each individual locus of genomic disorders, most commonly observed CNVs in Europeans were also found in Chinese patients of NDD (Table 2, Supplementary Table S15). But some population-specific locus does exist. A notable example is 17q21.3 micro-deletion syndrome. A local inversion polymorphism at this locus put the segmental duplications in the same orientation, predisposing this locus to NAHR^72^. As this inversion is almost exclusively found in Europeans, we did not observe similar patients in Chinese and other non-European populations.

Additional population specific loci may exist, because segmental duplications are highly variable between individuals genome-wide^73^ and thus may also likely differ across populations. We compared the CNV frequencies of our case/control cohorts in NAHR hotspots reported previously^22^ (Supplementary Table S16). A number of locus showing population differentiations can be noted, although it can also be attributed to platform difference. Further studies will be needed to better characterize population differences in the duplication architecture and understand their consequences on CNVs in disease.

## Materials and Methods

### Patients Ascertainment

The clinical cohort used in this study was selected from MR/DD trios collected by Department of Pediatrics in Peking University First Hospital. Informed written consent was obtained from the parents. The clinical manifestation of the patients included a wide range of neurodevelopmental phenotypes, typical of clinical diagnostic setting. Patients were ascertained to exclude history of prenatal brain injury, toxication, hypoxia, central nervous system infection, or cranial trauma. They also showed no evidence of recognizable inherited metabolic disorder or specific neurodegenerative disorders by brain imaging and blood/urinary metabolic screening. For patients eligible for intellectual assessment, only moderate to severe cases (IQ < 55, assessed with Gesell Developmental Schedules or Wechsler Intelligence Scale for children) were included. Sanger sequencing on *FMR1* gene had been performed to exclude causative mutations for male patients; and clinical features of Rett syndrome were excluded for female patients. All of them had normal karyotype and negative of subtelomeric aberrations screening reported previously^74^.

Genomic DNA was extracted from peripheral blood for each index patient and his or her parents. DNA samples of 289 patients and 510 parents were genotyped using Illumina CytoSNP12 bead arrays, which had a total 299,671 probes. The research was approved by the ethical committees of Peking University First Hospital and National Research Institute of Family Planning. The methods were carried out in accordance with the approved guidelines.

### Control Cohorts

Control samples included in this study were taken from previously published genome-wide association studies (GWAS) collected by two institutions in China (Supplementary Table S1B). The Shanghai Institute of Hypertension (SIH) cohort (n = 902) was recruited for a case-control study of essential hypertension. The GWAS summary statistics were previously included in the GWA meta-analysis of blood pressure by Asian Genetic Epidemiology Network^75^. The Anhui Medical University (AMU) cohort (n = 5366) was composed of cases of three autoimmune diseases and shared controls. The samples were used in previous GWAS of psoriasis^76^, systemic lupus erythematosus^77^, and vitiligo^78^. All GWAS samples were genotyped using Illumina HumanHap 610 k bead array. Neurodevelopmental disorders were not screened for the GWAS subjects. Although these subjects are not representative of general population, they can be served as controls because no evidence has so far been found that rare and large CNVs are associated with these diseases. But even if large CNVs do increase the risk of some disease, this would only make our comparison with MR/DD more conservative.

### Data Processing

We implemented and validated a rigorous computational workflow for CNV calling, quality control, filtering and interpretation (Supplementary Figure S1, details are given in Supplementary Methods). Briefly, we used PLINK^79^ for genotype management and analysis. After SNP-based QC, genotypes were used to identify duplicated samples, and to verify familial relationships, reported genders, and population ancestry. Genotypes were also used to infer constituent UPD and long ROH. After SNP genotype-based QC, we performed further QC on samples based on array intensities before CNV calling. CNVs were called based on log-R ratio and B-allele frequency signals using PennCNV^80^. High confidence CNV calls were subject to a series of filters to enrich for causal variants. The steps included the exclusion of known CNPs, CNVs of the same type that occurred at least five times in 5866 population controls, and CNVs detected in at least two other unrelated parents. The resulting ultra-rare CNV calls were subject to iterative QC, manual curation, and experimental validation to generate the final CNV list. The inheritance status of CNVs found in patients was initially determined by comparing to the unfiltered CNV segments in parents, then subject to manual curation. *De novo* origin in 5 cases where at least one parent was not available for testing was assumed based on the absence in one parent and their well-established causal involvement in severe and fully penetrant phenotypes.

To identify smaller exonic CNVs that might be missed by standard calling algorithm, we also started with a list of known/candidate disease genes, and defined target regions that cover the coding exons and contain enough informative SNP probes. Then, targeted rare CNV genotyping were performed to search for additional small CNVs^37^. Mosacisms were called using MAD package^81^.

### CNV Validation

Genomic DNA was extracted from peripheral blood leukocytes using standard methods (QIAamp DNA Mini Kit, Qiagen, Hilden, Germany). The exon regions of identified genic CNVs were targeted for primer design. Primer sequences of candidate genes were designed based on sequence data obtained from the NCBI database using Primer Express software 3.0 (Applied Biosystems).

The targeted regions were amplified in patients, parents (when available), and 12 unrelated healthy control individuals with matched ancestry by qRT-PCR with a set of gene-specific primers (available upon request). The gDNA was used as template in qRT-PCR reactions with SYBR^®^ Green PCR Master Mix (Takara Biotechnology (DaLian) Co. LTD) and performed on 7000 real-time PCR system (Applied Biosystems). The quantification of the target was normalized to an assay from chromosome 21, C2, and the relative copy number (RCN) was determined on the basis of the comparative ΔΔCt method with a normal control DNA as the calibrator^82^. The experiments were repeated three times. A ~0.5-fold RCN and a ~1.5-fold RCN were used for deletion and duplication, respectively.

### CNV Burden Analysis

To analyze the burden of ultra-rare and large CNVs in patients and parents, we need an accurate estimate of the baseline rate in control populations. CNVs in controls were first subject to the identical filtering process as done for the clinical cohort. Namely, we excluded CNVs whose 50% of the region overlapped CNP regions, or were covered by CNVs of the same type found in at least 5 unrelated controls or 2 parents. Due to platform differences, CNV calls in controls were further required to cover at least 10 markers on CytoSNP12 array. We also checked that all ultra-rare large CNVs found in the clinical cohort also had enough probe coverage (>=10 probes) on HumanHap 610 k array. CNVs >=500 kb or overlapping the coding exons of >=8 refSeq coding genes (over 90% of them >=250 kb) were subject to manual curation by the analyst with proven accuracy validated by experiments (Supplementary Methods). The manual curation were applied uniformly to the CNVs retained after automated filtering to correct for erroneous boundaries, and to reject false positives caused by abnormally behaving probes, CNVs artifacts in regions of segmental duplications or genome assembly gaps, etc. All comparisons between cases and controls were made on the curated CNV set, for which we believe should be least influenced by the platform differences.

We also analyze the burden of low frequency (0.1~1%) large CNVs. The low-frequency set was derived by using different filtering criteria; namely, CNVs were excluded if 50% its region overlap CNVs of the same type in at least 59 unrelated controls and 6 unrelated parents. We then manually reviewed signal plots of all low frequency CNV calls that were not in the ultra-rare CNV set.

### Parental Origin (POO) of *De novo* CNVs

To determine the POO of *de novo* CNVs, we first generated CNV specific genotype calls for the offspring using PennCNV (infer_snp_allele utility). SNP markers informative of POO were then identified by comparing with the genotypes of parents. For example, when a father and mother’s genotypes at a SNP marker are AA and AB, respectively, then if offspring carry a duplication whose genotype call is ABB, then we can infer this *de novo* duplication happened on the maternal chromosome. Similarly, if the offspring carry a deletion whose genotype call at that marker in B, then we can infer the deletion happened on the paternal chromosome. For complete trios, we inferred the POO from all such informative SNPs with the CNV segment; a binomial p-value was assigned to test the predominance of paternal or maternal origin (against the 50% equal chance). When p < 0.01, POO of the *de novo* CNV was determined by the majority vote. The POO of small *de novo* CNVs without enough informative SNPs could not be determined. In four rare cases, only genotypes of one parent were available, but the *de novo* origin was presumed based on the well-established causal involvement in severe and fully penetrant phenotypes. The CNV-specific genotype was compared to the genotypes of the available parent, to check whether the CNV is consistent with the mutation on that parent. In case of large number of consistencies, CNV was presumed to originate from mutation in the other parent. Because all four CNVs had >100 informative SNPs, the POO determined by this heuristic approach should be accurate.

### CNV Interpretation

To judge the pathogenicity of CNVs, we adopted the following criteria in spirit of ACMG recommendation^83^ and an evidence-based review procedure^20^. A CNV is judged causal if it overlaps at least 50% of the critical region of known genomic disorders matched for the copy numbers; or deletes the CDS regions of known haplo-insufficient genes or duplicates known triplo-sensitive genes^84^,^85^. Typical phenotypes of well known syndromes were checked with clinical records to confirm the diagnosis. If no such genes was found, we subjectively judge a CNV as pathogenic if it overlaps the CDS regions of a large number of genes: >=15 genes for deletions and >=20 genes for duplications. The gene number threshold were determined empirically such that >99% of CNVs detected from population-based controls should fall below these levels (Supplementary Figure S7). We also considered a CNV to be probable pathogenic, if it overlaps critical regions or dosage sensitive genes recently emerged from large-scale genetic studies of NDD with strong statistical significance or with convincing experimental evidence. CNVs were judged as likely pathogenic, if it were also reported in patients with similar phenotypes. The remaining was considered as unknown clinical significance. CNVs judged as pathogenic or probable pathogenic are considered as pathogenic variants throughout the paper.

### Haplotype Analysis

To shed light on the evolutionary origin of the recurrent *ARFS* exonic deletion at Xp22 (Supplementary Figure S11G), we analyzed its SNP haplotype background. Genotypes from 218 complete trios centered around the deletion (+/−1 cM) were extracted (physical position: chrX:2720840-3628132, hg18 assembly). We only included common SNPs with MAF >0.05. Male heterozygotes and Mendel errors were set to missing. If a sample had more than 4% missing genotypes, then the complete trios was excluded from analysis. In trios consisting of father-mother-son, hemizygous genotypes of the son were used for phasing the genotypes of mother; and father’s haplotype was assumed known. This resulted in 143 phased mothers and 143 phase known fathers, or 429 unrelated haplotypes in total. In trios consisting of father-mother-daughter, father’s genotypes were treated as diploid homozygotes. We then used 429 resolved haplotypes to “guide” the phasing of genotypes of 65 father-mother-daughter trios using Beagle version 3.3.2^86^,^87^. A total of 624 unrelated haplotypes were inferred. The shared haplotype background was determined by allele matching extending from the deletion locus toward left and right sides until the first marker with mismatched allele was encountered.

## Additional Information

**How to cite this article**: Wang, B. *et al*. CNV analysis in Chinese children of mental retardation highlights a sex differentiation in parental contribution to *de novo* and inherited mutational burdens. *Sci. Rep.* **6**, 25954; doi: 10.1038/srep25954 (2016).

## Supplementary Material

Supplementary Information

Supplementary Information

## Figures and Tables

**Table 1:** Burden of ultra-rare and large CNVs in MR/DD patients, their parents, and controls.

		All Samples			Females			Males		
		All	Gains	Losses	All	Gains	Losses	All	Gains	Losses
Length >500kb										
Patients	Number of samples with CNVs	53	24	29	20	8	12	33	16	17
	Percent of samples with CNVs	18.5%	8.4%	10.1%	20.4%	8.2%	12.2%	17.5%	8.5%	9.0%
	Fold change (vs. controls)	2.89	1.59	9.18	3.24	1.61	11.09	2.69	1.55	9.00
	*p*-value	1.7E-11	3.2E-02	4.4E-17	4.3E-06	1.7E-01	7.9E-09	1.2E-06	1.0E-01	7.8E-10
Parents	Number of samples with CNVs	37	30	7	20	14	6	17	16	1
	Percent of samples with CNVs	7.3%	5.9%	1.4%	7.8%	5.4%	2.3%	6.7%	6.3%	0.4%
	Fold change (vs. controls)	1.14	1.11	1.27	1.24	1.06	2.09	1.03	1.15	0.40
	*p*-value	4.5E-01	5.4E-01	5.1E-01	3.5E-01	7.7E-01	1.3E-01	8.9E-01	5.6E-01	5.1E-01
Controls	Number of samples with CNVs	374	310	64	193	158	35	181	152	29
	Percent of samples with CNVs	6.4%	5.3%	1.1%	6.3%	5.1%	1.1%	6.5%	5.5%	1.0%
Affecting at least 8 Genes										
Patients	Number of samples with CNVs	36	12	24	13	2	11	23	10	13
	Percent of samples with CNVs	12.5%	4.2%	8.4%	13.3%	2.0%	11.2%	12.2%	5.3%	6.9%
	Fold change (vs. controls)	7.35	3.50	16.80	8.31	1.82	22.40	6.78	4.08	17.25
	*p*-value	1.0E-18	3.7E-04	3.2E-19	1.9E-08	2.9E-01	7.0E-11	2.1E-11	5.6E-04	5.0E-10
Parents	Number of samples with CNVs	12	8	4	11	7	4	1	1	0
	Percent of samples with CNVs	2.4%	1.6%	0.8%	4.3%	2.7%	1.6%	0.4%	0.4%	0.0%
	Fold change (vs. controls)	1.41	1.33	1.60	2.69	2.46	3.20	0.22	0.31	0.0
	*p*-value	2.8E-01	4.0E-01	3.1E-01	4.6E-03	3.1E-02	5.3E-02	1.2E-01	3.7E-01	6.2E-01
Controls	Number of samples with CNVs	97	70	27	48	33	15	49	37	12
	Percent of samples with CNVs	1.7%	1.2%	0.5%	1.6%	1.1%	0.5%	1.8%	1.3%	0.4%

Total number of patients: n = 287 (189 males, 98 females), total number of parents: n = 510 (252 males, 258 females), total number of controls: n = 5866 (2780 males, 3086 females). Only CNVs on autosomes are included in the burden analysis. Fold change: fold change in proportion of samples carrying large CNVs as compared with controls. P-values were calculated by two-sided Fisher’s Exact Test. Gene content of a CNV is defined by the number of refSeq coding genes whose coding sequences overlap with the CNV segment.

**Table 2:** Pathogenic CNVs associated with known genomic disorders.

Cytoband	Syndrome	OMIM ID	Is NAHR Hotspots?	CNVs in patients	CNVs in controls
1q21.1	1q21.1 deletion/duplication syndrome	612475	Y	1 atypical dup (inherited from mother)	3 dups, 1 del
2q32-q33	2q32-q33 deletion syndrome (*SATB2*)	612313	N	1 large del	
7q11.2	Williams-Beuren syndrome/WBS Duplication	194050, 609757	Y	1 del, 1 dup	
15q11.2-q13.2	Prader-Willi and Angelman syndrome/15q11.2-q13.2 duplication syndrome	176270,105830	Y	2 dels^i^	
15q24	15q24 deletion syndrome	613406	Y	1 del	
16p11.2	16p11.2 micro-deletion/duplication syndrome (*SH2B1*)	613444, 614671	Y	1 del (inherited from mother)	2 dels, 1 dup
16p12.1	16p11.2p12.1 deletion syndrome	613604	Y	1 atypical dup	
17p13.3	Miller-Dieker lissencephaly syndrome (*PAFAH1B1*)	247200	N	1 large del	
17p11.2	Smith-Magenis syndrome/Potocki-Lupski syndrome	182290, 610883	Y	3 dels (1 smaller atypical), 2 dups	
22q11.2	DiGoerge, Velocardiofacial syndrome/22q11.2 duplication syndrome	188400, 192430, 608363	Y	2 dels	1 del, 3 dups
22q13.3	Phelan-McDermid deletion syndrome (*SHANK3*)	606232	N	1 large del	
Xq28	*MECP2* duplication syndrome	300260	N	1 large dup (inherited from mother)	

NAHR hotspots: genomic disorders that defined by the hotspots of non-allelic recombination mediated by segmental duplications. CNVs are required to have 50% reciprocal overlap with the NAHR hotspots, or cover the critical region defined by the syndrome with matched copy number. Unless otherwise noted, CNVs in patients are of *de novo* occurrence.

^i^Both *de novo* CNVs originated on the maternal allele. Patients were diagnosis as Angelman syndrome.

**Table 3:** Patients carrying small (likely) pathogenic CNVs (affecting <15 genes).

Patient (Gender)	Age^i^	Phenotype	Cytoband	Start-End (kb)	Size (Mb)	Copy number	Origin	Num of Genes	Candidate Genes	Pathogenicity
MR_3670 (Male)	12M	Growth retardation, microcephaly, cryptorchidism, hypotonia, open foramen ovale, left had joint deformity, elevated right side of sternum, eye vascular tumor	16p13.3	3,710.7–3,941.9	0.23	Loss	*De novo*^ii^	1	*CREBBP* (Exonic)	Confirmed: Rubinstein-Taybi syndrome (OMIM:180849)
MR_211 (Female)	1Y1M	Delayed psychomotor development with prominent speech delay, microcephaly, hypotonia, no hearing problem, stereotyped movement	Xp11.4	41,403.1- 41,513.6	0.11	Loss	*De novo*	3	*CASK* (Exonic)	Confirmed: X-linked MR (OMIM:300749)
MR_452 (Male)	5Y	Delay in motor development, epileptic seizures, facial dysmorphic features	Xp21.1	31,674.3- 31,762.8	0.09	Loss	Inherited from mother	1	*DMD* (Exonic)	Confirmed: Duchenne muscular dystrophy (OMIM:310200); incidental finding
MR_410iii (Male)	4Y5M	Global DD, macrocephaly, hypertonia; Eccentric and repetitive behavior, restricted interest, poor social communication.	9q33.1	118,395.0–118,716.1	0.32	Loss	Inherited from mother	2	*TRIM32*, *ASTN2*	Confirmed: risk locus for autism and other neuro-developmental disorders^89^
MR_3699 (Male)	5Y	Microcephaly, speech problems, spastic movement	2p16.3	50,990.3–51,066.3	0.08	Loss	Inherited from mother	1	*NRXN1 (Exonic)*	Confirmed: risk locus a range of developmental disorders^90^
MR_1194^iv^ (Male)	Prenatal	Congenital absence of abdominal muscle, died within first month after birth	1q21.1	145,037.9–145,537.2	0.50	Gain	Inherited from mother	3	*CHD1L*, *PRKAB2*	Probable: critical gene of 1q21.1 deletion/duplication syndrome^27^,^28^
MR_1234 (Male)	11M	Developmental delay, mild facial dysmorphic features.	19p13.3	4,973.6–5,099.4	0.13	Loss	*De novo*	1	*KDM4B*	Probable: *De novo* loss of function variants associated with autism^46^
MR_496 (Male)	1Y5M	Hyperactivities, feeding difficulty, sleeping problem, deficits in emotional behavior	7q31.33	125,762.5–125,899.9	0.14	Loss	Inherited from mother	1	*GRM8* (Exonic)	Probable: Deletions associated with ADHD^91^ and reported in autism^92^
MR_3861^v^ (Male)	2Y	Intellectual disability, language impairment, gross motor delay, facial dysmorphic features (flat nose, long philtrum), single palmar creases	8p23.3	0–2,015.5	2.2	Loss	*De novo*	9	*DLGAP2*	Likely: *De novo* deletions identified in autism^93^
MR_1117 (Male)	7Y	Hyperactivity, short attention span, learning difficulties (speech delay, poor comprehension, memory weakness), facial dysmorphic features (long face, protruding eyes)	8p21.3	22,169.5–22,817	0.65	Gain	*De novo*	10	*PPP3CC*	Likely: Reported duplication carriers with mood disorder^47^ and schizophrenia^48^
MR_3465 (Male)	3Y10M	Gross motor delay since birth, intellectual disability, no limb abnormality	7q21.3	95,097.5–95,870.5	0.77	Loss	Inherited from mother	2	*SLC25A3*, *DYNC1I1*	Likely: SHFM locus (OMIM:183600); reported deletion carriers with MR without SHFM^69^

CNVs of known genomic disorders are not shown in this table. ADHD: attention deficit hyperactivity disorder, SHFM: split-hand/foot malformation.

^i^Age at the time of sample DNA collection. Clinical features may also include the findings from patient follow-ups.

^ii^*De novo* occurrence in this case is presumed based on fully penetrant phenotype.

^iii^Patient MR_410 also carried a second large pathogenic deletion at 22q13 (*SHANK3*).

^iv^Mothers of the patient MR_1194 reported stillbirth or miscarriages in her previous three pregnancies; fetuses also showed abdominal wall defects.

^v^Patient MR_3861 also carried a second large pathogenic duplication 2q35-q37 (26.7 Mb, 208 genes). Both the duplication and deletion extend to telomeres, likely caused by a single unbalanced translocation. This case was a false negative in subtelomeric aberration screen.

**Table 4:** Potential pathogenic CNVs identified in parent that are not found in patients.

Sample	Cytoband	Start - End (kb)	Size (Mb)	Copy number	Num of Genes	Candidate Genes	Pathogenicity
CTRL_1848 (Female)	16p13.11	15,147.1–18,063.9	2.92	Loss	10	*ABCC6*, *MYH11*, *C16orf45*, *XYLT1*, *ABCC1*, *KIAA0430*, *NDE1*	Confirmed: 16p13.11 deletion syndrome^94^,^95^
CTRL_2233 (Female)	14q22.2	53,476.5–53,502.9	0.03	Loss	1	*BMP4*	Confirmed: loss of function mutations causes abnormalities in eye and brain^96^,^97^,^98^
CTRL_2580 (Female)^i^	2q36.1-q36.3	224,278.6–227,232.0	2.95	Loss	8	*SERPINE2*, *CUL3*, *WDFY1*, *DOCK10*, *MRPL44*, *NYAP2*	Probable: Loss of function variants in *CUL3* is associated with autism^46^ and congenital heart defects^99^.
CTRL_2060 (Female)	4p16.3	1,950.6- 2,016.5	0.07	Loss	2	*WHSC1*(3’UTR), *WHSC2*	Probable: Wolf-Hirschhorn syndrome critical region^100^.
CTRL_1964 (Female)	3p26.2-p26.3	754.2-4,668.1	3.91	Gain	9	*CNTN4*, *CNTN6*, *ITPR1*, *TRNT1*, *LRRN1*, *CRBN*	Likely: Reported duplication carriers with neuro-developmental and psychiatric disorders^101^,^102^
CTRL_3796 (Female)	9p24.3	597.7-753.0	0.16	Loss	1	*KANK1* (Exonic)	Likely: Associated with neurodevelopmental diseases^103^,^104^

^i^The DNA of her son failed QC and was not tested. In all other cases, the potential pathogenic CNV was confirmed un-transmitted.
