# Supplementary material for: CNV analysis in Chinese children of mental retardation highlights a sex differentiation in parental contribution to de novo and inherited mutational burdens
Source: Sci Rep. 2016 Jun 3;6:25954. doi: 10.1038/srep25954 (PMC4891738; doi:10.1038/srep25954)
Supplement: Supplementary Information [file srep25954-s1.pdf]

Supplementary Information

CNV analysis in Chinese children of mental retardation highlights a sex differentiation in parental contribution to *de novo* and inherited mutational burdens

Binbin Wang[*], Taoyun Ji[*], Xueya Zhou[*#], Jing Wang[*], Xi Wang, Jingmin Wang, Dingliang Zhu, Xuejun Zhang, Pak Chung Sham, Xuegong Zhang, Xu Ma[#], and Yuwu Jiang[#]

Supplementary Methods

SNP Quality Control (QC)

Raw signals of Illumina bead array (Illumina CytoSNP12, ~300k markers) were first pre-processed by GenomeStudio® and had genotypes called using default genotype cluster files. We excluded samples with less than 90% call rate, and inferred genders for the remaining samples based on signal intensities on chromosome X. SNP genotypes were then re-clustered using the remaining samples. On chromosome X, only females were used to generate clusters; the new cluster was then used to genotype males. After reclustering, samples with less than 95% call rate were excluded.

We used PLINK software (Purcell et al. 2007) for SNP genotype processing and management. Duplicated samples were detected if their genotypes shared over 99% identity-by-states (IBS); only the one with highest SNP call rate was kept. The family relationships were checked using identity-by-descent (IBD) estimates from genome-wide SNP markers. Parents-offspring pairs were expected to have Pr(IBD=1)>0.9 and estimated kinship coefficient 0.4~0.6. Parental samples that did not fit with the claimed relationship were removed from the trios. No cryptic relatedness up to third degree of relatives (kinship coefficient > 0.075) was found among patients. Genders were verified using the estimated inbreeding coefficient on chromosome X. Samples with highest genome-wide heterozygosity rate were manually inspected on their array signals to exclude the possibility of cross-sample contamination. After sample-level QC, 289 MR/DD patients (191 males, 98 females) and 510 parents were retained in genotype analysis, including 218 trios, 52 pairs and 19 singletons (Table S1A).

For inclusion of SNPs in genotype analysis, we required marker should pass the following QC criteria: (1) had genotype calls in >= 97% individuals (>=99% individuals if minor allele frequency (MAF) < 0.05); (2) exhibited Hardy-Weinberg equilibrium in unrelated samples with p>1E-5; (3) had Mendelian transmission errors in less than 5% of the trios. The genetic position for each QC-passed SNP was interpolated from fine-scale recombination rates (Myers et al. 2005). A total of 277,842 high quality SNPs on CytoSNP12 array passed marker-level QC.

SNP Genotype Analysis

We assessed the population ancestry using principle component analysis (PCA) (Patterson, Price, and Reich 2006). To combine with HapMap samples, QC passed SNP markers that

can be aligned to HapMap Phase 3 panel (Release 2) were retained, and thinned to be in approximate linkage equilibrium using PLINK option "--independent-pairwise 100 25 0.25". MHC regions were excluded to prevent the confounding effects of long range LD. Only unrelated patients were used in PCA.

When combined with three major continental populations of HapMap (CEU, YRI, and CHB+JPT), we found two outliers in the first two principle component axes: one was an admixture of European and Asian like Uyghur, the other might have African ancestry (Figure S2A). After removing two outliers. The patient cohort mixed well with Chinese population (CHB) but separated from Japanese (JPT) (Figure S2B).

Constituent uniparental disomy (UPD) in MR/DD children was detected as chromosome outlier in Mendel errors. We first tabulated the number of Mendel errors on each chromosome for each trio, and estimated the expected proportion of errors for each chromosome using trimmed mean across trios. The unusually large number of errors on one particular chromosome for each patient was then evaluated by exact binomial test. P-values were corrected for 218×23 tests. We also screened for the runs of homozygosity (ROH) using plink's "--homozyg" option with default parameter. The total genetic and physical length of ROHs were summed up for each individual. UPD and ROH analyses were done after zeroing out individual genotypes within CNVs.

CNV Calling

LogR ratios (LRRs) and B-allele frequencies (BAF) signals of all subjects passed SNP QC were exported from GenomeStudio®. We applied PennCNV to calculate quality control statistics. Obviously bad samples were spotted as outliers in the distribution and removed from CNV calling. These included samples that had LRR standard deviation>0.35, BAF median>0.55 or <0.45, BAF drift >0.02, or |waviness factor|>0.15. CNVs were called using PennCNV package (Wang et al. 2007) based on the standard hidden Markov model for Illumina arrays, and customized population B allele frequencies calculated from unrelated samples included in CNV calling. The GC content-based wave adjustment was applied (Diskin et al. 2008). We only kept CNV calls spanning at least 5 markers and having confidence score >15.

The raw CNV calls were removed if they were mapped to the following artifact regions: 500kb within start or end of chromosome, within centromeres, and immunoglobulin loci (Need et al. 2009). To determine an appropriate quality control procedure, we first manually inspected the LRR/BAF signals of all large CNV calls (>800kb) and all smaller CNVs that were absent in population controls and appeared only once in each pedigree (private CNVs). We observed that although smaller CNVs in noisy samples were fraught with false positives, large CNVs could still be reliably distinguished. To identify potential samples with poor CNV calling performance, we also calculated two other QC measures adopted by Sanders et al. (2011): (1) Excessively wide LRR band, defined as the number of probes with |LRR|>0.5; (2) The number of aberrant behaving probes, defined by highly negative (extreme) LRR values (LRR<-1.0). The relationship between the total number CNVs calls and different QC metrics are shown in Figure S3. Noisy samples were empirically defined as those had LRR standard deviation>0.3, |waviness factor|>0.10, number of markers with wide or extreme LRR values fall above 90% quantile across all

samples. CNV calls <500kb in noisy samples were excluded from subsequent analysis. We also noted that PennCNV tended to generate smaller fragmented calls for a large CNV, possibly due to uneven probe coverage on the array. We therefore iteratively merged neighboring CNV calls of the same copy number, if the sum of original lengths was at least 70% of the merged CNV length. The excessive number of CNV calls on a particular chromosome may also be indicative of chromosome aneuploidy. Before CNV merging, we estimated the proportion of CNV calls made on each chromosome across the sample, and detected chromosomes with significantly more than expected CNV calls using a goodness-of-fit test. One mother with chromosome X trisomy was found and CNVs on that chromosome were not included for subsequent analysis.

CNV Filtering and QC

To enrich for pathogenic CNVs, we applied a series of filters to the cleaned CNV calls. We first excluded CNVs that have 50% of its region overlapping with a known copy number polymorphism (CNP) reported in HapMap samples (McCarroll et al. 2008, Bailey, Kidd, and Eichler 2008, Conrad et al. 2010, Park et al. 2010, International HapMap et al. 2010). Then we excluded CNVs whose 50% region overlap with CNV segments of the same type in at least 5 unrelated subjects from population-based controls, or at least 2 unrelated parents. The number of CNV calls after each step of filtering is shown in Table S2. After automated filtering, we found 345 CNVs in 185(64.5%) patients and 612 CNVs in 280(54.9%) their parents. The inheritance status of CNVs found in patients was initially determined by comparing to the unfiltered CNV segments in parents. All the resulting CNVs were subject to manual inspection to check the carrier status in related samples, split incorrectly merged nearby CNVs, resolve incorrect boundaries, and flag the ambiguous cases for validation.

We initially selected 64 representative CNVs from the curated set for quantitative real-time polymerase chain reaction (qRT-PCR) validation. These include 16 CNVs with size 250~600kb that overlap candidate genes, and 48 small CNVs <250kb (22 duplications, 26 deletions) that overlap at least one gene. For CNVs in patients, validations were also performed in parents to confirm inheritance status. To evaluate the CNV calls in noisy samples, we also applied the above filtering procedures on all samples, and randomly select 20 ultra-rare CNVs overlapping at least one gene in noisy samples for validation. All 16 CNVs >250kb in cleaned samples were validated, as compared with only 2 out of 20 CNVs in noisy sample (Figure S4B). It demonstrated the accuracy of manual curation on large CNVs on clean samples, which will be used in CNV burden analysis. Given the low validate rate, no further validation was attempted on noisy samples. Validation rate was ~62% for small CNVs in cleaned sample. To derive an operational filter, we compared the LRR of each CNV to the markers in the neighboring regions at the right and left sides. The neighboring region was selected to have similar size as the CNV with a minimum of 10 markers. We defined $\Delta LRR_{right(left)}$ as the difference in mean LRR of markers in the CNV segment and the marker in the right (left) neighboring region. If not enough markers were found within 1Mb surrounding region of the CNV or there were CNVs nearby, then ΔLRR was set to the mean LRR of markers in the CNV. We empirically determined the QC filter ΔLRR >= 0.15 for duplications and <= -0.25 for deletions, which achieved >90% sensitivity and >85% specificity on CNVs selected for validation (Figure S4A). Most false positives were caused by the markers with extreme LRR in deletion calls, and could be

identified by manual inspection. When applied the filter to the CNV calls, we observed an increase in concordance rate of CNV calls in duplicated samples without much loss of sensitivity (Table S3).

The QC filter was then applied to the ultra-rare CNVs <250kb passed automated filtering, with additional 51 ambiguous cases resolved by further validation (33 of them are validated and included in the final set). The QC of ultra-rare CNVs >=250kb were only subject to manual curation. We performed additional qRT-PCR experiments to validate all pathogenic CNVs <600kb reported in main text and Table S10. Seven *de novo* CNVs <600kb after curation were also validated to be absent from parents. The final set of ultra-rare CNVs in patients and parents are given in Table S8 and Table S9. The numbers of CNVs after validation, QC, and curation step are shown in Table S2. The iterative process of validation, QC and curation not only reduced the likely false positives; they also reduced the false negatives as compared with automated filtering.

To assess the frequency of CNVs in non-Chinese populations, we also obtained published CNV calls obtained from three population controls: WashU (Itsara et al. 2009), CHOP (Shaikh et al. 2009), and OPGP (Uddin et al. 2014), totaling up to 5000 samples of different ancestries (Table S1C). Among ultra-rare CNVs identified in our clinical cohort, none of them had 50% of its region covered by >=5 CNVs of them type.

Control Cohorts Analysis

For population-based controls, genotypes were called by GenomeStudio® using the default genotype cluster file. Samples with less than 98% call rate were excluded. Duplicated samples and related samples up to 2nd degree relatives (kinship coefficient >0.1875) were identified; and only one with highest SNP call rate was kept. Sample genders were verified based on chromosome X inbreeding coefficient. A total of 6,268 samples passed SNP genotype QC (Table S1B). The Chinese ancestry was verified by PCA (not shown).

All subjects that passed SNP QC procedures were entered into CNV calling. The CNV calls were generated using the same method (PennCNV) as the clinical cohort, with customized population B allele frequencies and wave adjustment. To reduce the labor for manual curation, only CNVs found in samples passed stringent QC criteria were retained. The QC step excluded samples that had LRR standard deviation>0.275, BAF drift>0.002, WF>0.06 or WF<-0.07. Then we further removed samples having >100 total number of CNV calls. A total of 5866 control samples passed QC, including 2780 males, 3086 females (Table S1B). We only kept CNVs that were supported by at least 5 probes and having PennCNV confidence score>10. The CNV calls located within genomic regions of known artifacts were excluded. Two chromosome X trisomy females were identified; CNVs on these two chromosomes were not included for further analysis. Neighboring CNVs of the same type were merged if the sum of original lengths were at least 80% of the merged length.

Targeted Rare CNV Genotyping

We adopted SNP-Conditional OUTlier detection (SCOUT) algorithm (Mefford et al. 2009) implemented in SCIMMkit package (Zerr et al. 2010) for targeted rare CNV genotyping in our clinical cohort. Only 22 autosomes were analyzed.

To select probes informative for CNV genotyping, we followed the recommendation of the software developer. After QC on SNP genotypes, the following more stringent criteria were used to select SNP probes: MAF>0.1, missing call rate<0.03, and HWE test p-value>10E-4. The resulting 186,996 autosomal probes were selected.

To define targeted regions, we first compiled a list of 2928 known and candidate genes for MR/DD. Genomic regions spanned by 5 consecutive markers and less than 100kb were enumerated. If a region overlapped coding exons of the candidate gene and was not contained within deletions or duplications found in >=5 population-based controls, then it was nominated as a target region. A total of 14,139 target regions were defined.

We noted that inclusion of noisy samples would reduce the sensitivity of this approach, because SCOUT algorithm uses the probe signal intensities across the samples to detect outliers. So the samples passed QC for small CNV calling were further filtered to exclude those whose wide and extreme LRR markers (see CNV Calling, Filtering, and QC section) were above 85% quantile across all samples. This resulted in 233 patients and 410 parents left for SCOUT analysis (Table S1A).

The genotyping was performed using the default parameters adopted by SCIMMkit; CNV calls with absolute per-site score >6 were generated. The overlapping CNV segments of the same type were subsequently merged. We tested the sensitivities of rare CNV genotyping on the curated ultra-rare CNVs. Among CNVs in the final list (Table S8, Table S9), we found 68 CNVs (31 duplications, 37 deletions) that cover the targeted regions and were carried by samples passed QC for SCOUT analysis. SCOUT detected 58 of them (27 duplications, 31 deletions) achieving an 85.3% recovery rate. Further increase the score threshold to 6.5 and 7.0 would reduce the recovery rate to 80.9% and 69.8% respectively.

To discover additional gene disrupting CNVs, the resulting CNV calls were filtered to exclude those that were already identified by PennCNV or also called in at least 2 unrelated parents. Then, further sample level QCs were performed to exclude those harboring more than 4 rare CNV calls after filtering, as manually inspecting the signal plots suggest that they were most likely noisy samples. This procedure resulted in 57 candidate CNVs (38 duplications and 19 deletions) in 37 samples. None of the duplications encompassed an entire gene transcript. As the functional consequence of duplications that partially overlap the coding sequence of a gene was hard to interpret, we focused on deletions (details given in Table S14). QRT-PCT validation was attempted at coding exons encompassed by 11 CNVs. We validated one deletion in the exon 1 of *HIRA* of MR_3590, which was not inherited from father (Figure S10). High rate of false positives was also suggested by a large number of apparent *de novo* calls. Manually inspecting the signal plots suggested two sources failed validations: aberrant behaving probes in noisy samples, or the real deletion was smaller than the region spanned by five probes. In two cases where CNV calls were also made on mothers (*BMP6* and *GPC6*), validations targeting at the intronic region defined by the probes with strongest signals were successful. Together, we identified three additional small CNVs using the targeted genotyping approach.

Mosacism Calling

To identify mosaic structural changes, we first applied MAD package (Gonzalez et al. 2011) to detect consistent skews in mirrored B-allele frequency signals. The following parameters

were used for segmentation: aAlpha=0.8, T=9, MinSegLen =100. A total of 70 candidate segments of mosaic events were called. We first excluded segments that overlapped CNVs called by PennCNVs, and those that overlapped constituent ROHs identified by PLINK. The resulting segments were then subject to manual inspection to exclude remaining error classifications caused by noisy or waviness signals, artifacts regions on chromosome X, etc.

Supplementary Figures

Figure S1 The computational workflow for CNV filtering and interpretation: an overview.

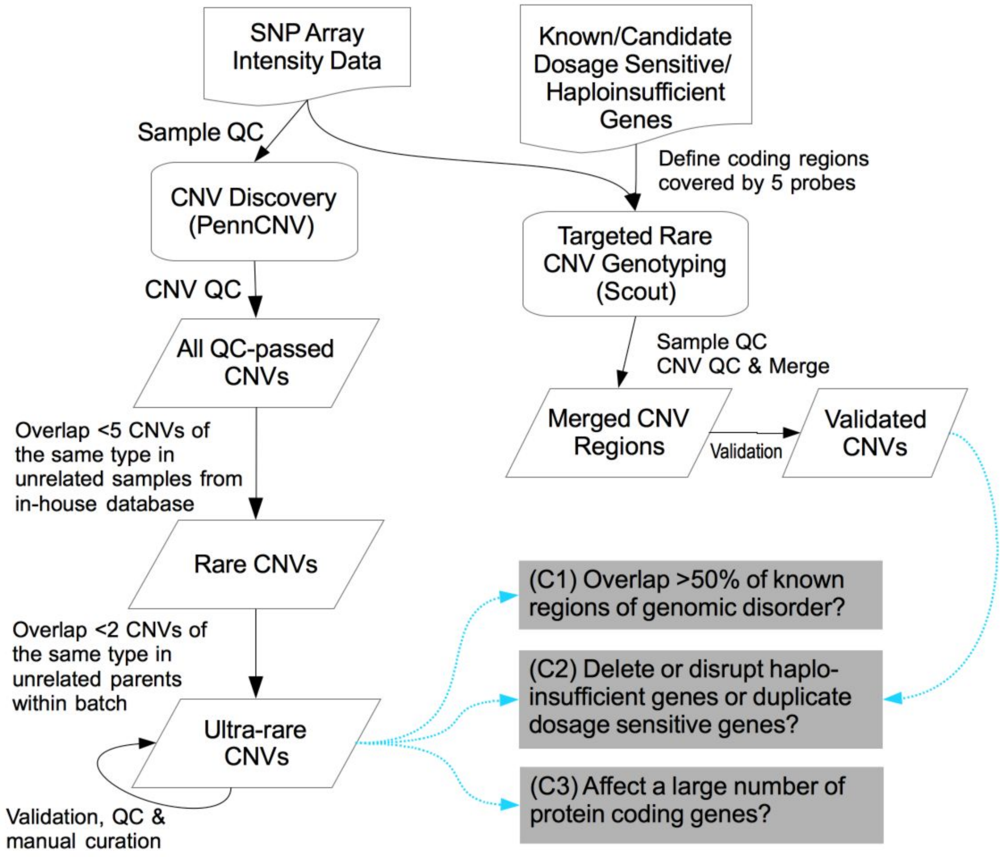

After SNP genotype-based QC, we performed further QC on samples based on array intensities before CNV calling. High confidence CNV calls were subject to a series of filters to enrich causal variants. The steps included the exclusion of known CNPs, CNVs of the same type that occurred at least five times in population controls, and CNVs detected in at least two other unrelated parents. The resulting ultra-rare CNV calls were subject to iterative QC, manual curation, and experimental validation to generate the final CNV list. To evaluate the pathogenicity, we first check if a CNV overlaps the critical region of known genomic disorders (GD) matched for the copy numbers (C1). Then, we check if the CNV deletes or disrupts the coding exon of haplo-insufficient genes or duplicate triplo-sensitive genes (C2). If no overlap with such region or gene was found, we subjectively judged the CNV as pathogenic if it affects a large number of genes (C3). To opportunistically identify smaller exonic CNVs that might be missed by standard calling algorithm, we also started with a list of known/candidate disease genes, and defined target regions that cover the coding exons and contain enough informative SNP probes. Then, targeted rare CNV genotyping were performed to search for additional small CNVs. The results were subject to validation; and their pathogenicity was assessed based on C2.

Figure S2 Evaluating the population ancestry and structure of MR/DD patients using principle component analysis. (A) The cluster plot of first two principle components (PCs) when combing patients with four HapMap populations; two outliers are indicated by grey arrows. (B) After removing outliers, the cluster plot of first two PCs combined with two East Asian populations.

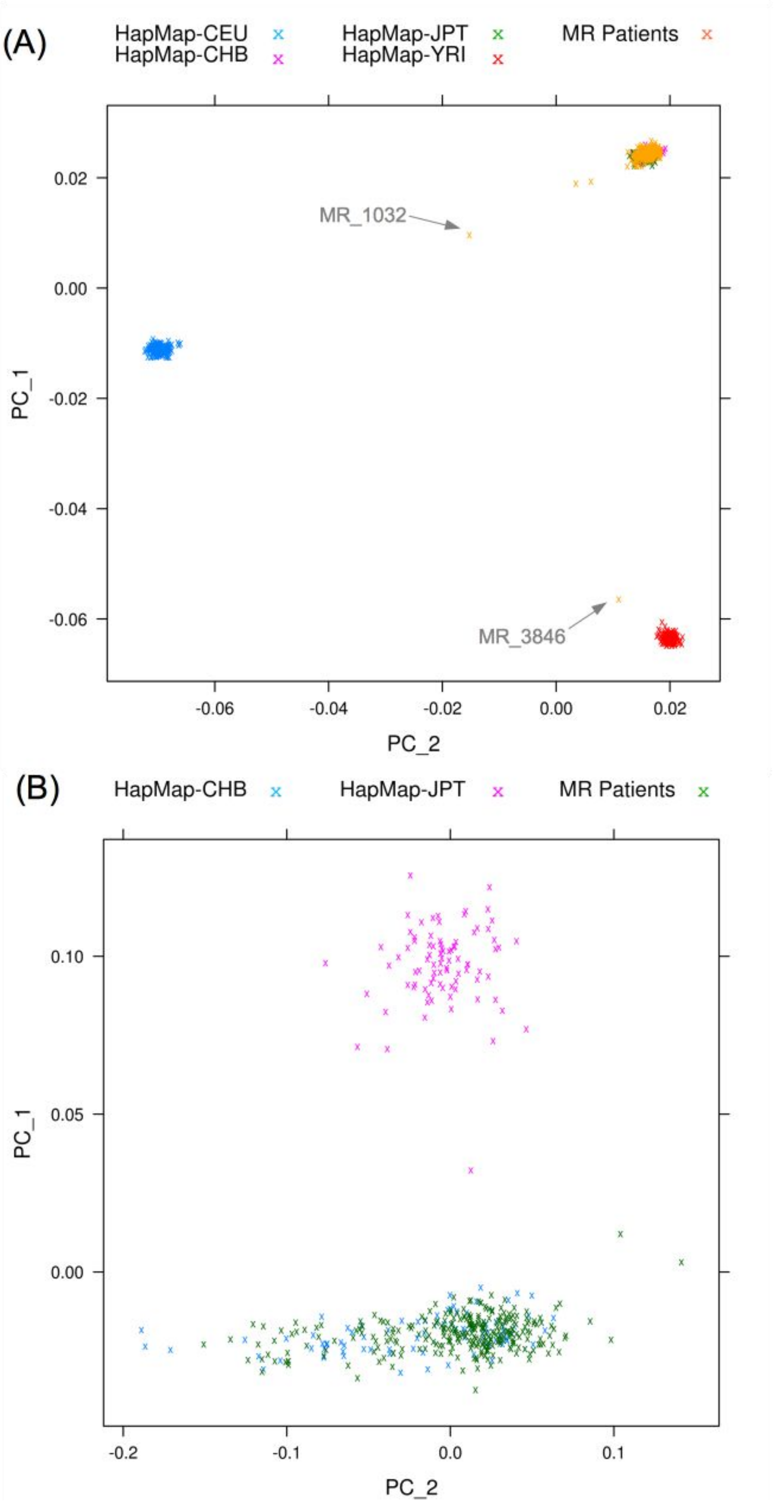

Figure S3 Factors influencing the total number of CNV calls.

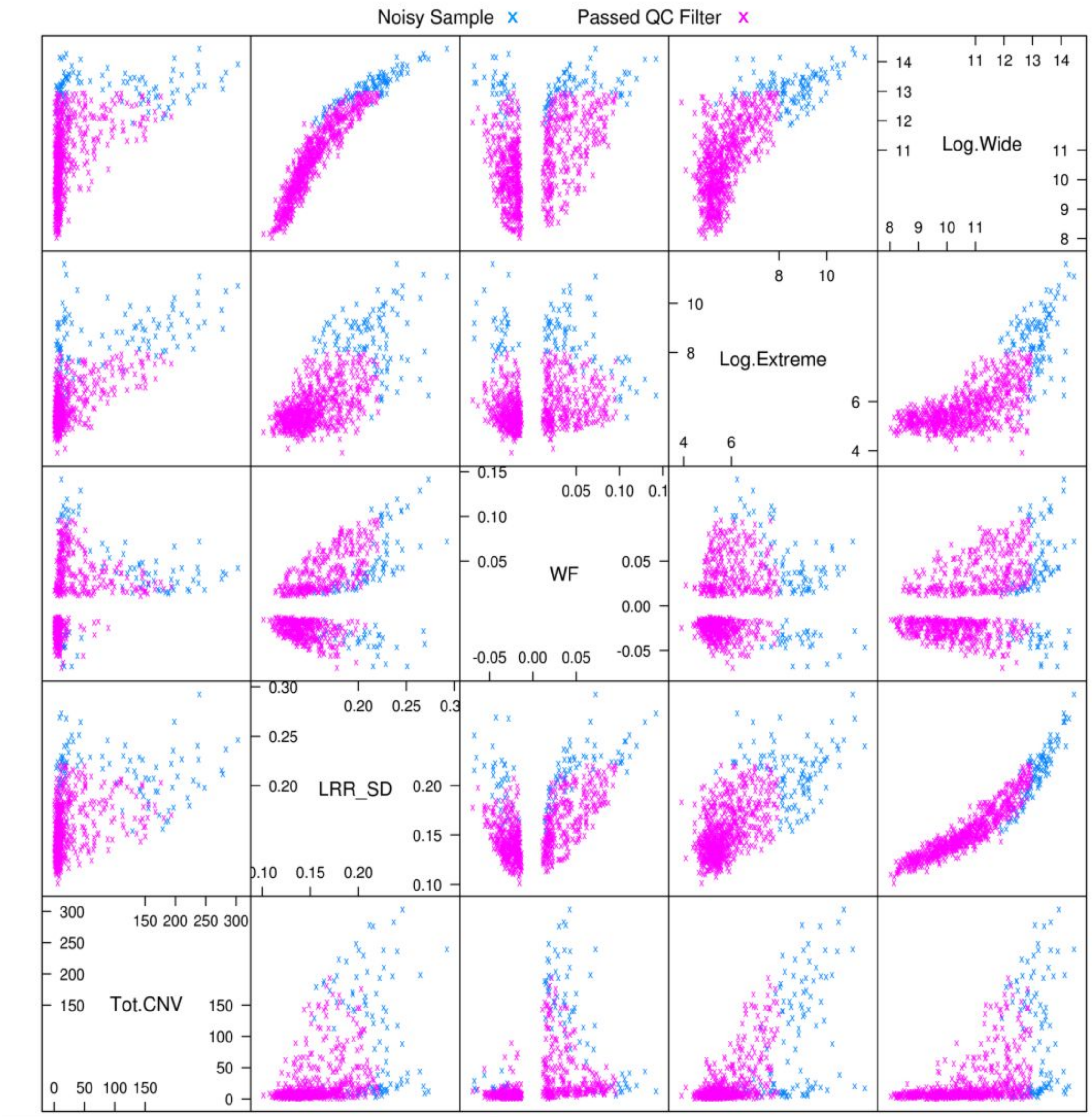

The scatterplot shows the relationships between different sample-level QC metrics and total number of CNV calls. LRR_SD: standard deviation of log-R ratio (LRR) signals; WF: waviness factor, a summary measure of the LRR signal fluctuation explained by local GC content (Diskin et al. 2008); Log.Extreme: log2 of the number of probes with LRR less than -1.0; Log.Wide: log2 of the number of probes whose LRR deviate at least 0.5 from 0. Different QC metrics are correlated and capture different aspects of data quality. Noisy samples (defined in CNV Calling, Filtering, and QC section) are shown in blue. They generally have higher number of total CNV calls suggesting higher false positives.

Figure S4 The validation of small CNVs (<250kb) in (A) clean and (B) noisy samples.

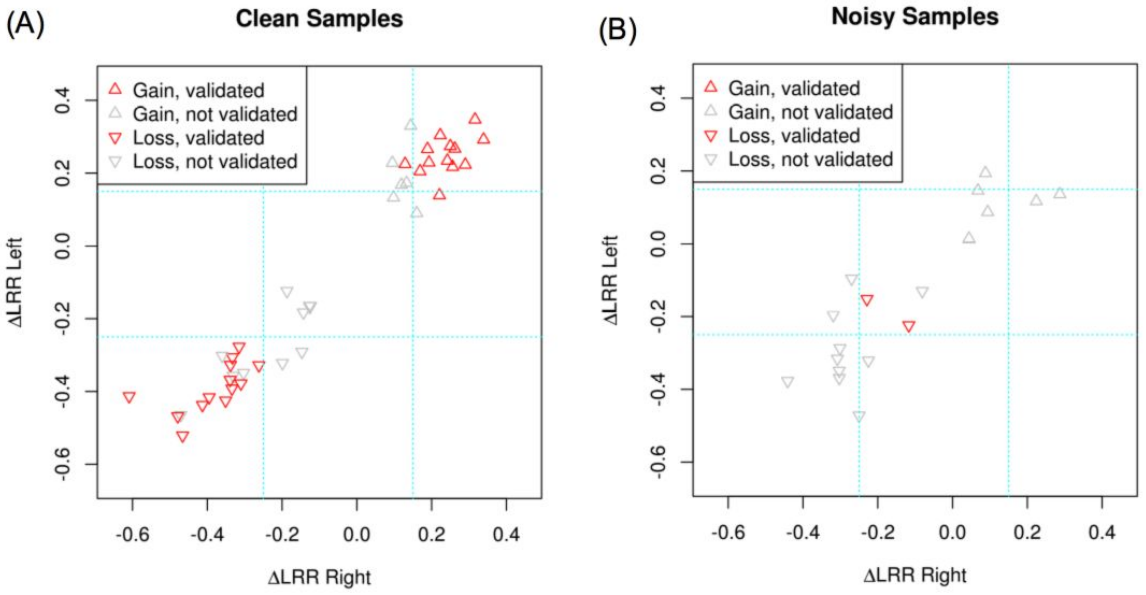

For each CNV, ΔLRR Left (Right) is defined as the difference in mean LRR of markers in the CNV segments and the markers in the left (right) neighboring region of similar size as the CNV with a minimal of 10 markers. The thresholds of 0.15 and -0.25 are shown in cyan lines.

Figure S5 The logR-ratio/B-allele frequency signal plots for all pathogenic or likely pathogenic CNVs.

Supplementary Figure S5

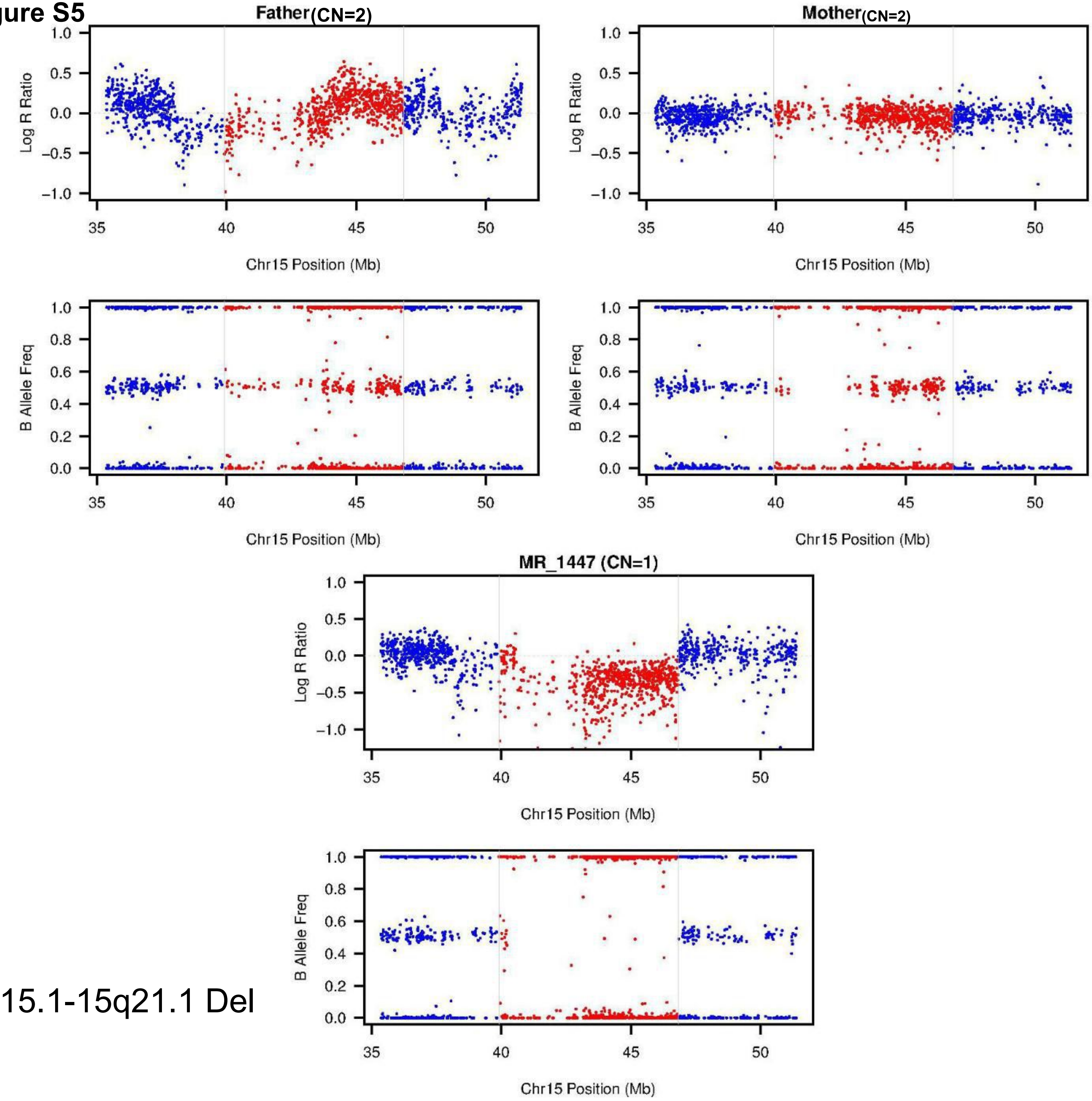

MR_1447: 15q15.1-15q21.1 Del

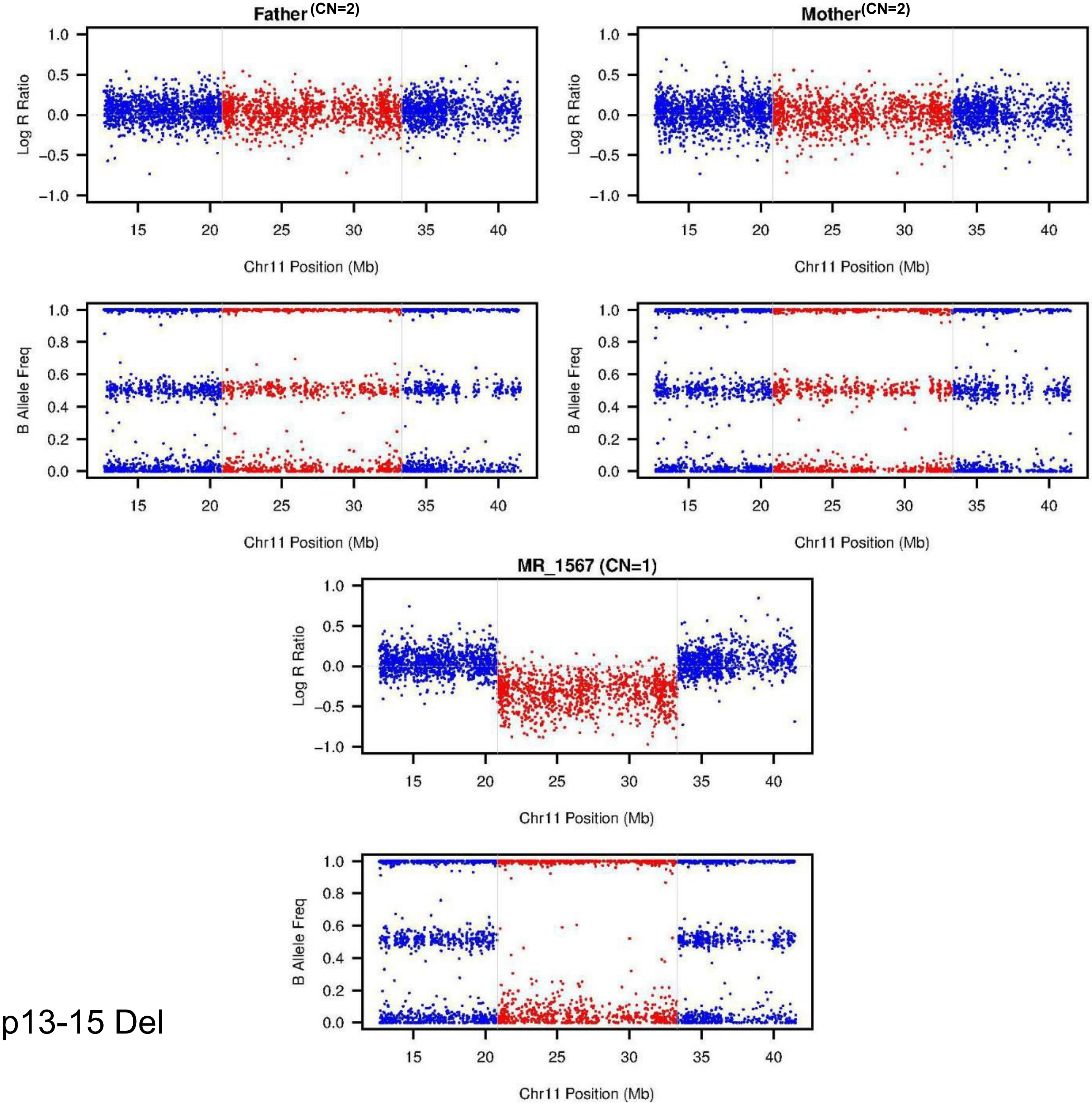

MR_1567: 11p13-15 Del

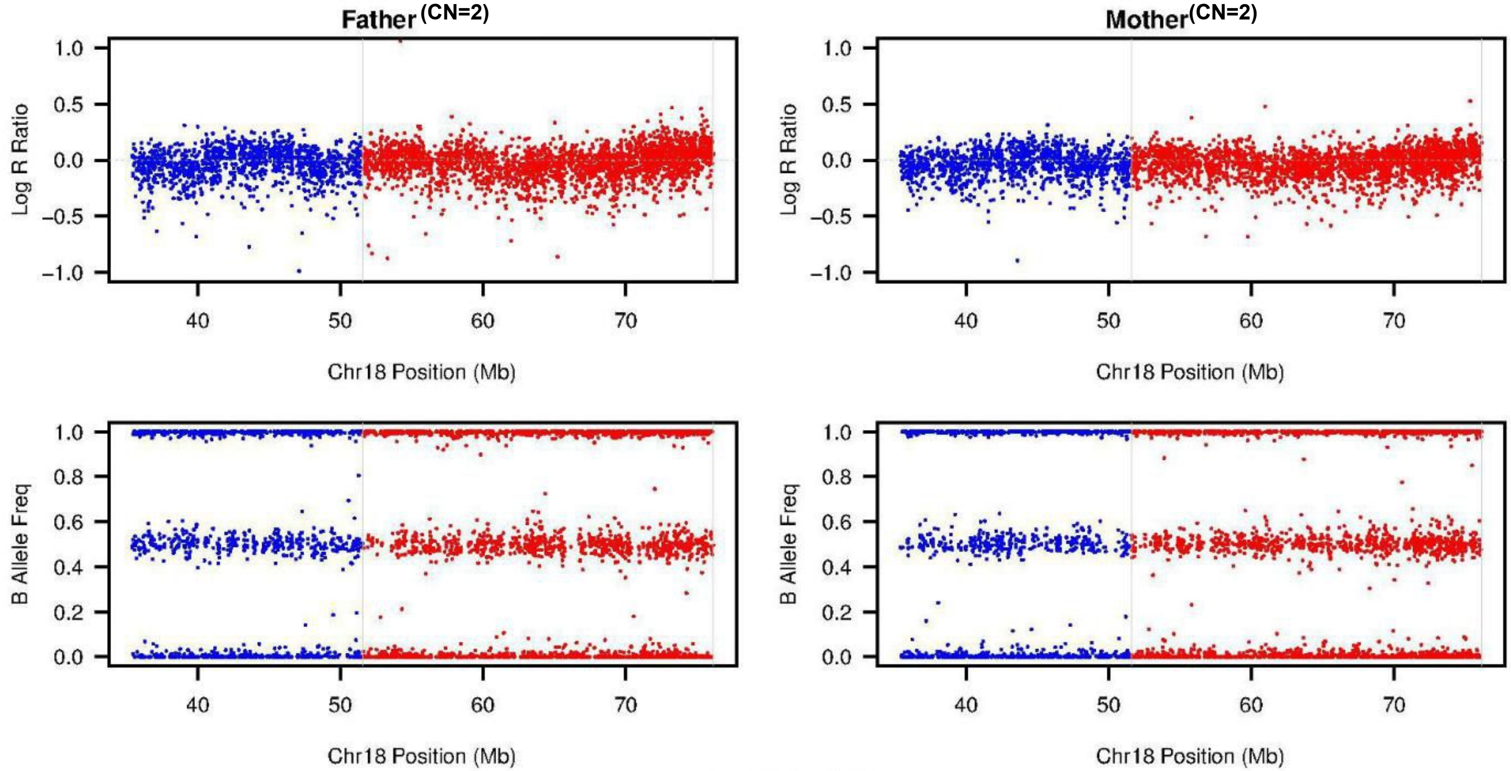

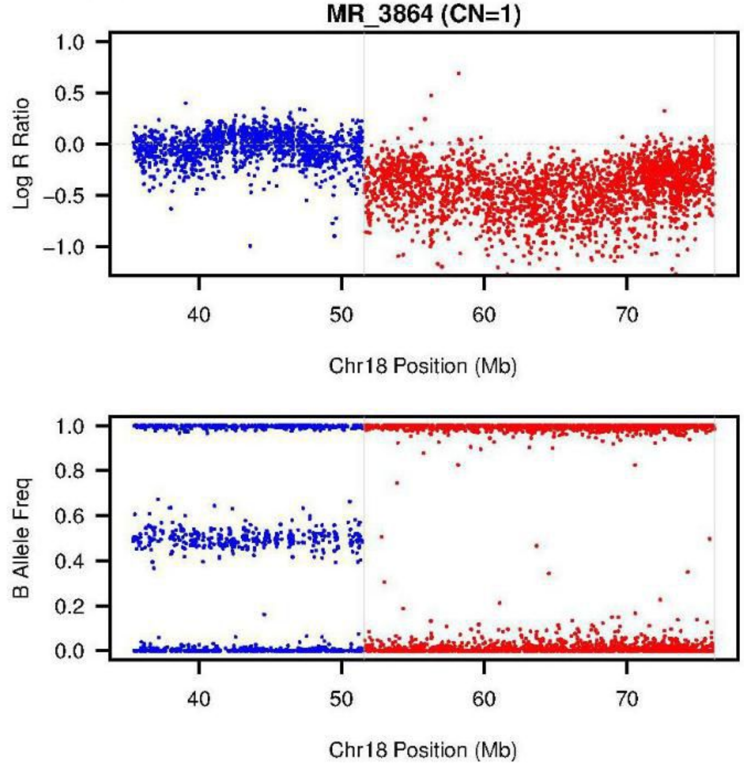

MR_3864: 18q21-18qter Del

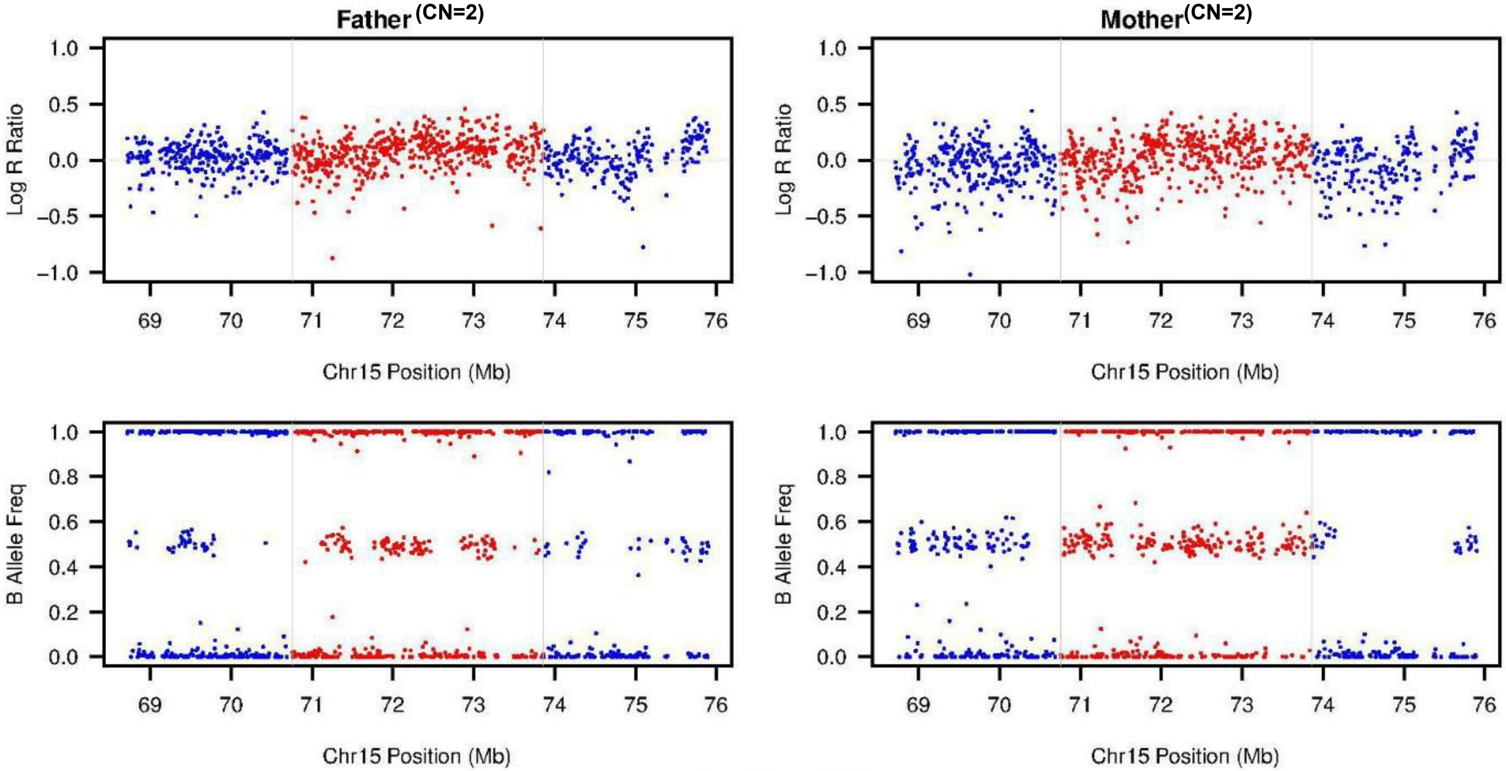

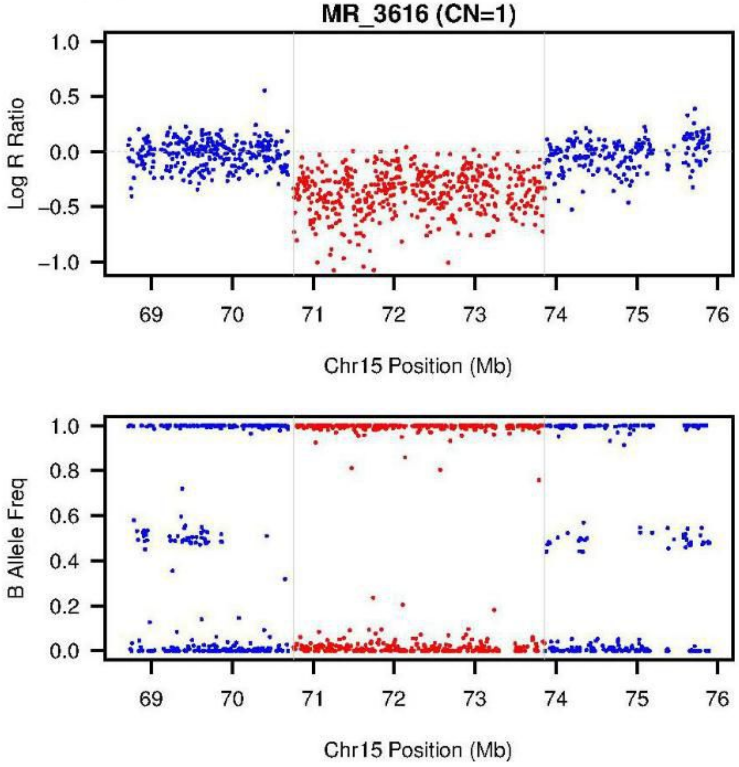

MR_3616: 15q24 Del

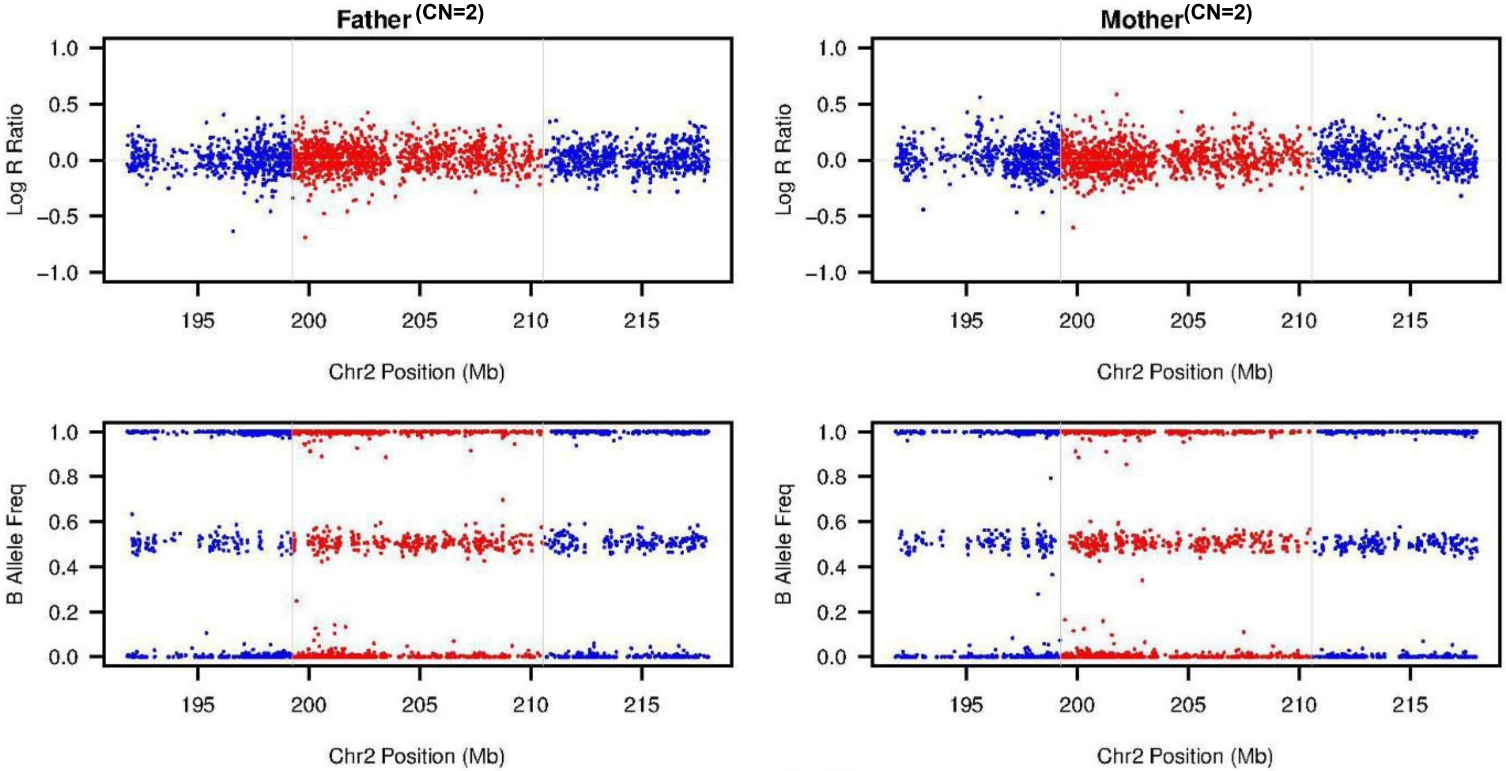

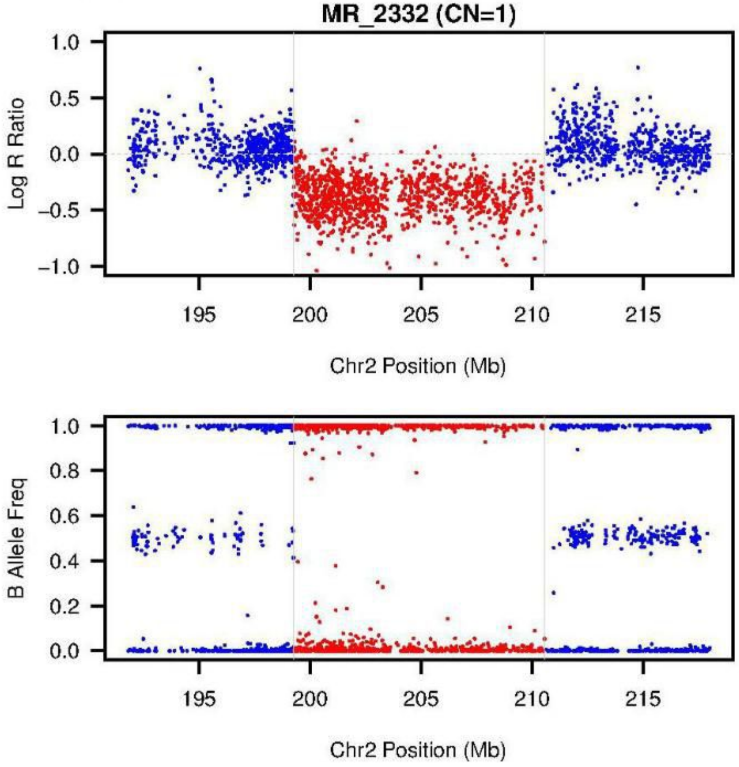

MR_2332: 2q32-2q33 Del

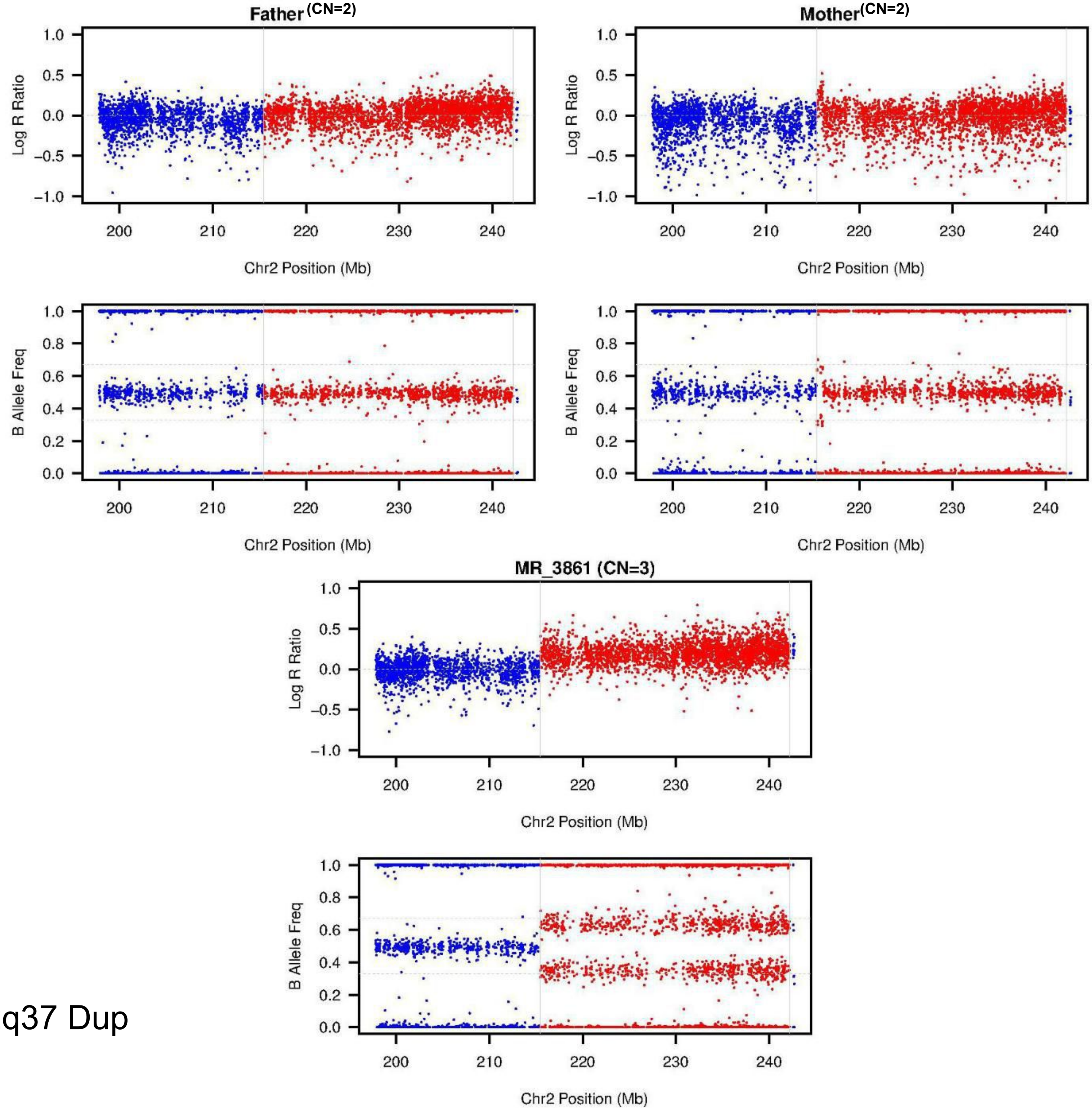

MR_3861: 2q37 Dup

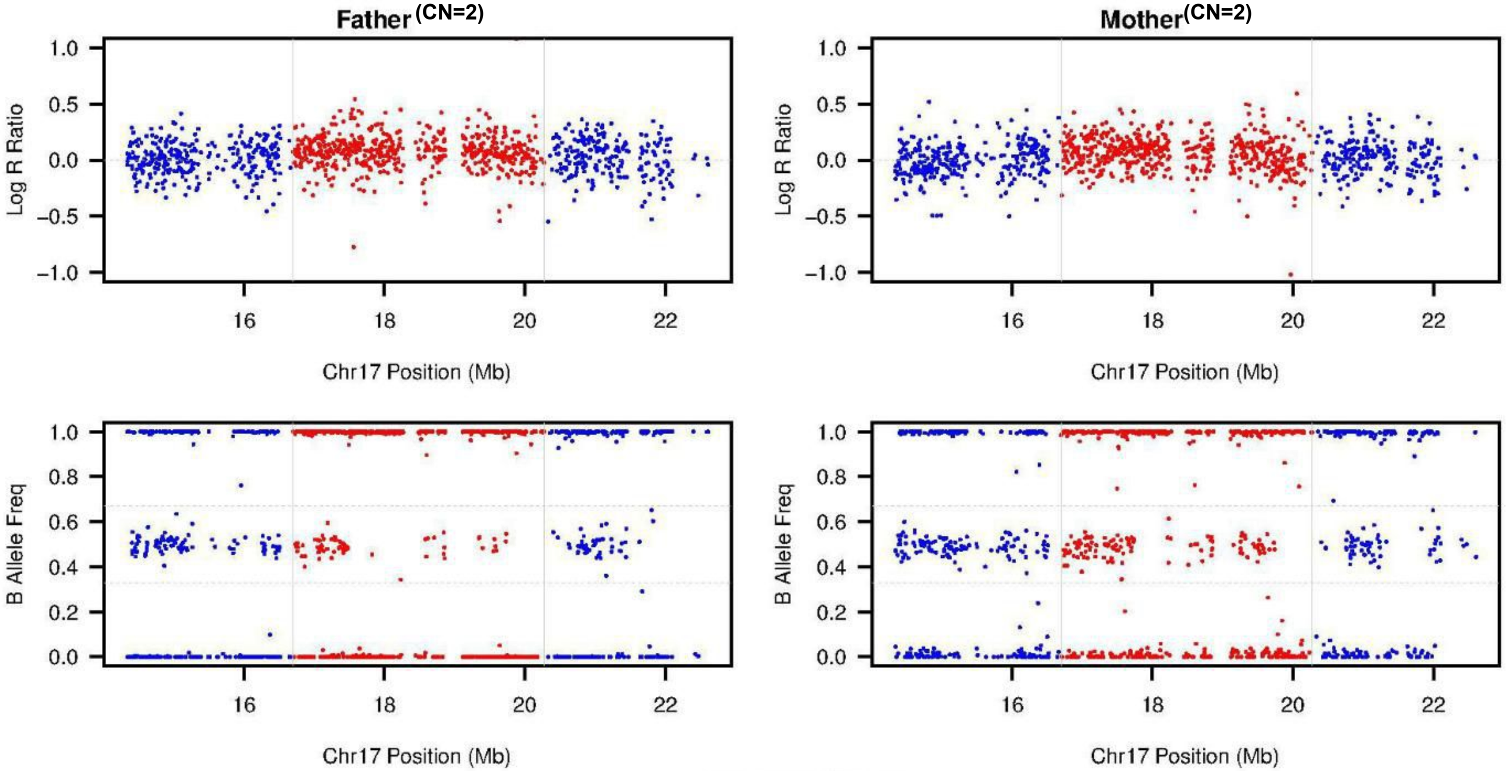

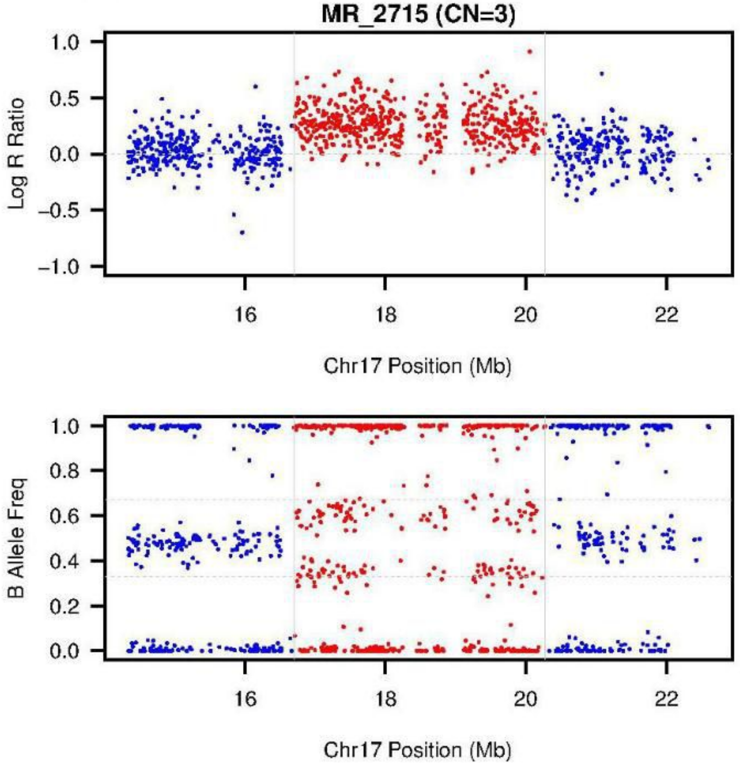

MR_2715: 17p11.2 (SMS) Dup

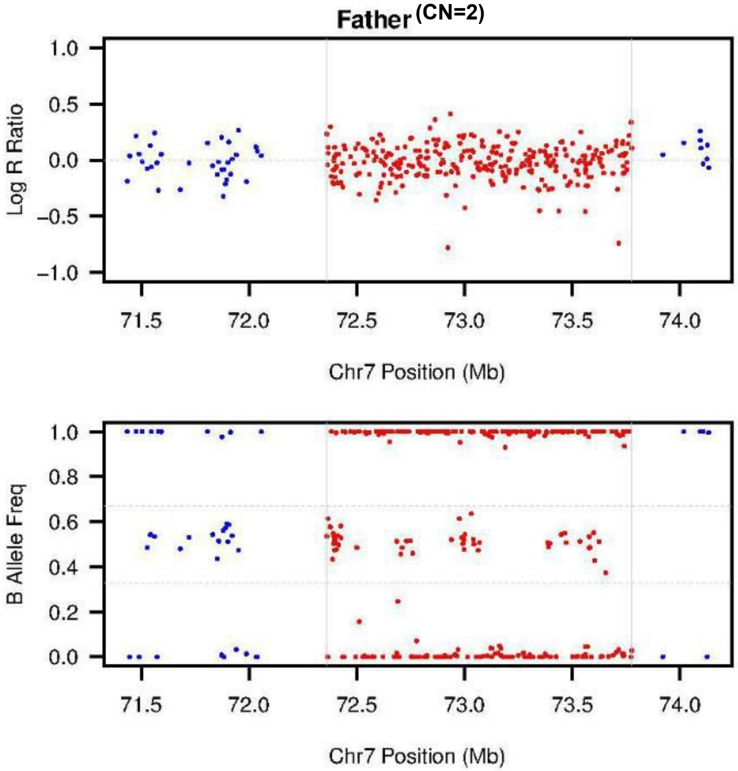

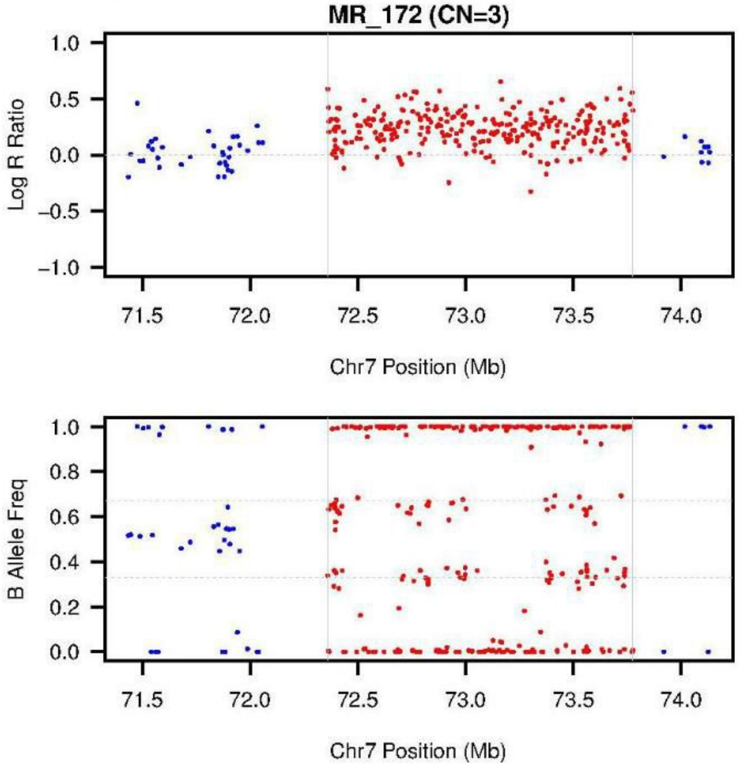

MR_172: 7q11.2 WBS Dup

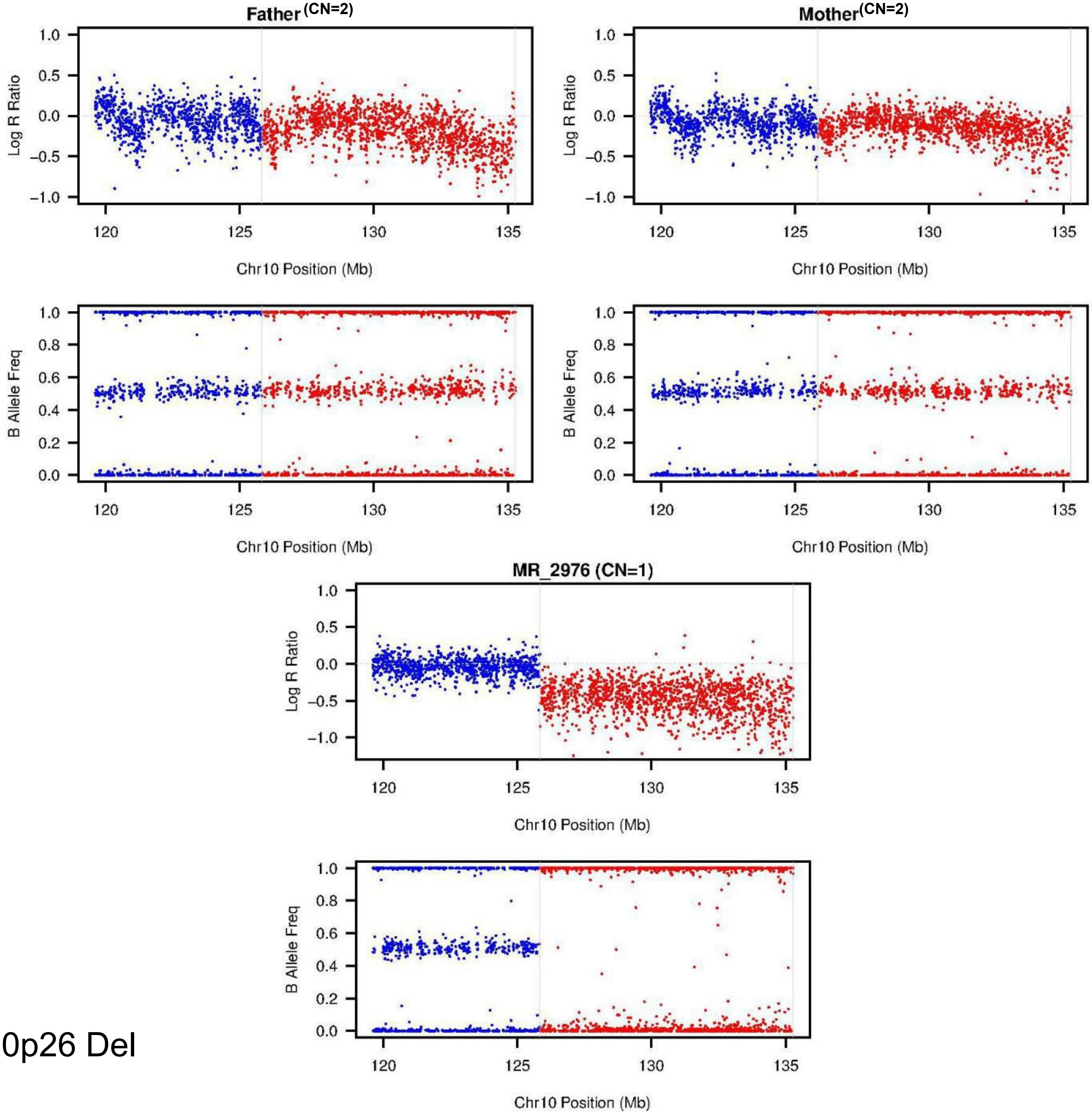

MR_2976: 10p26 Del

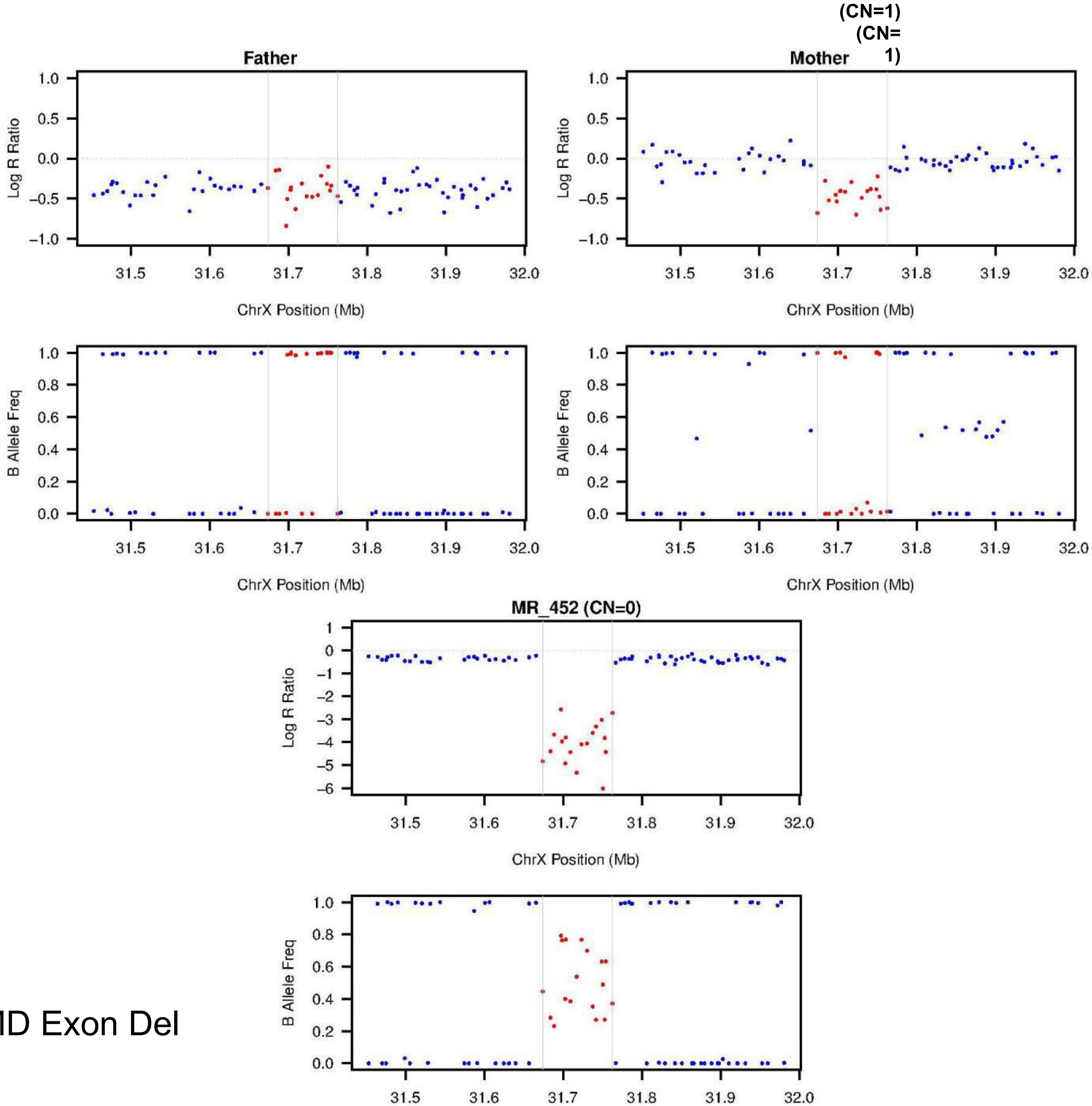

MR_452: DMD Exon Del

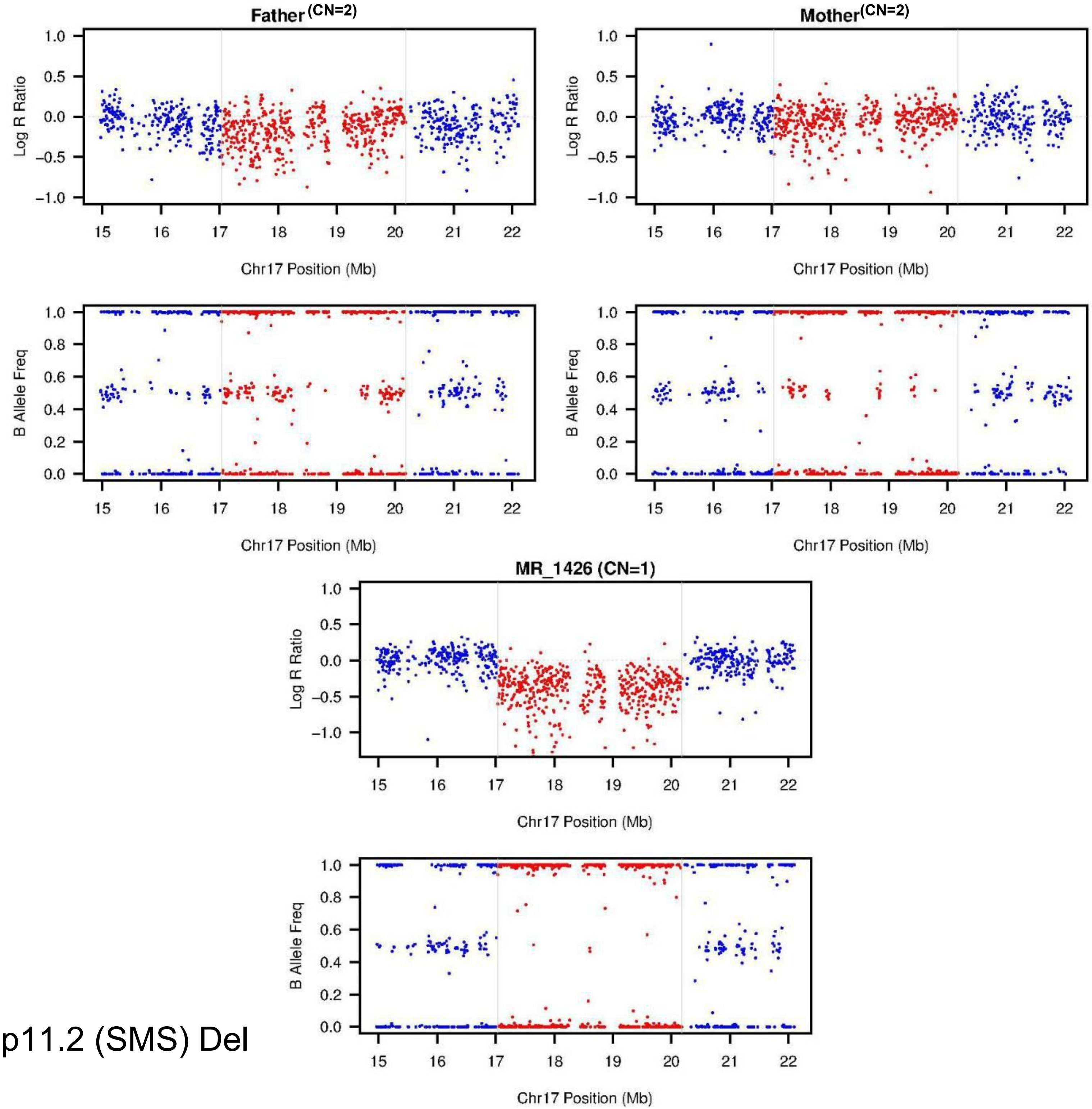

MR_1426: 17p11.2 (SMS) Del

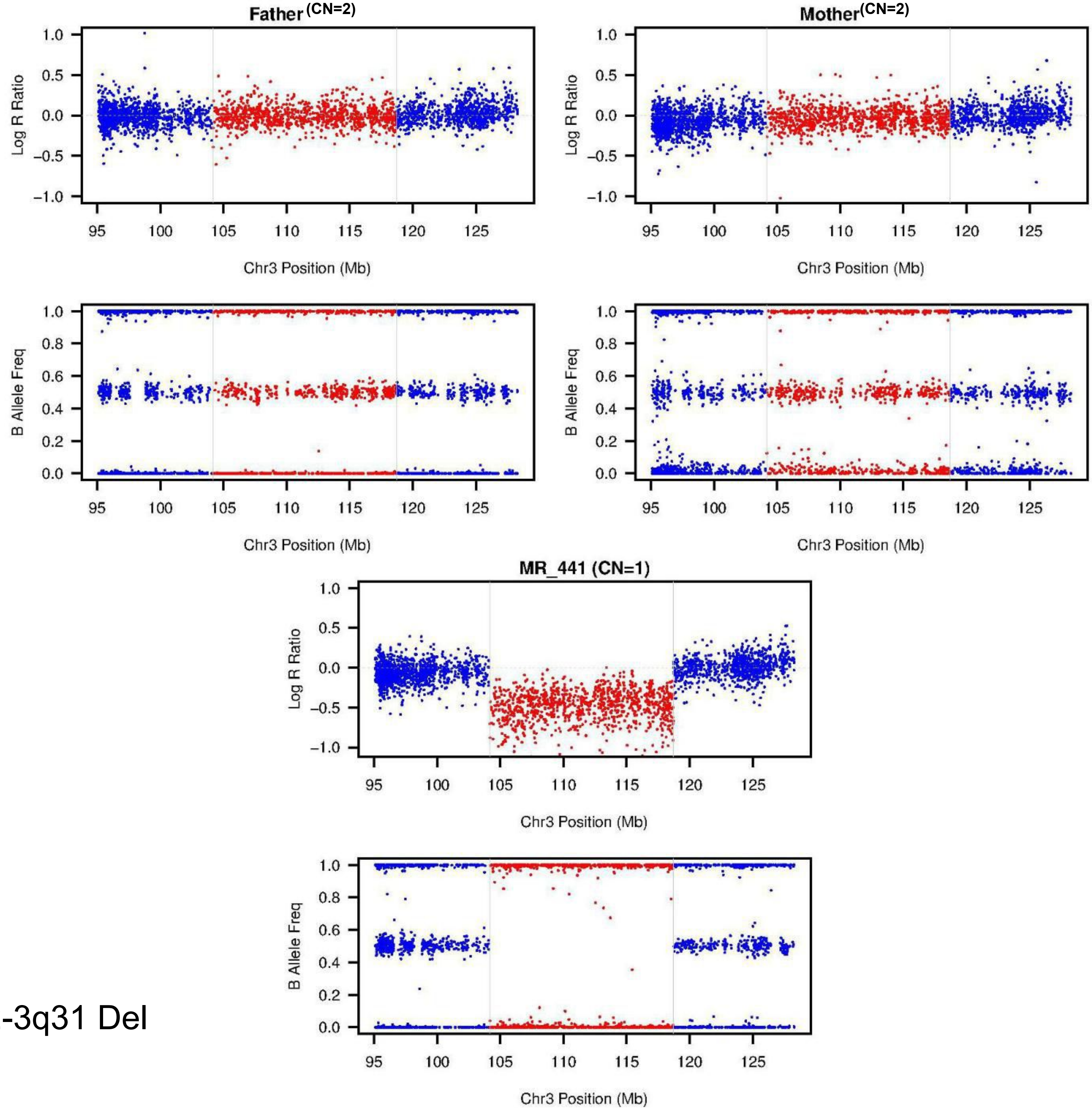

MR_441: 3q12-3q31 Del

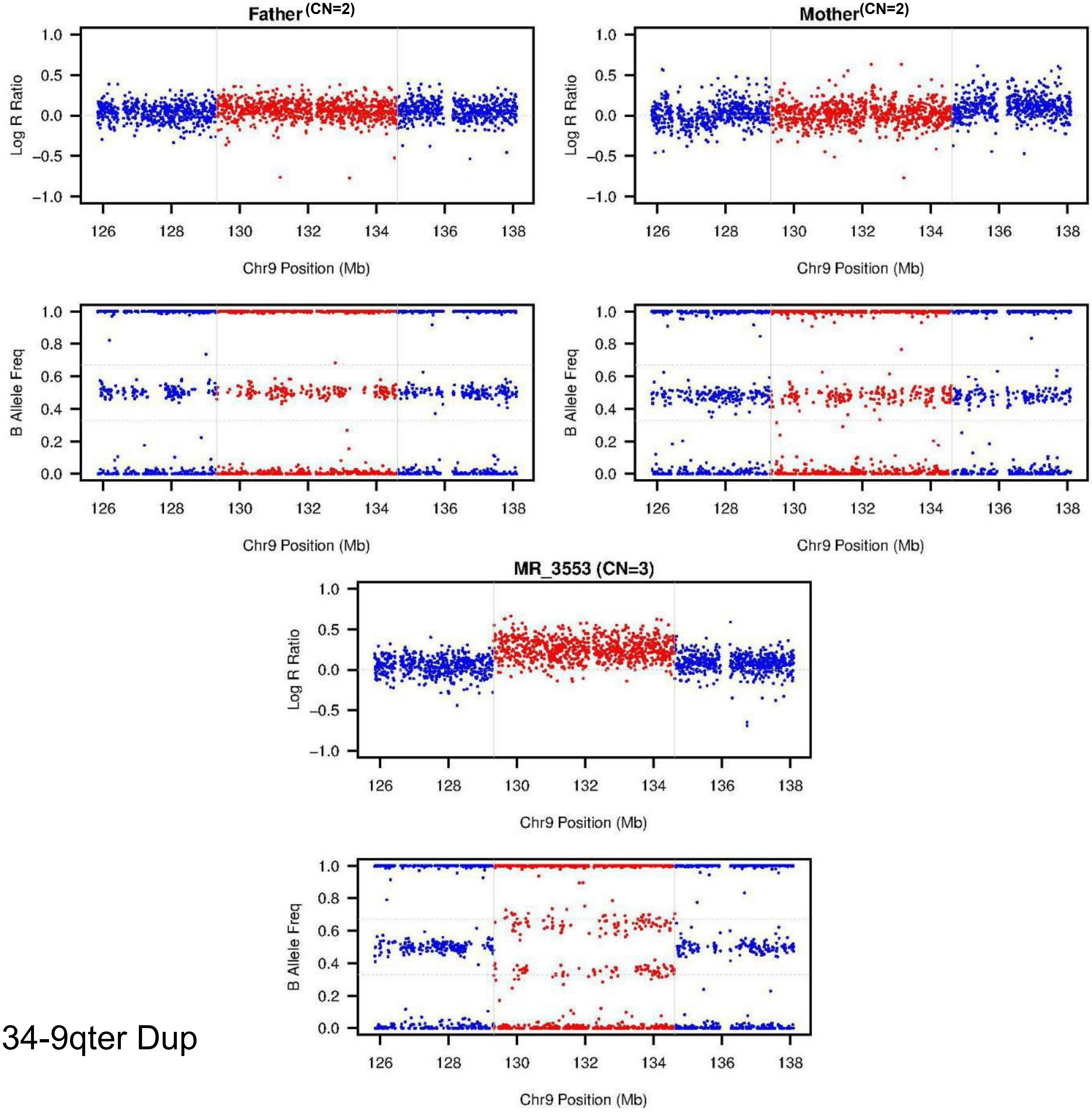

MR_3553: 9q34-9qter Dup

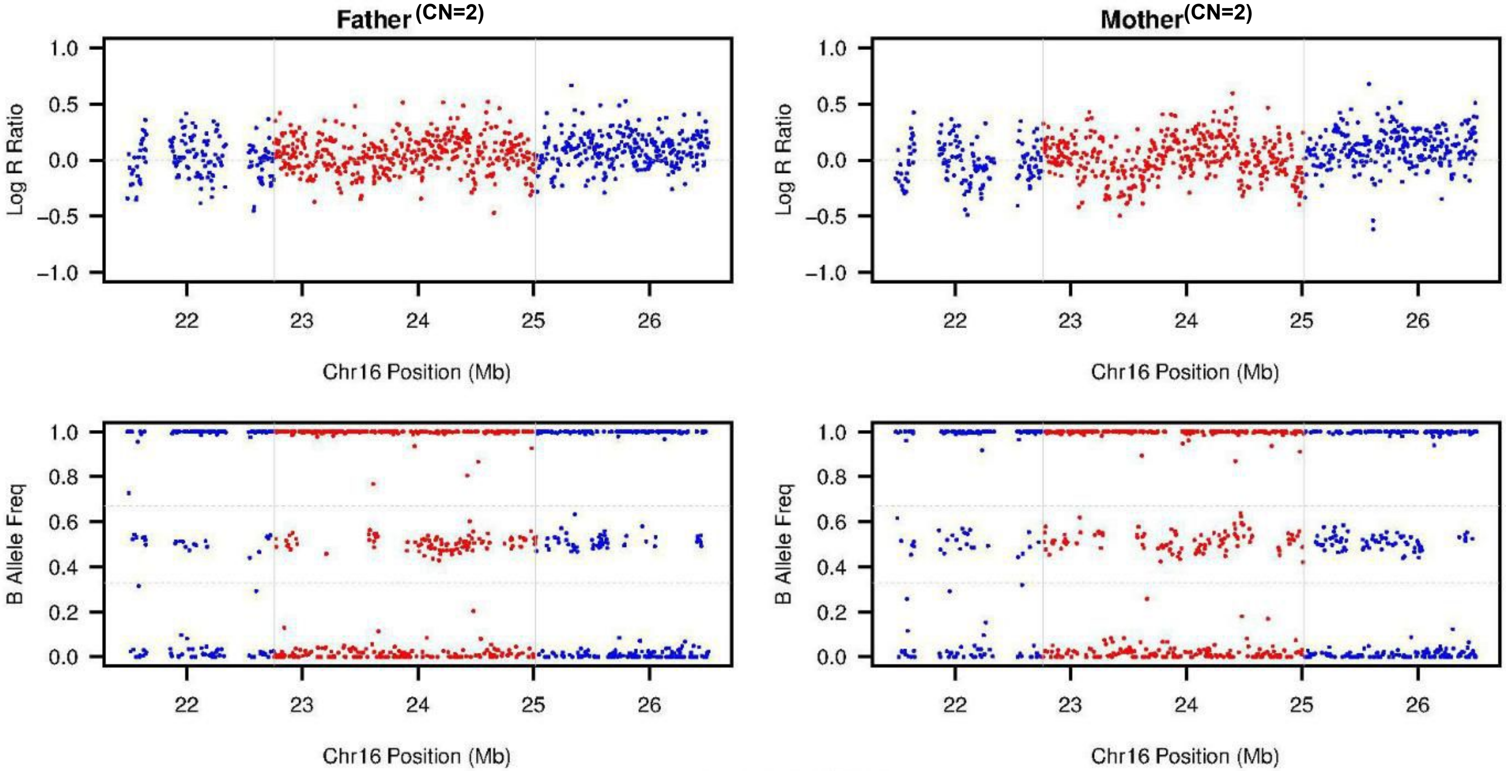

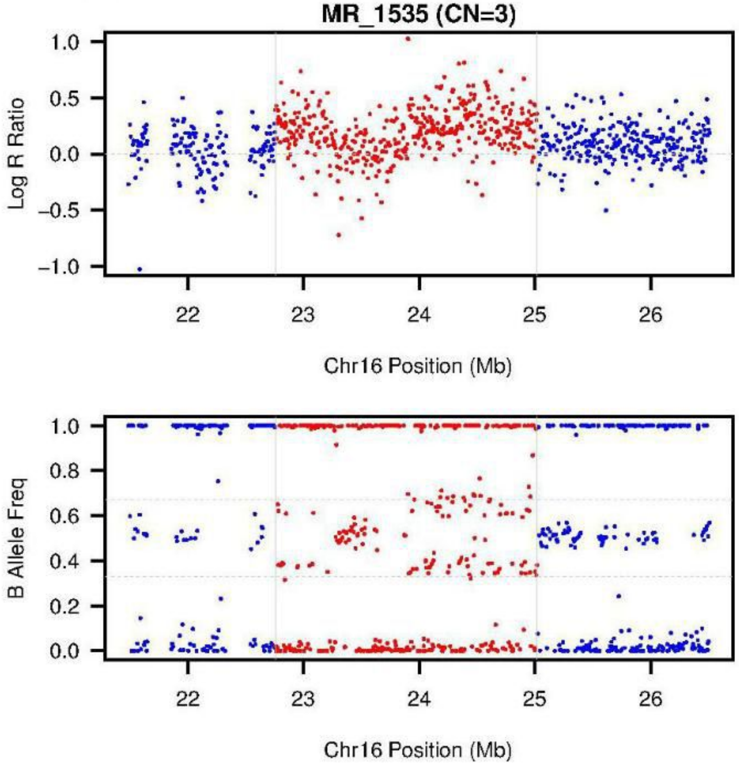

MR_1535: 16p12.2-p11.2 Dup

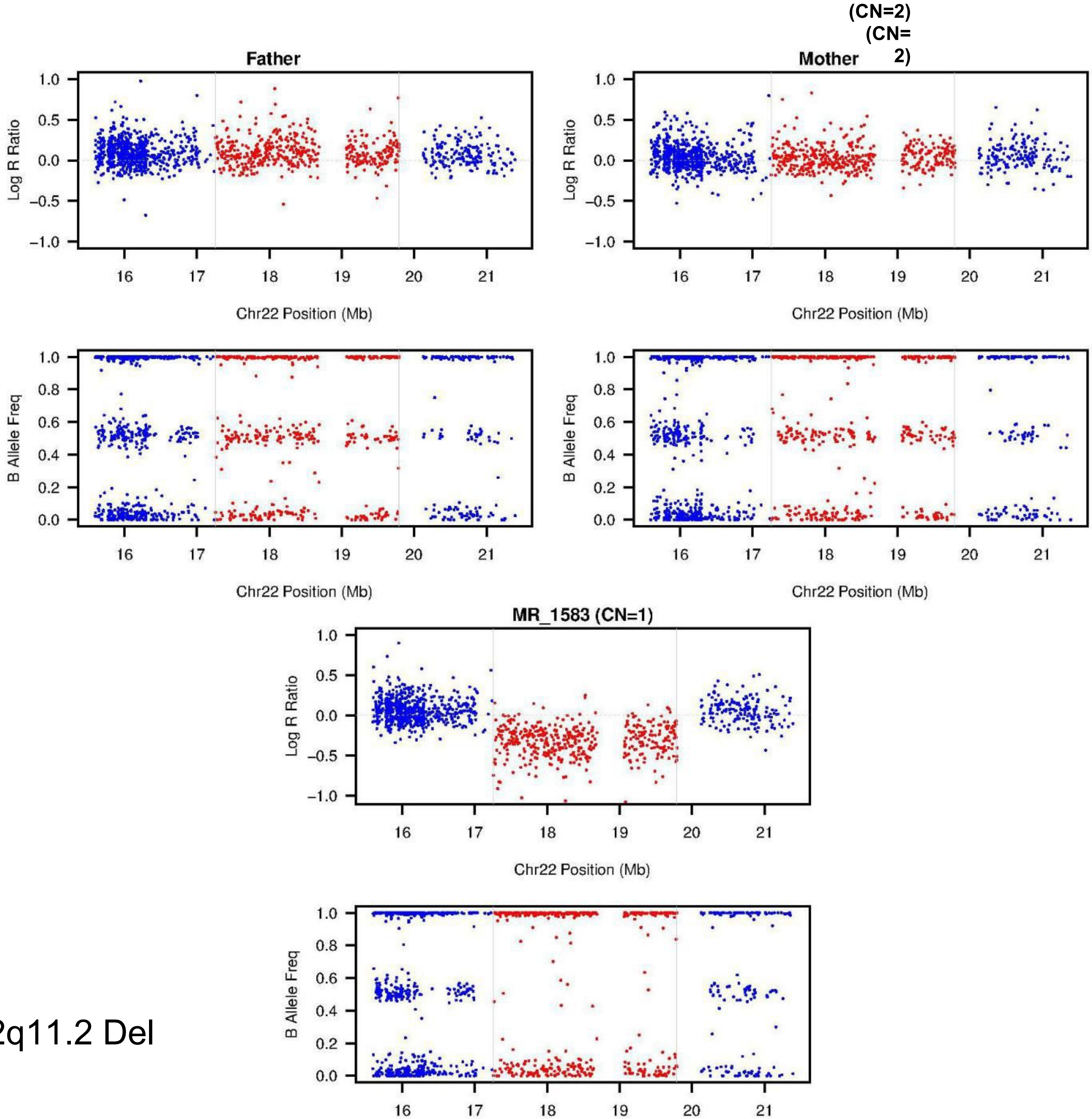

MR_1583: 22q11.2 Del

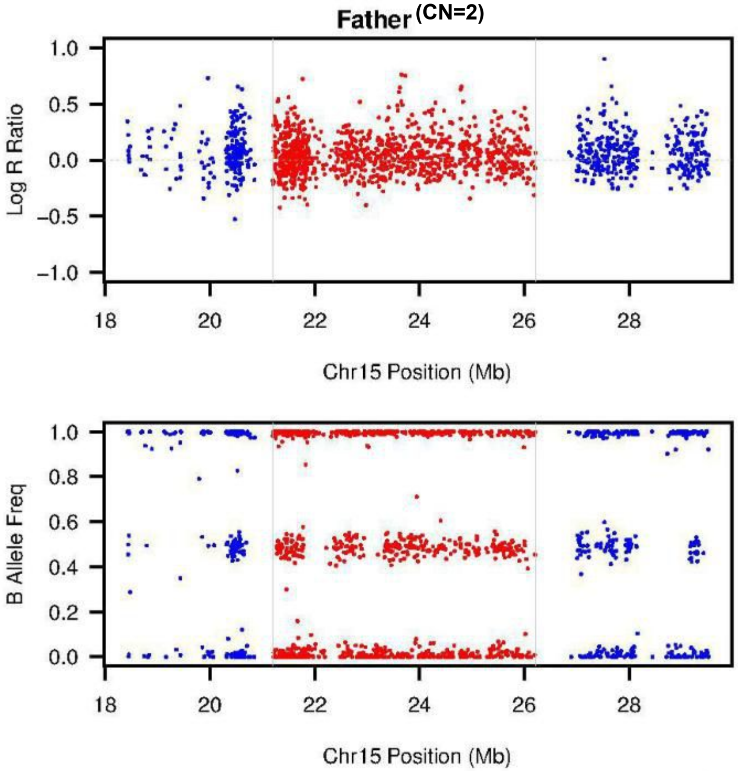

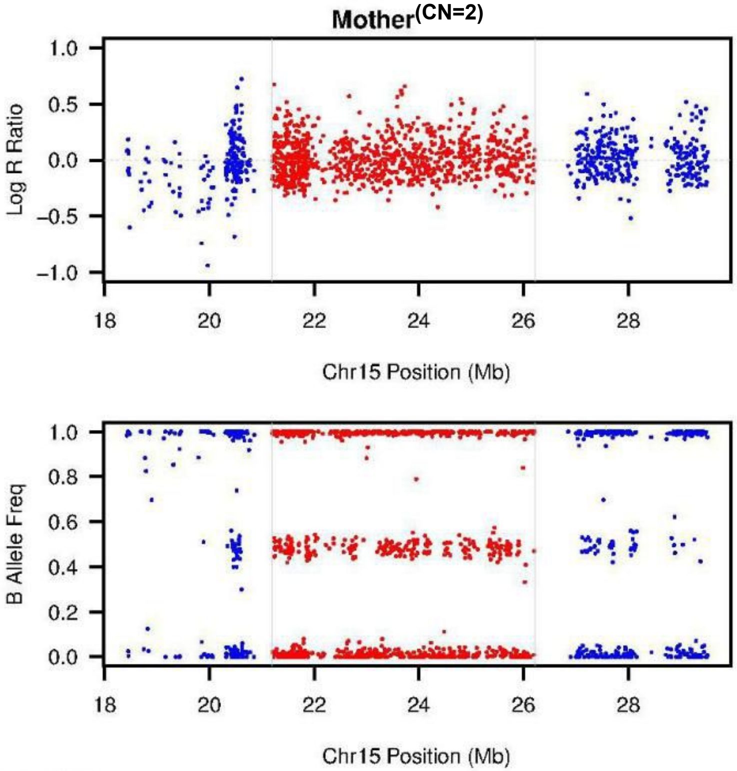

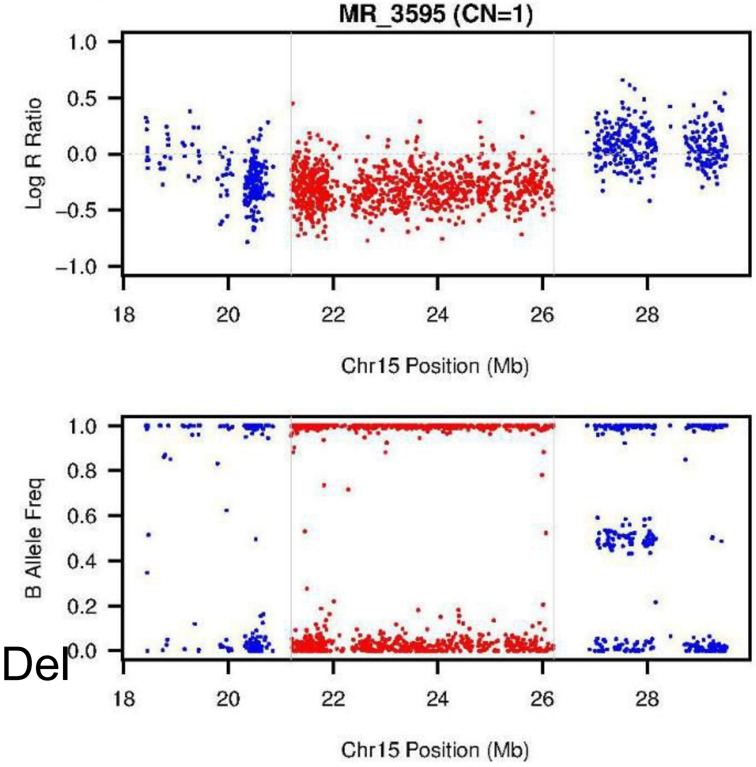

MR_3595: 15q11-q31(Angelman) Del

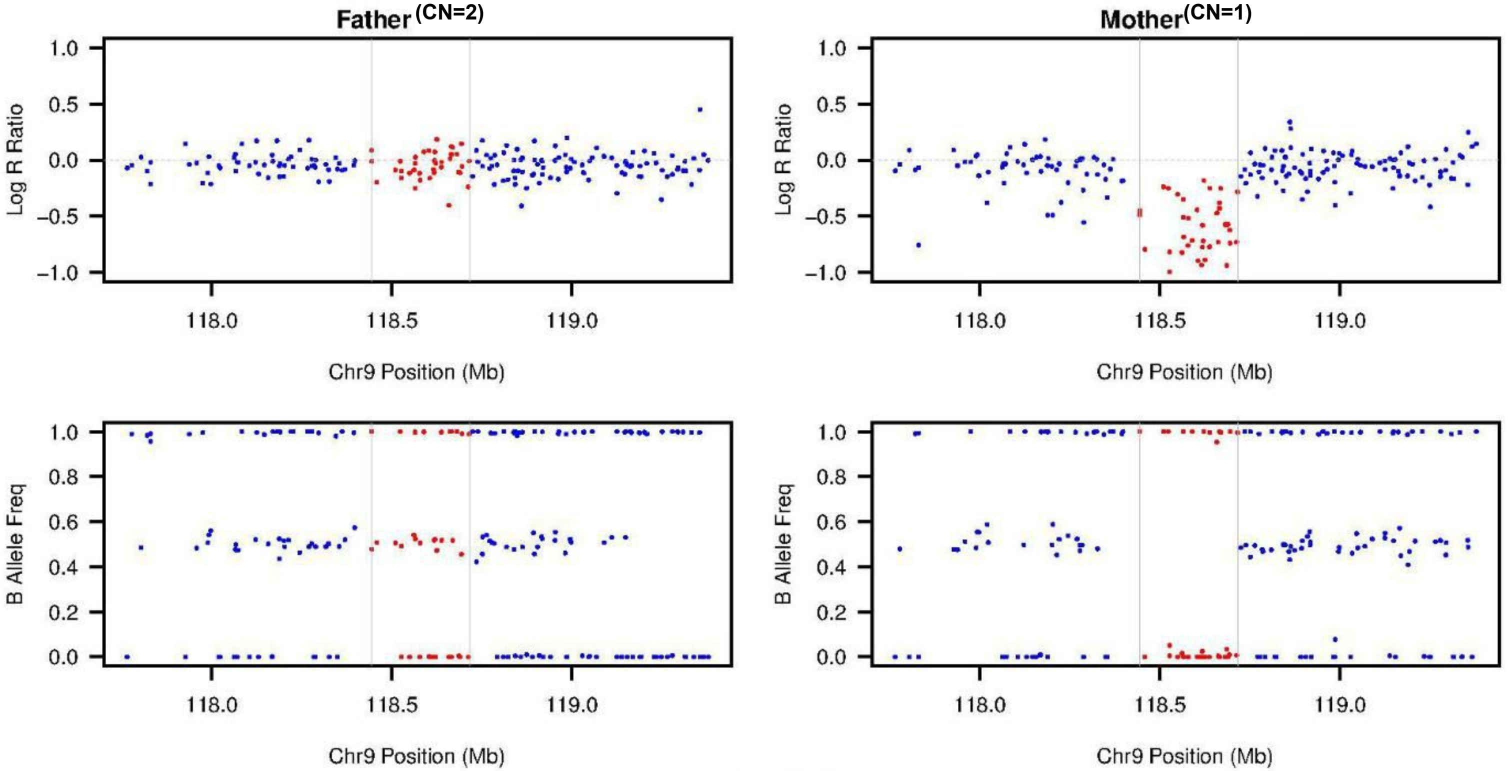

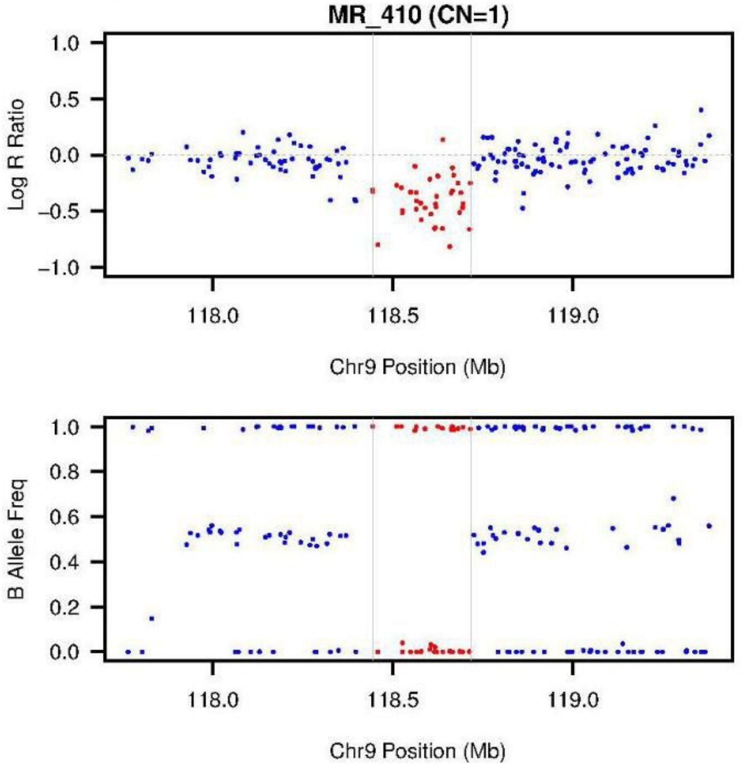

MR_410: 9q33 Del

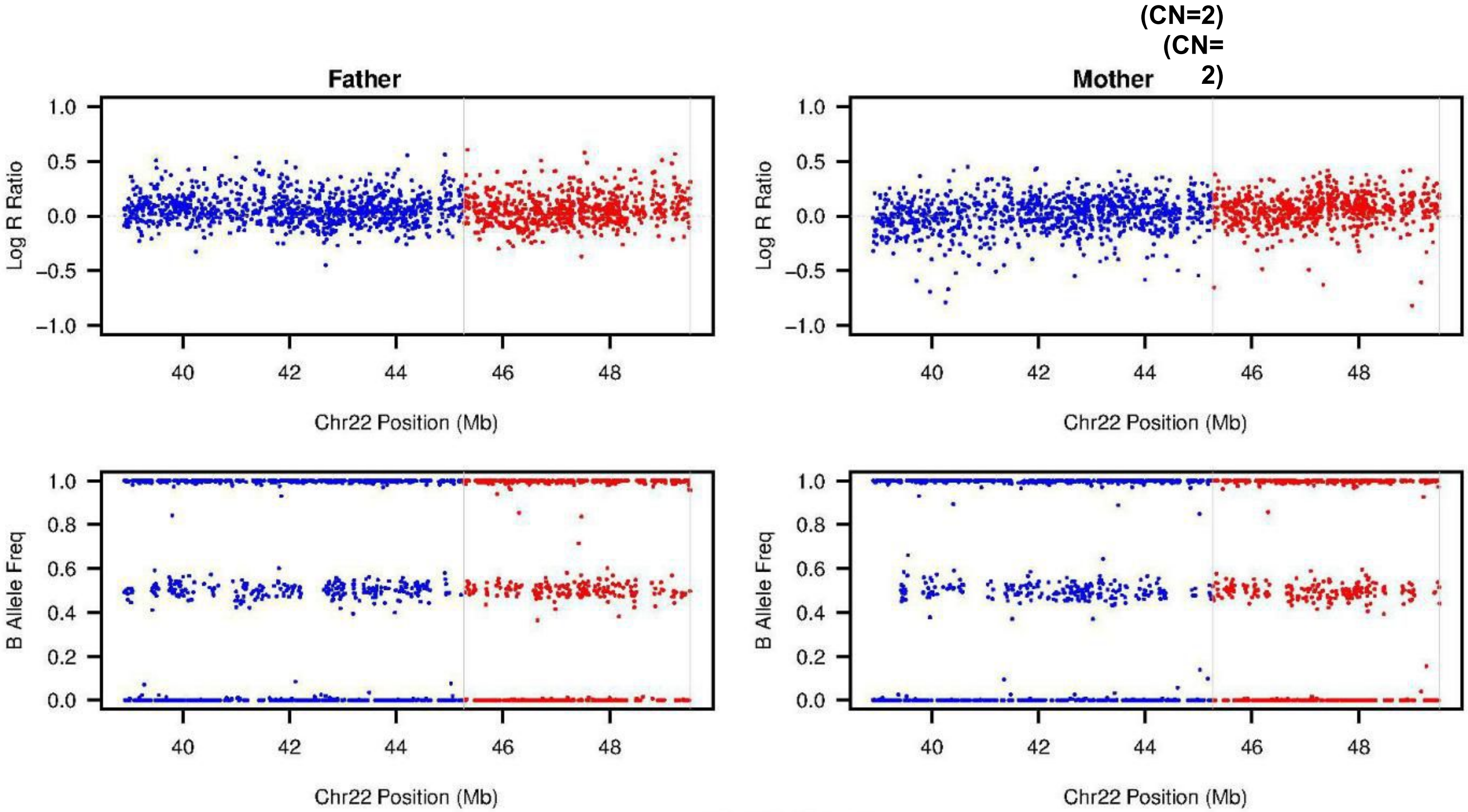

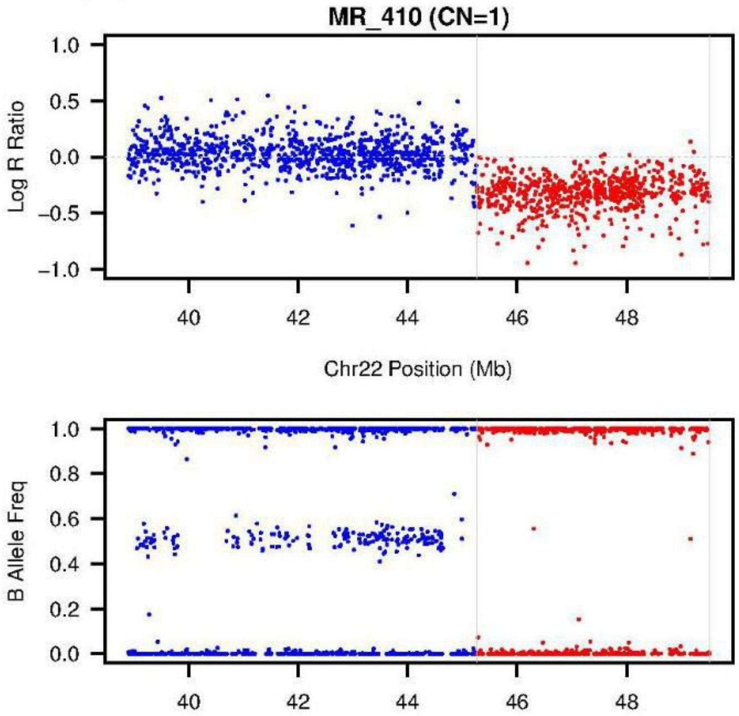

MR_410: 22q13.2-22qter Del

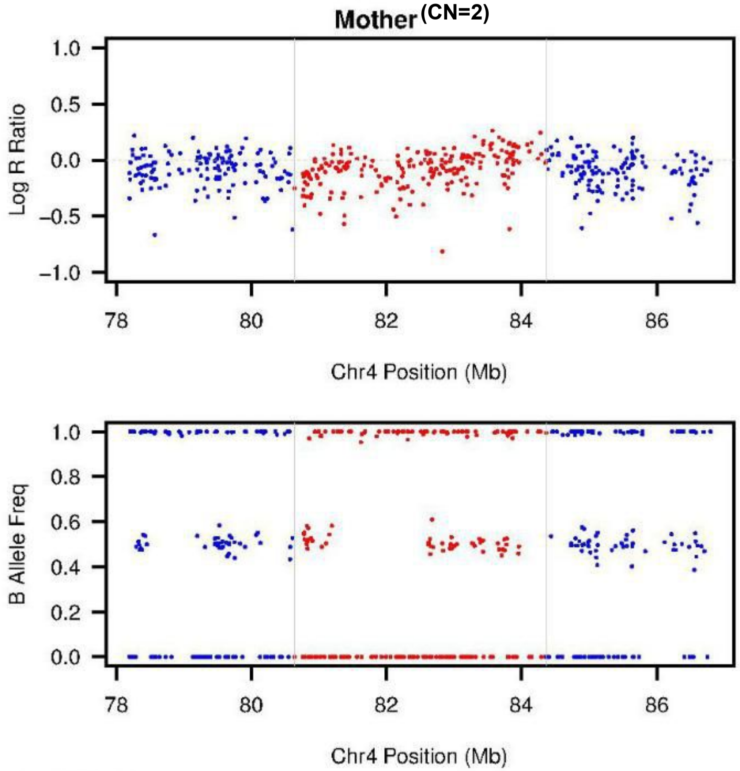

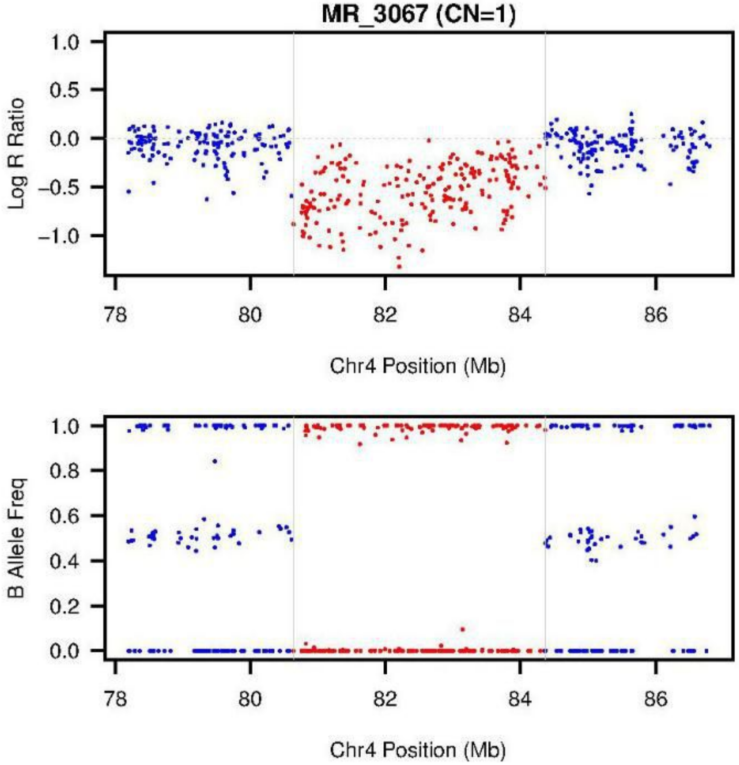

MR_3067: 4q21 Del

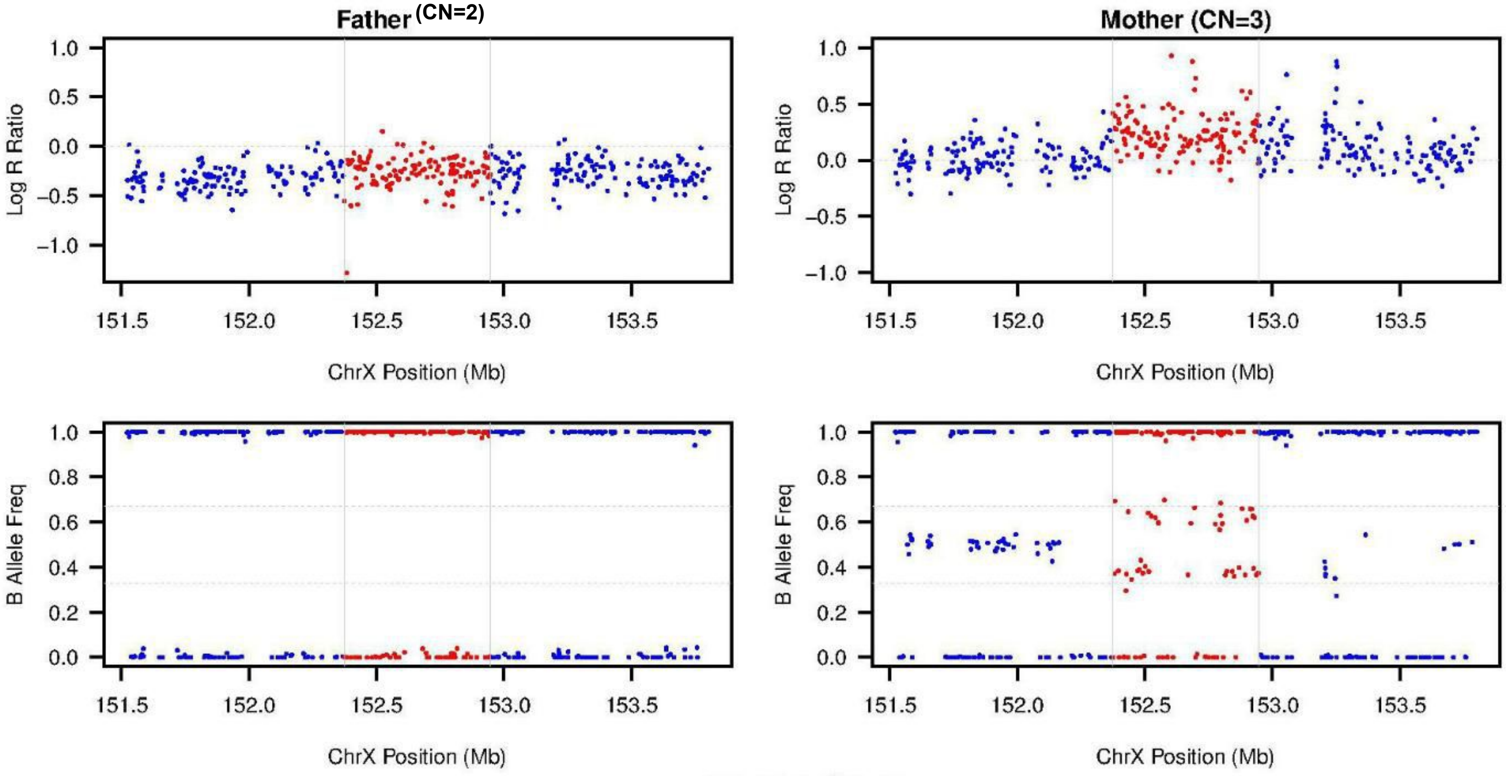

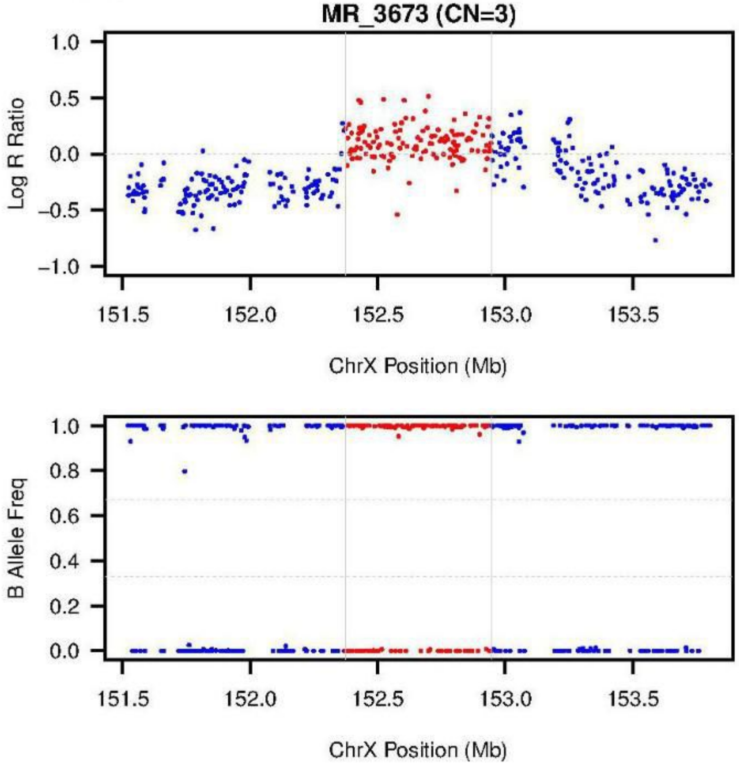

MR_3673: Xq28 MECP2 Dup

(CN=2)
(CN=
2)

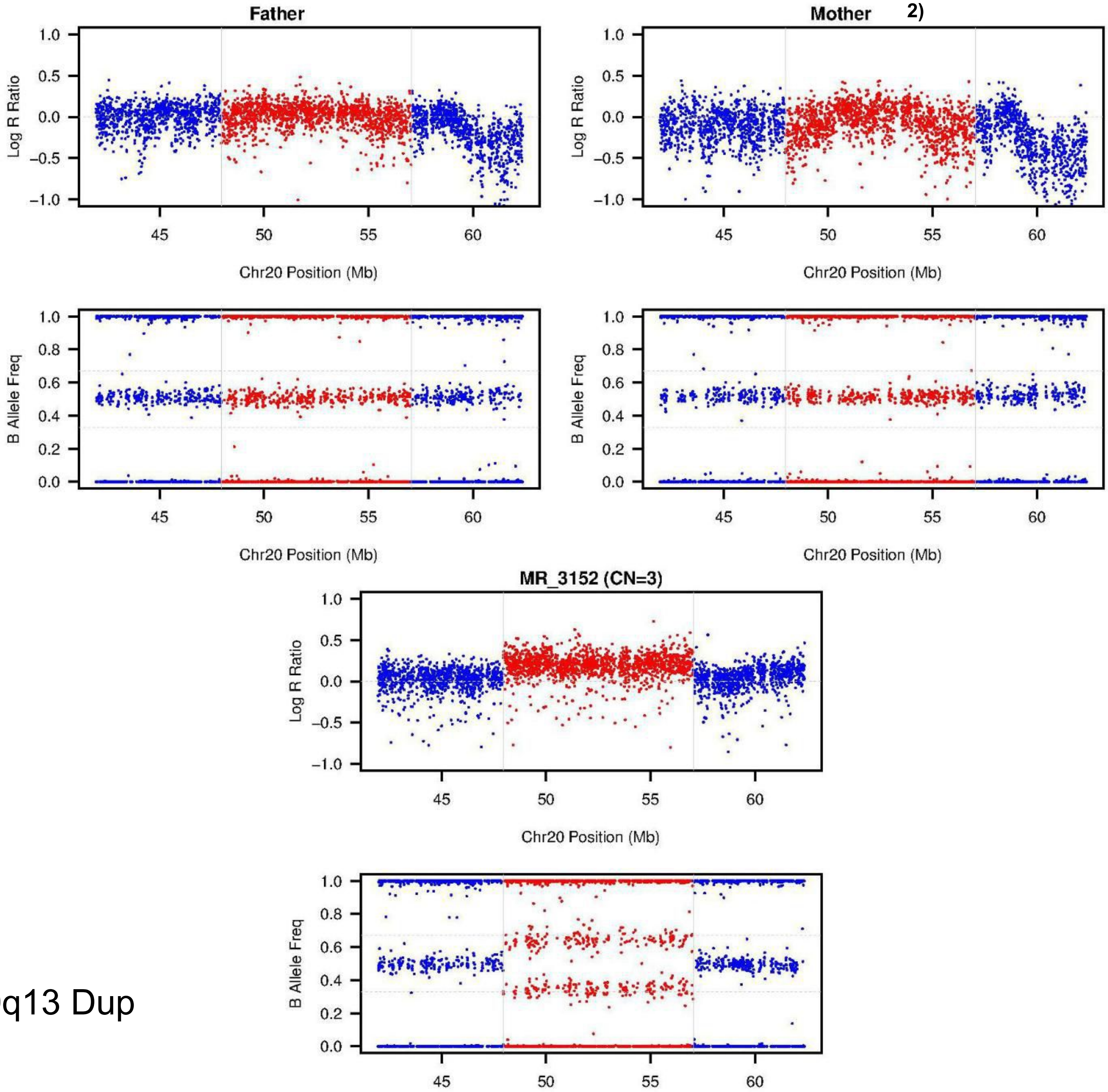

MR_3152: 20q13 Dup

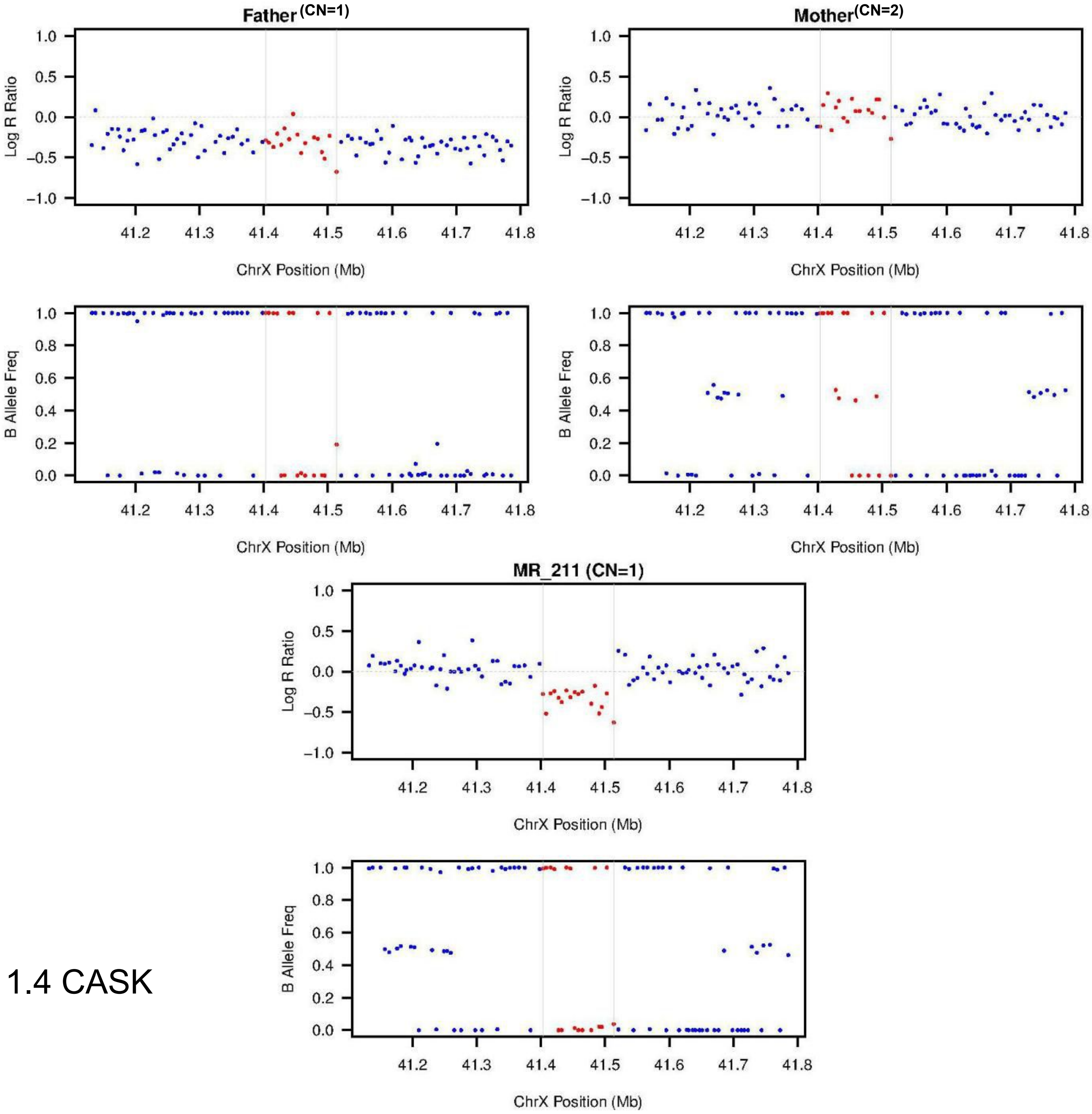

MR_211: Xp11.4 CASK Del

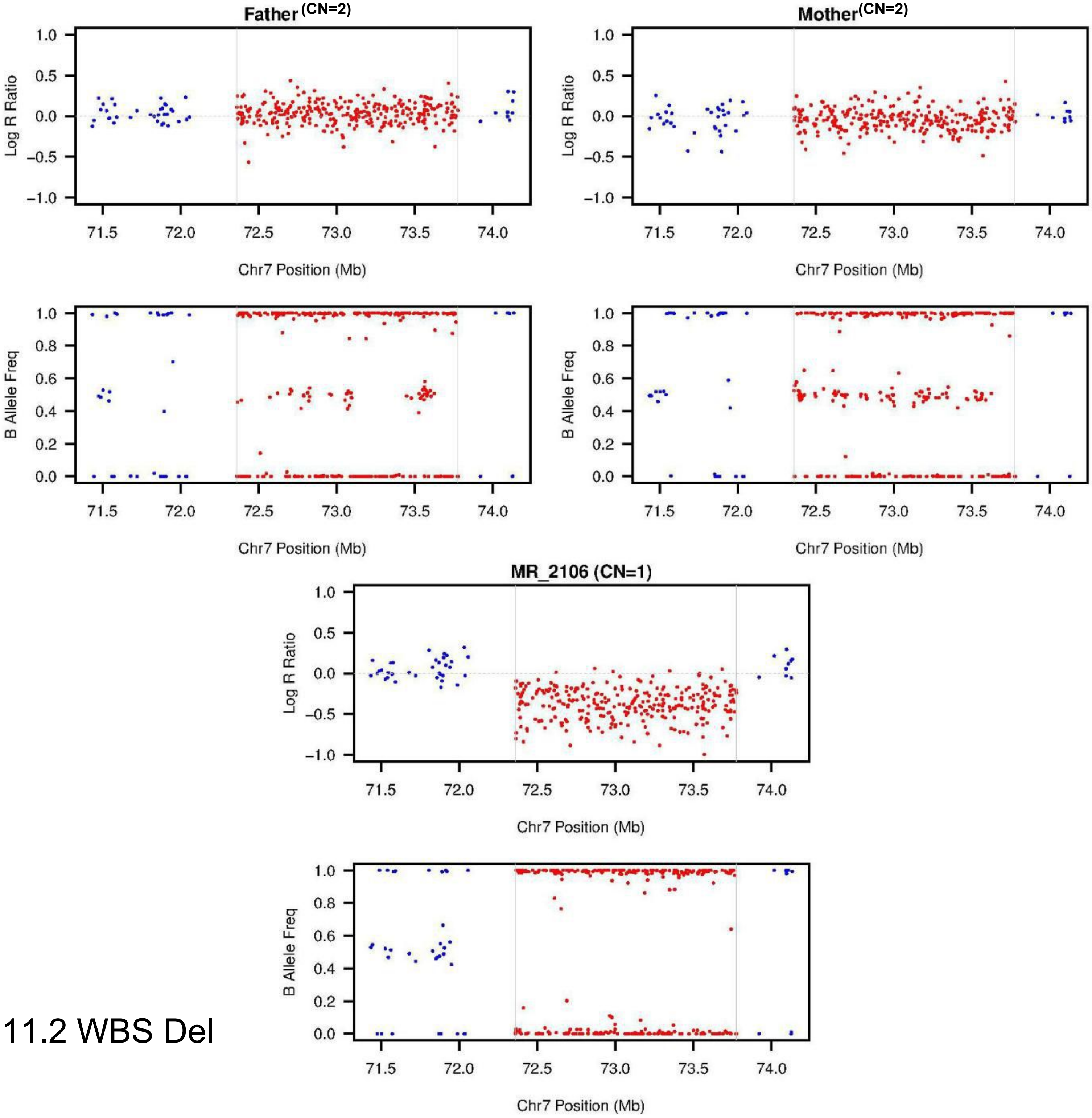

MR_2106: 7q11.2 WBS Del

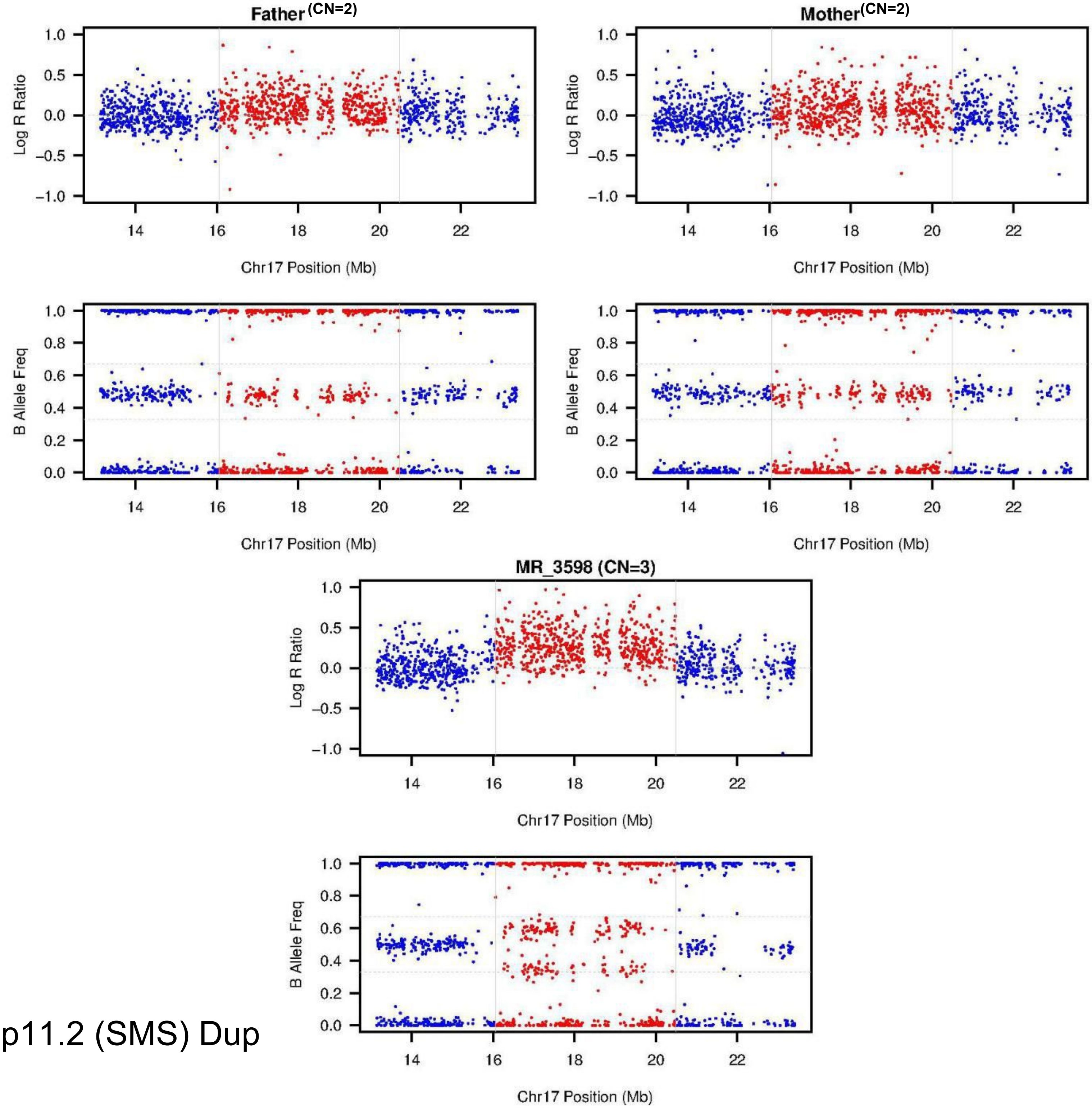

MR_3598: 17p11.2 (SMS) Dup

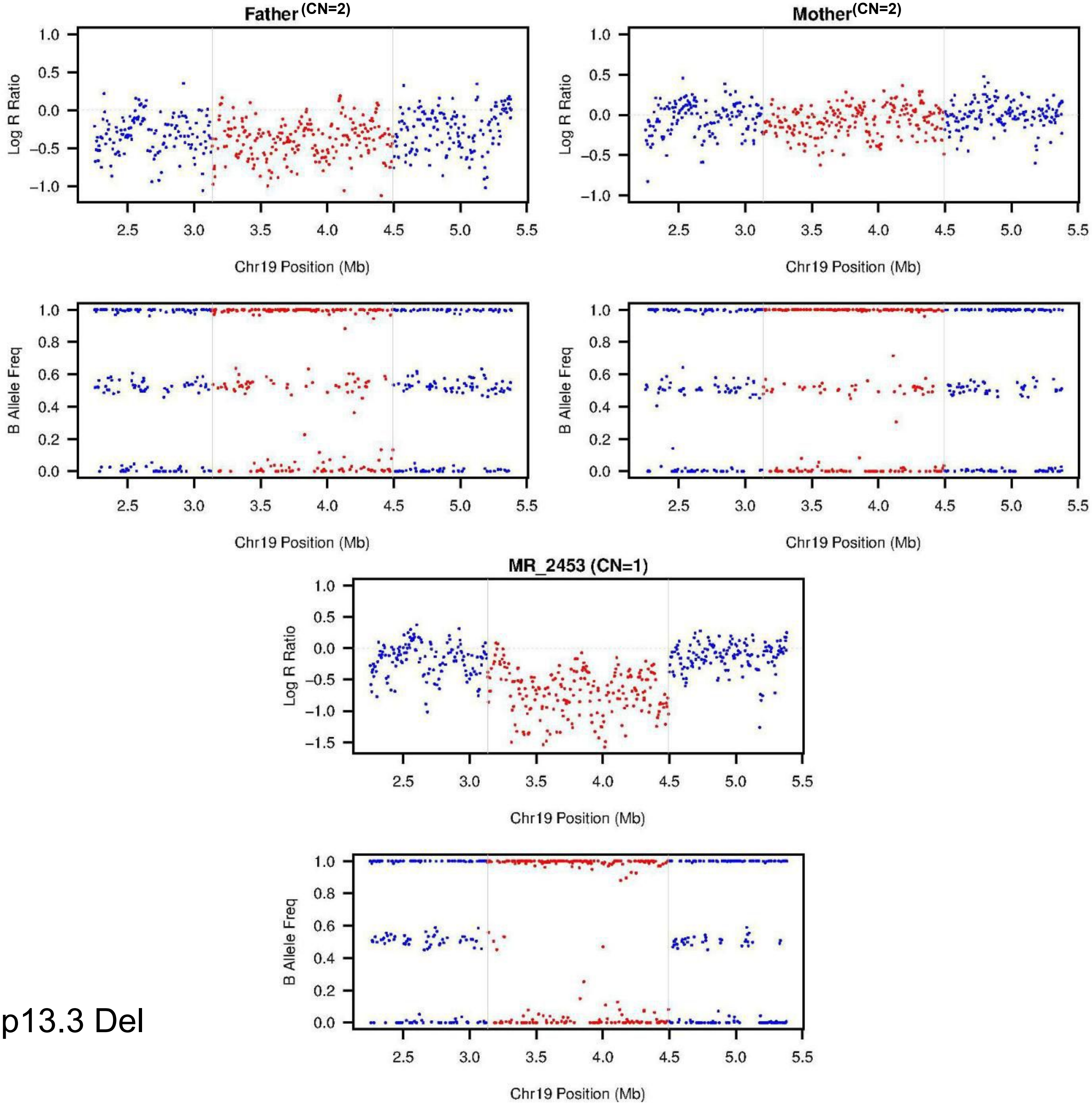

MR_2453: 19p13.3 Del

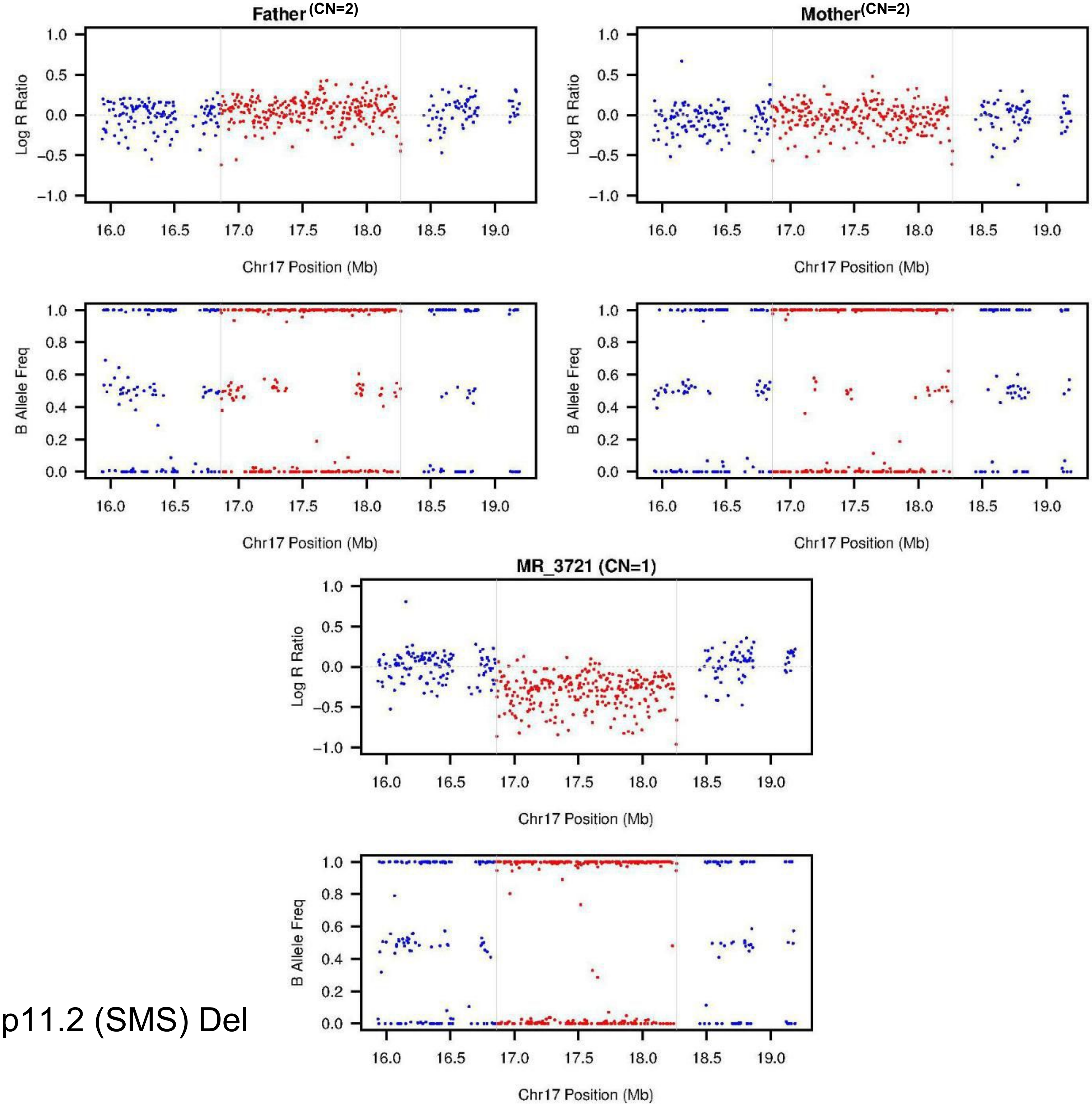

MR_3721: 17p11.2 (SMS) Del

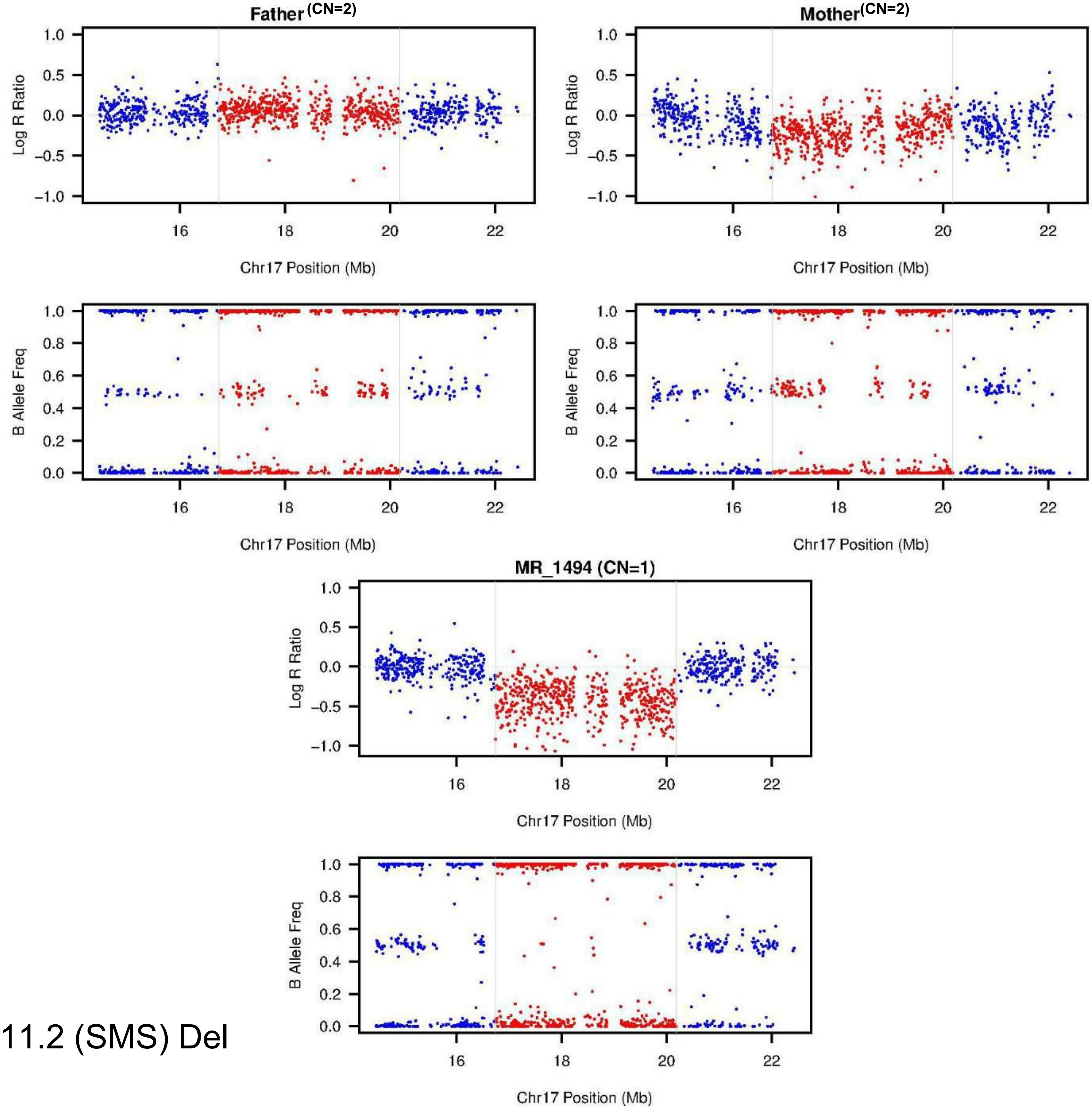

MR_1494: 17p11.2 (SMS) Del

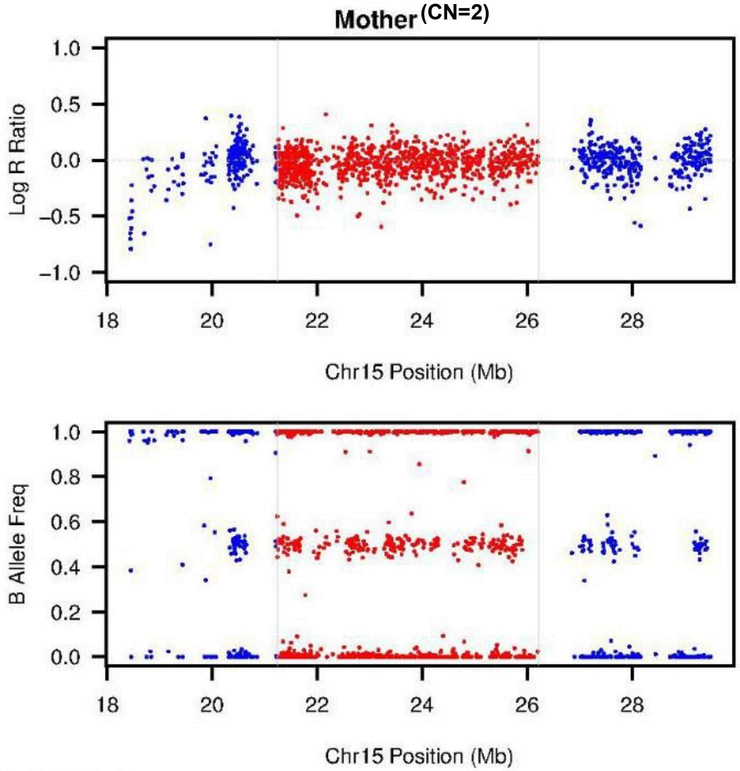

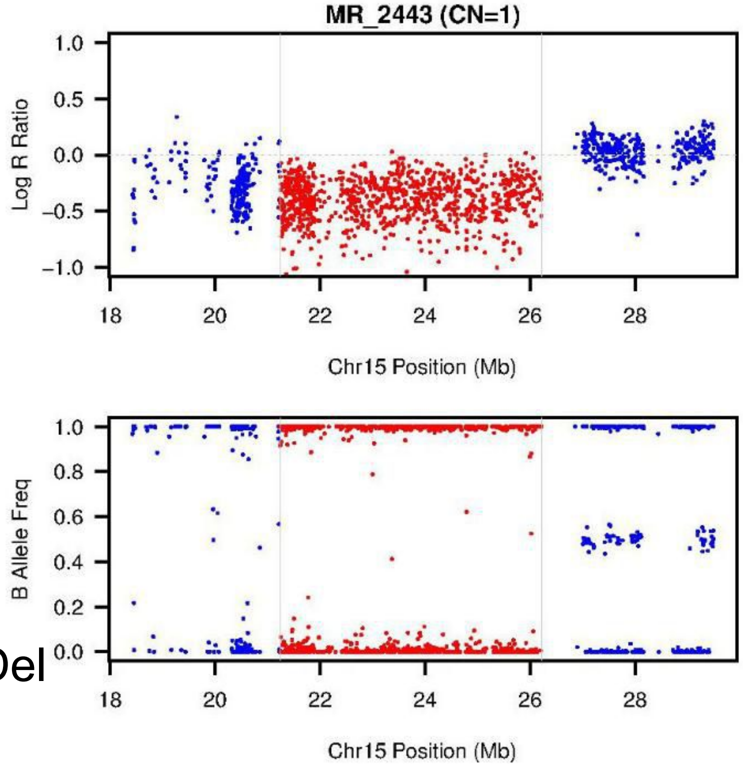

MR_2443: 15q11-q31(Angelman) Del

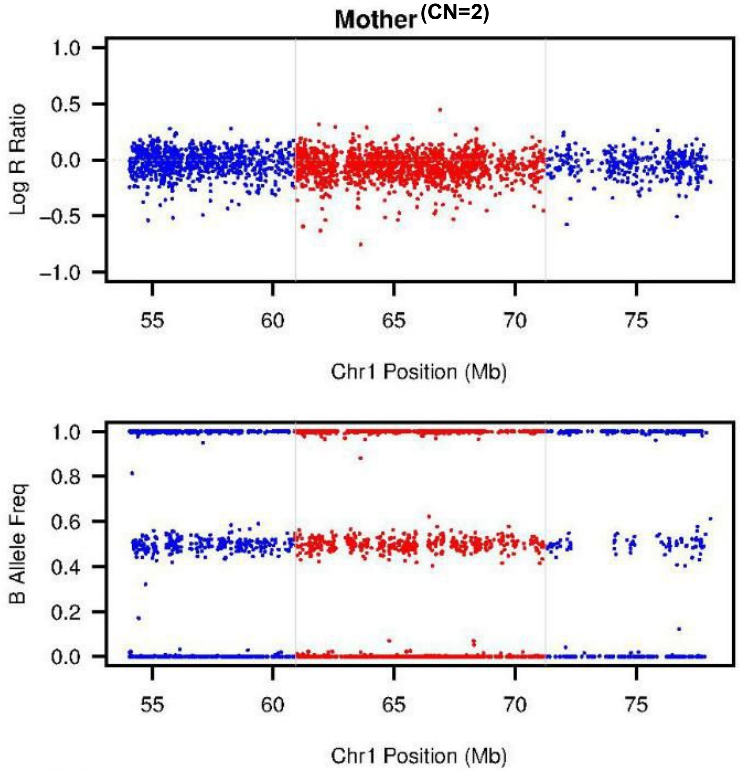

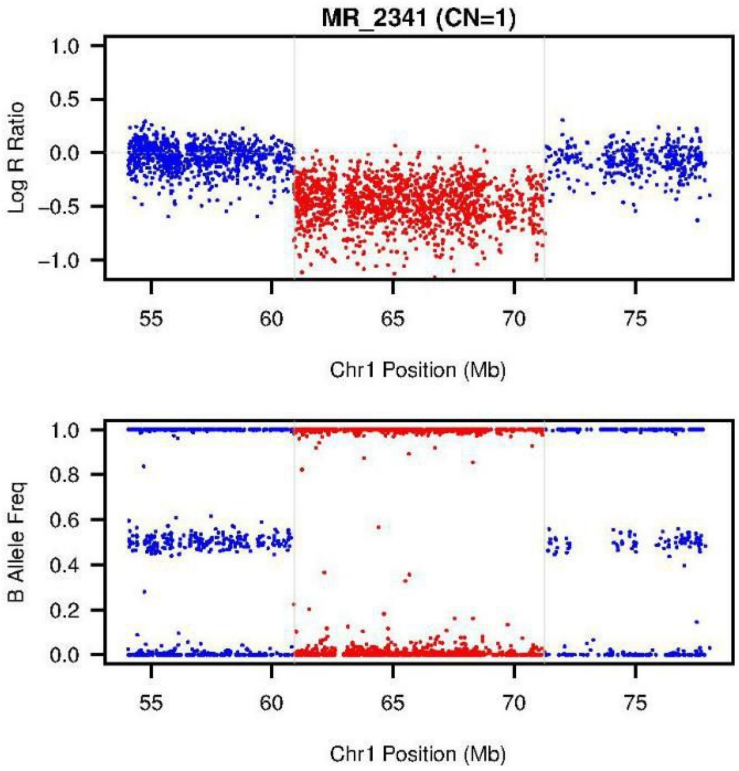

MR_2341: 1p31 Del

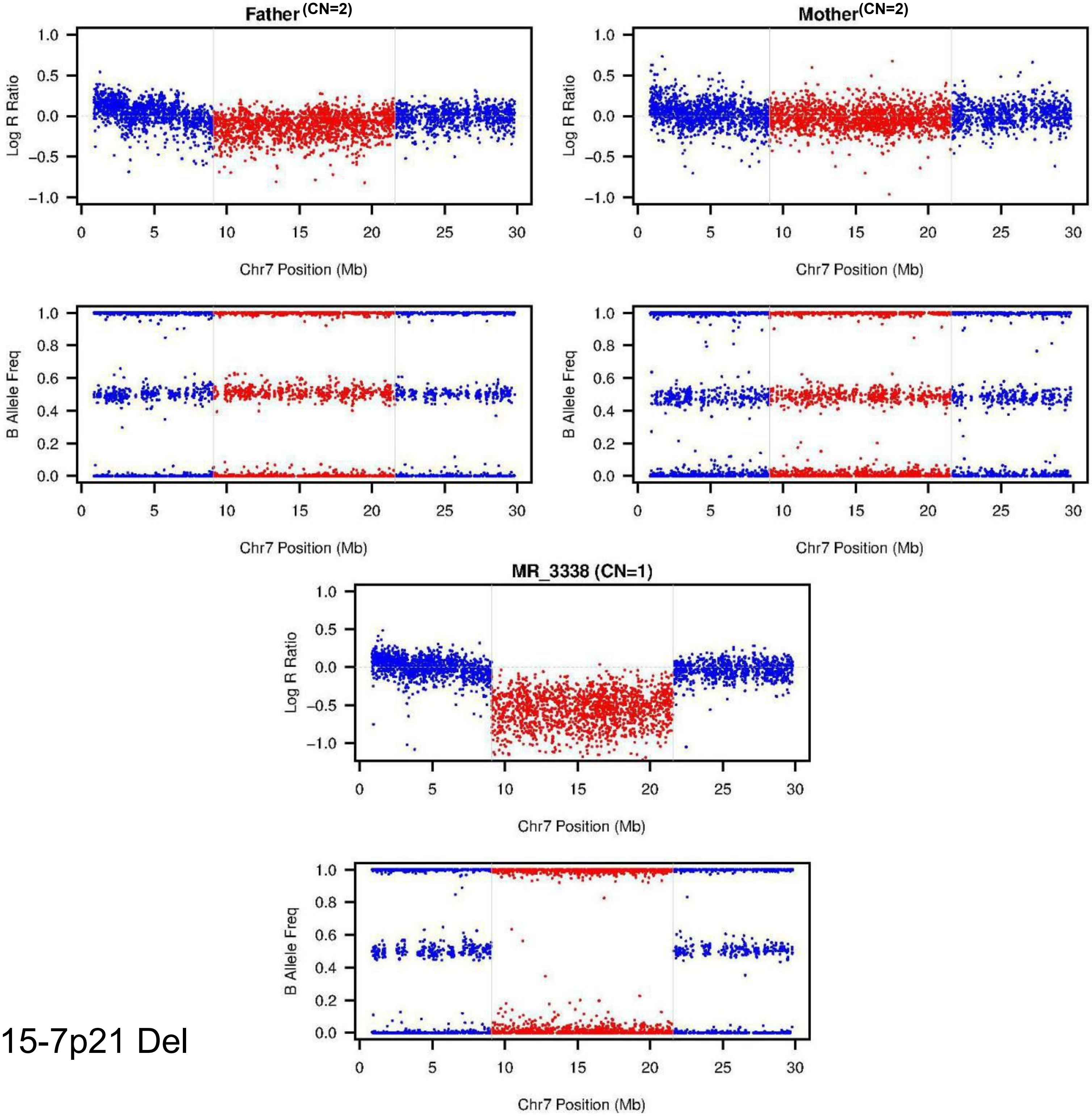

MR_3338: 7p15-7p21 Del

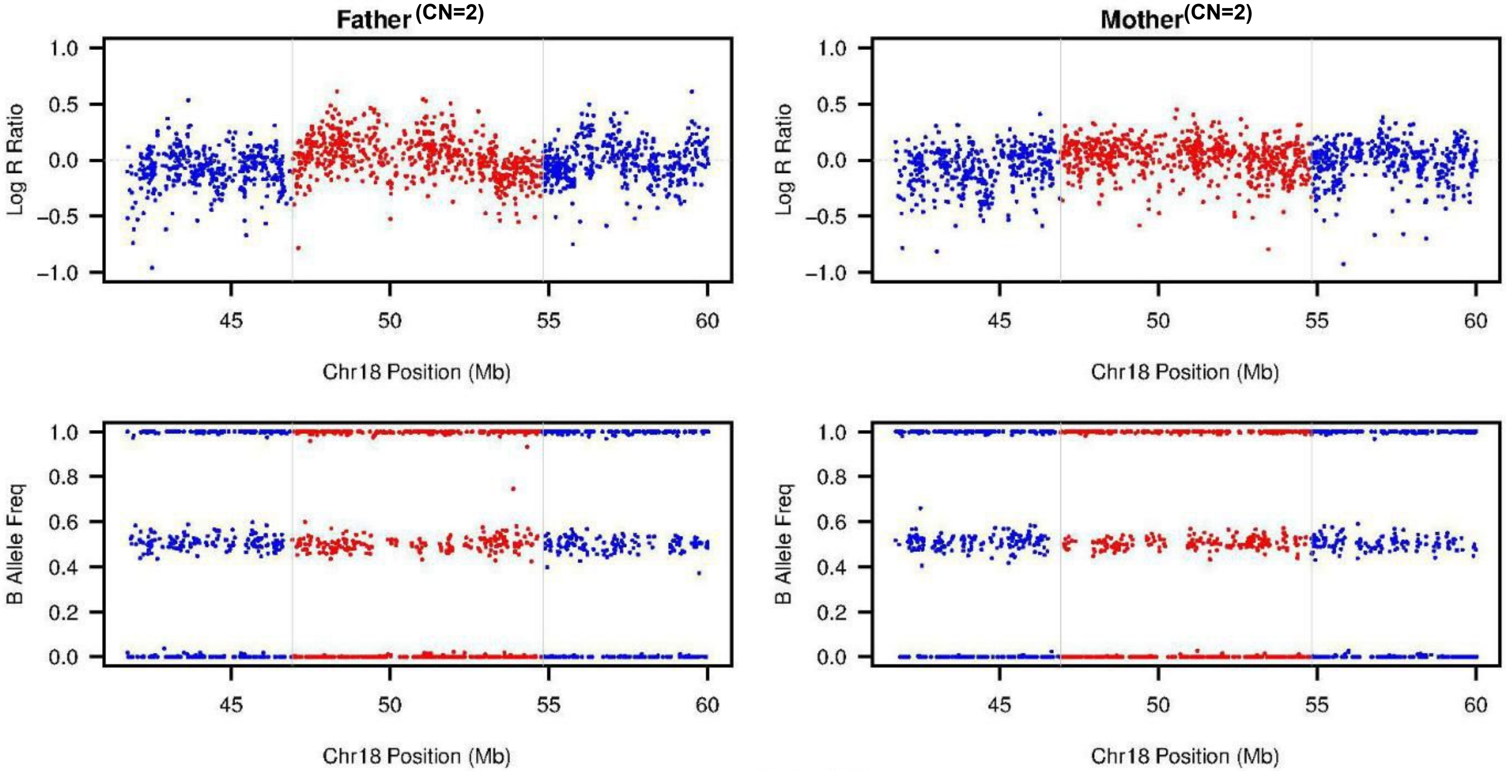

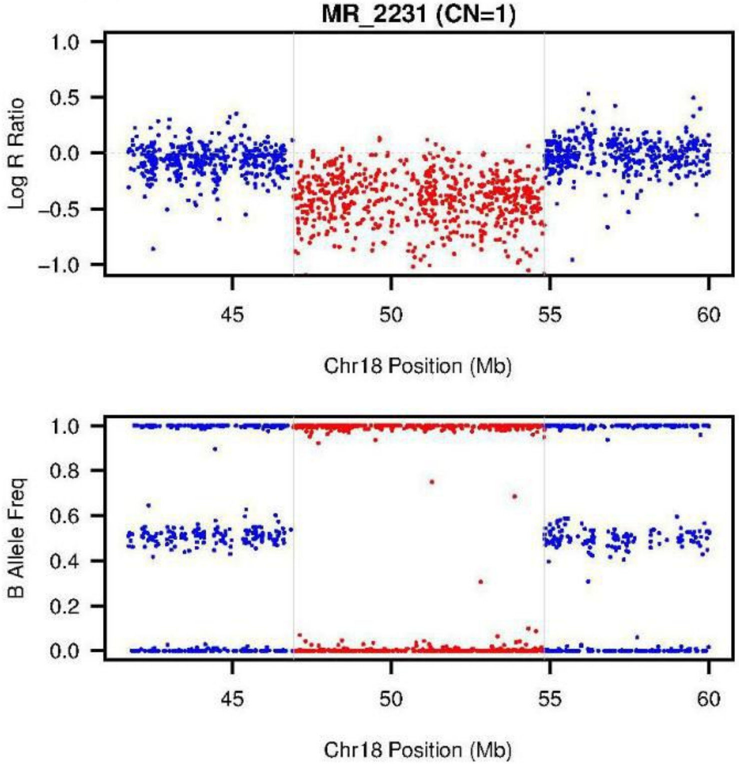

MR_2231: 18q21 Del

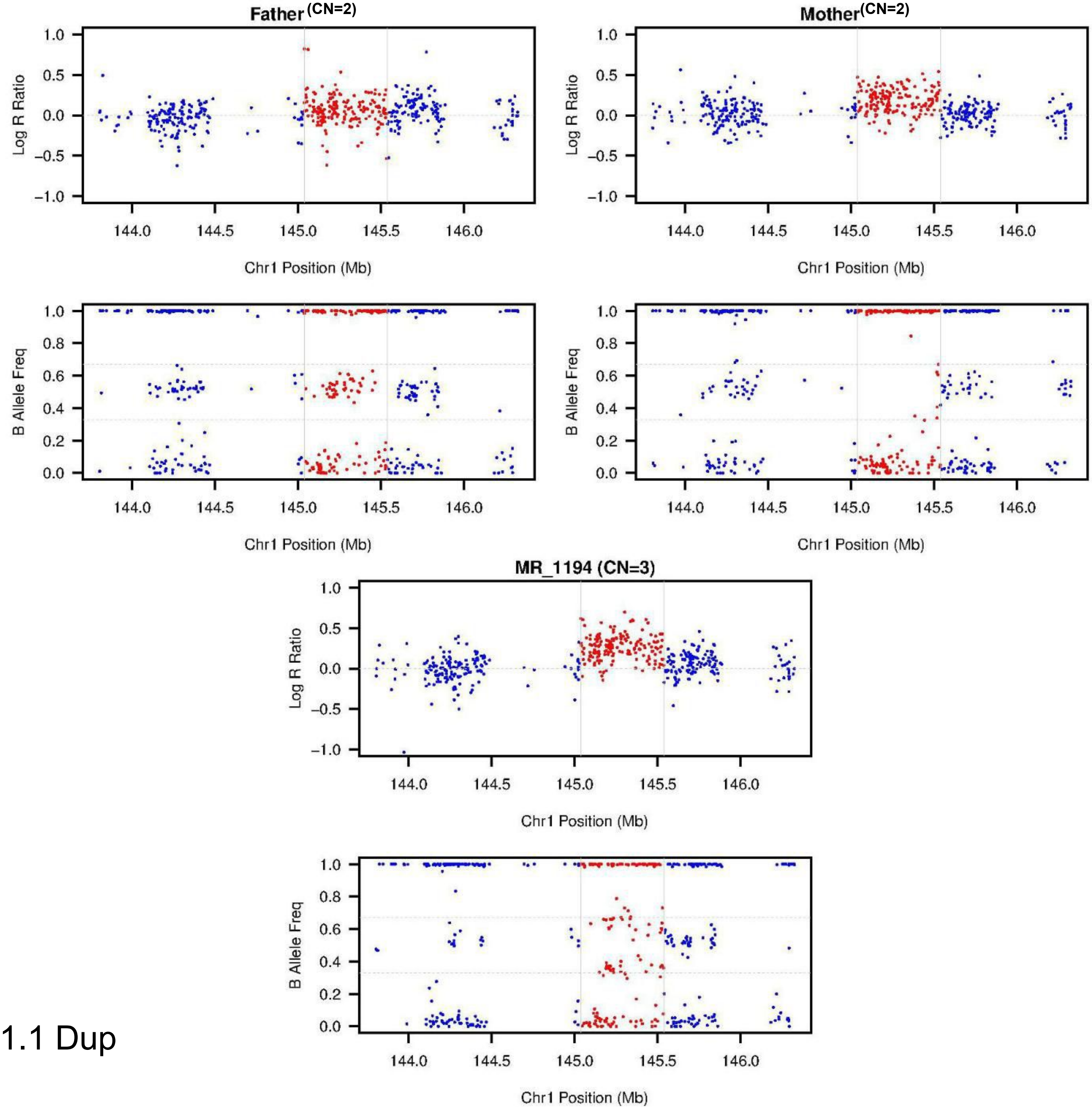

MR_1194: 1q21.1 Dup

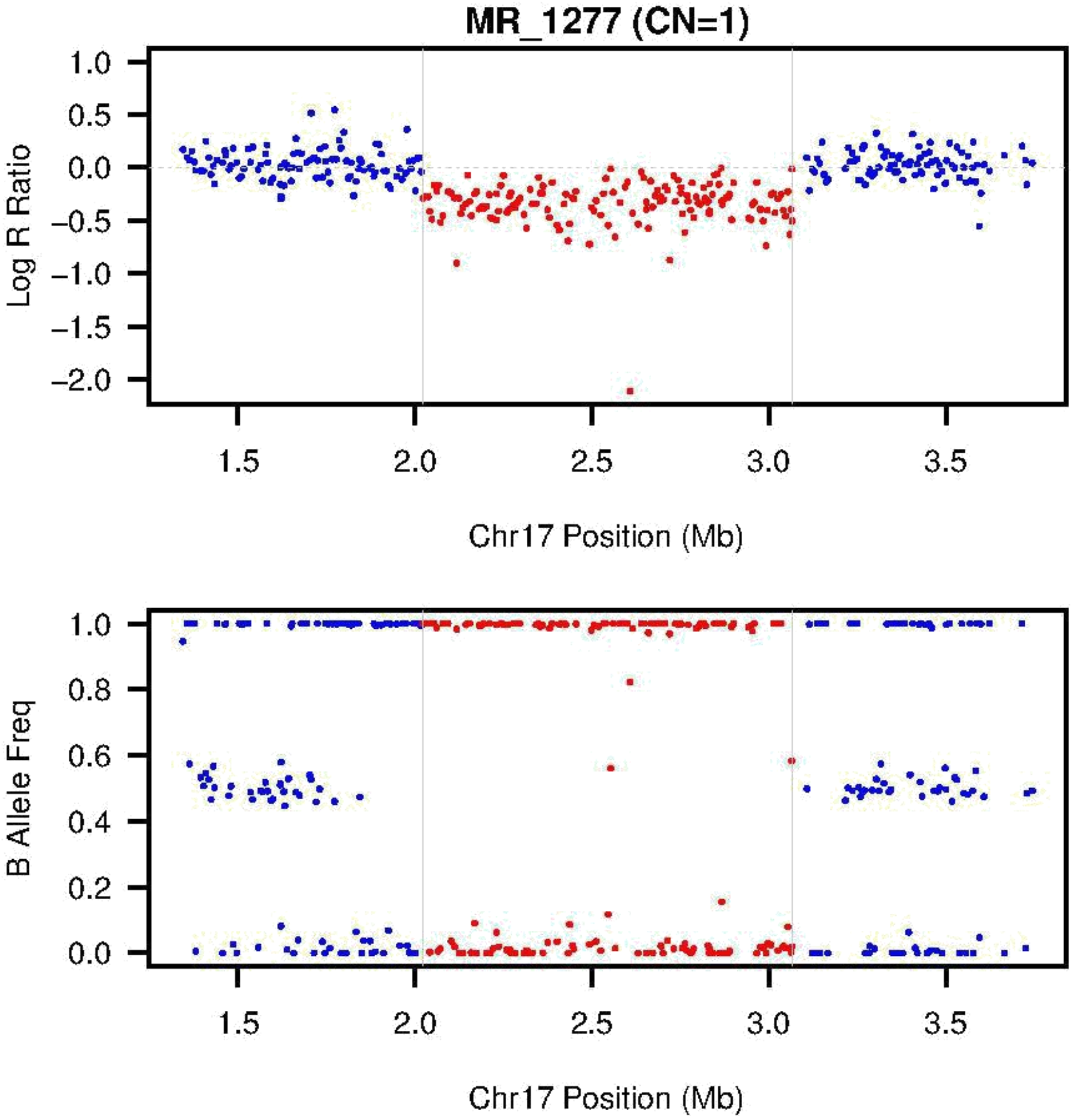

MR_1277: 17p13.3 (Miller-Dieker) Del

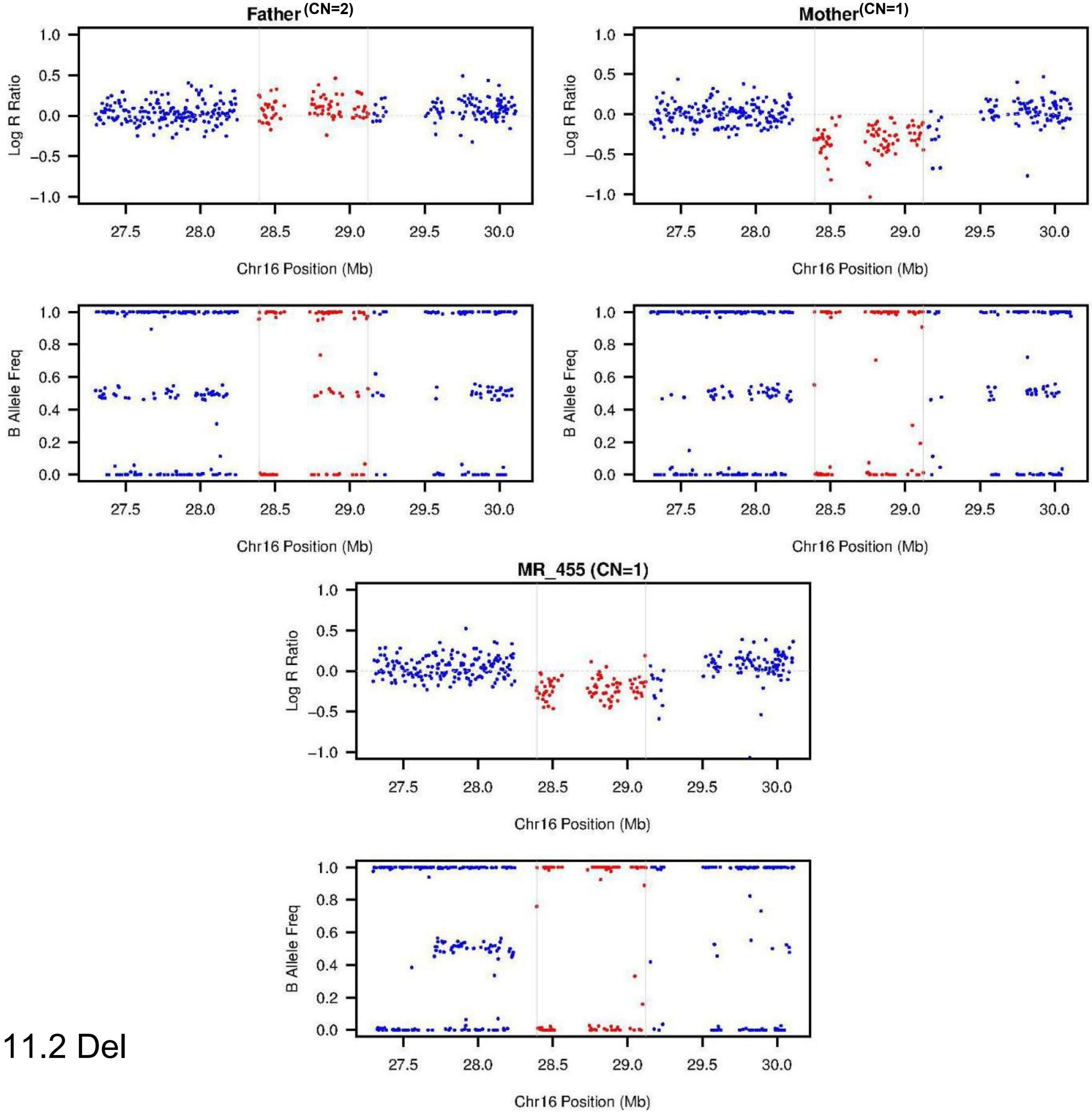

MR_455: 16p11.2 Del

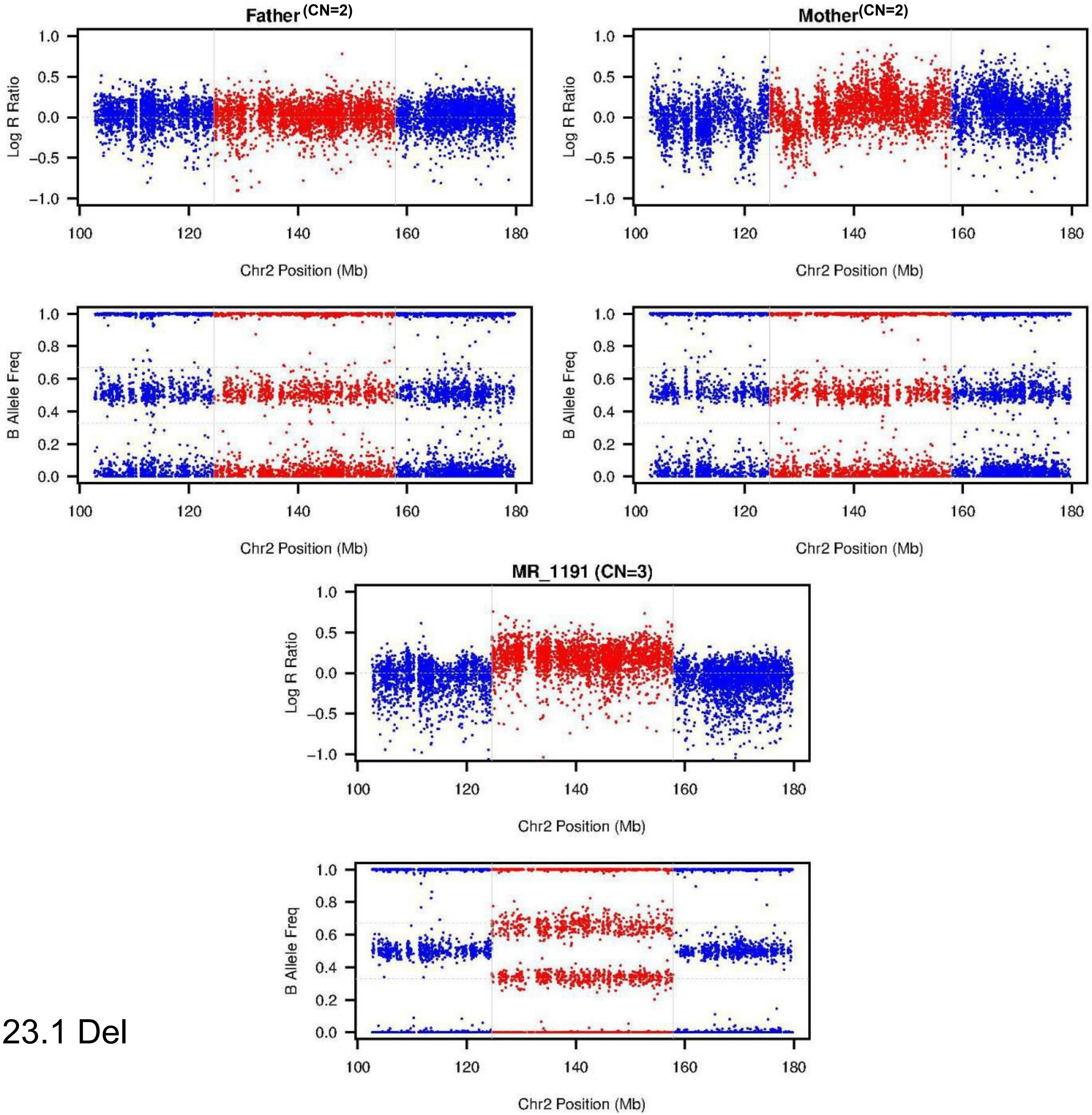

MR_1191: 2q23.1 Del

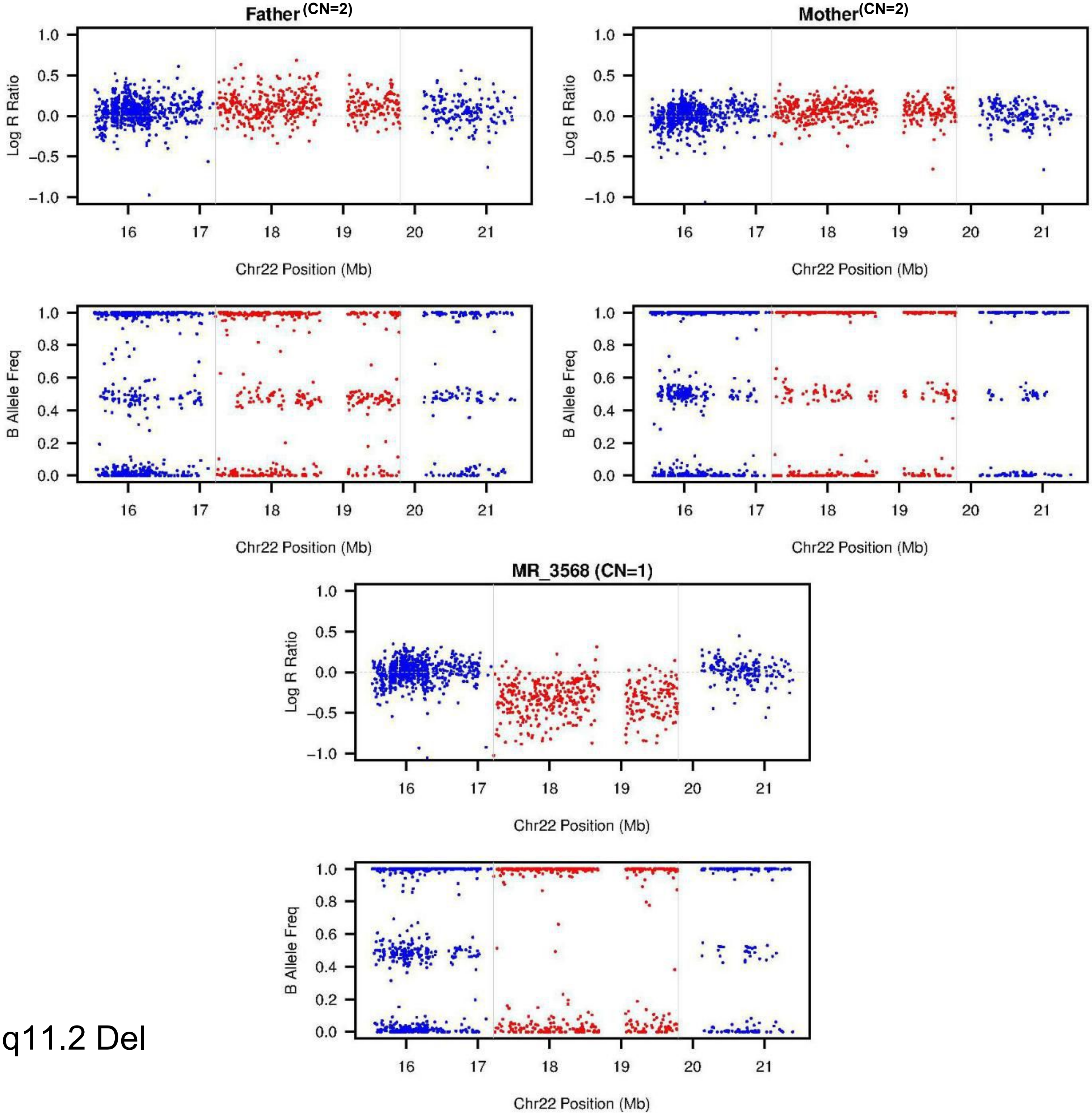

MR_3568: 22q11.2 Del

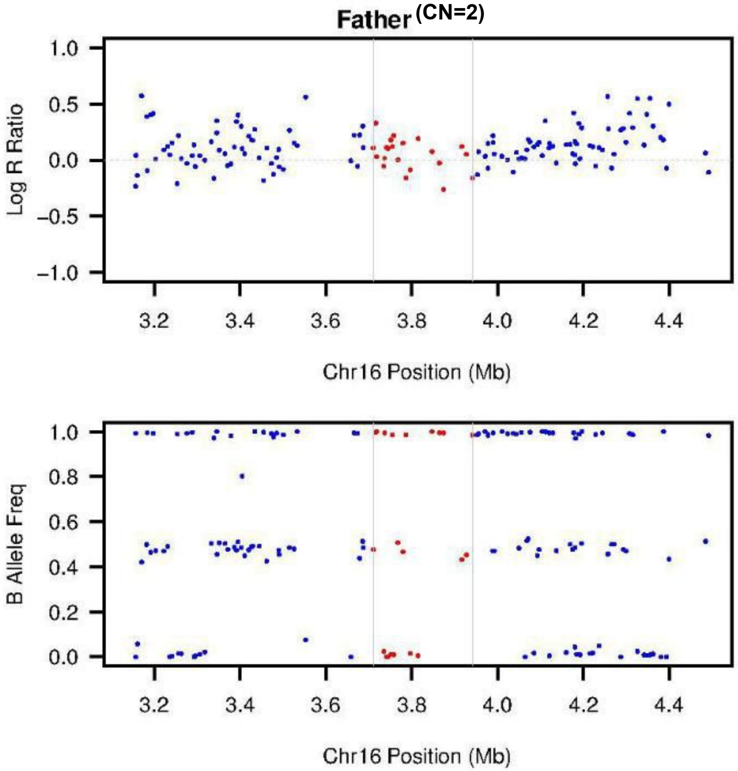

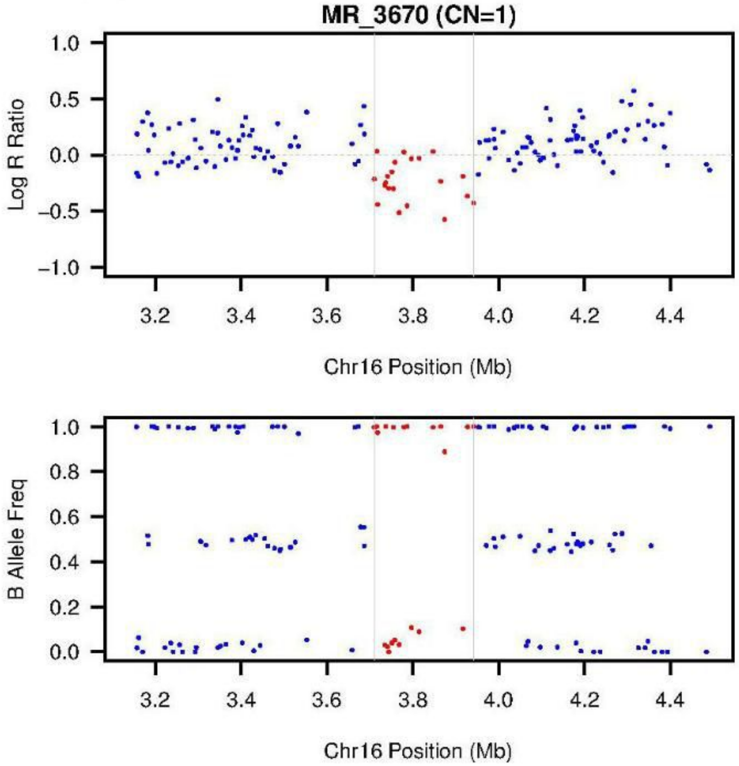

MR_3670: CREBBP Del

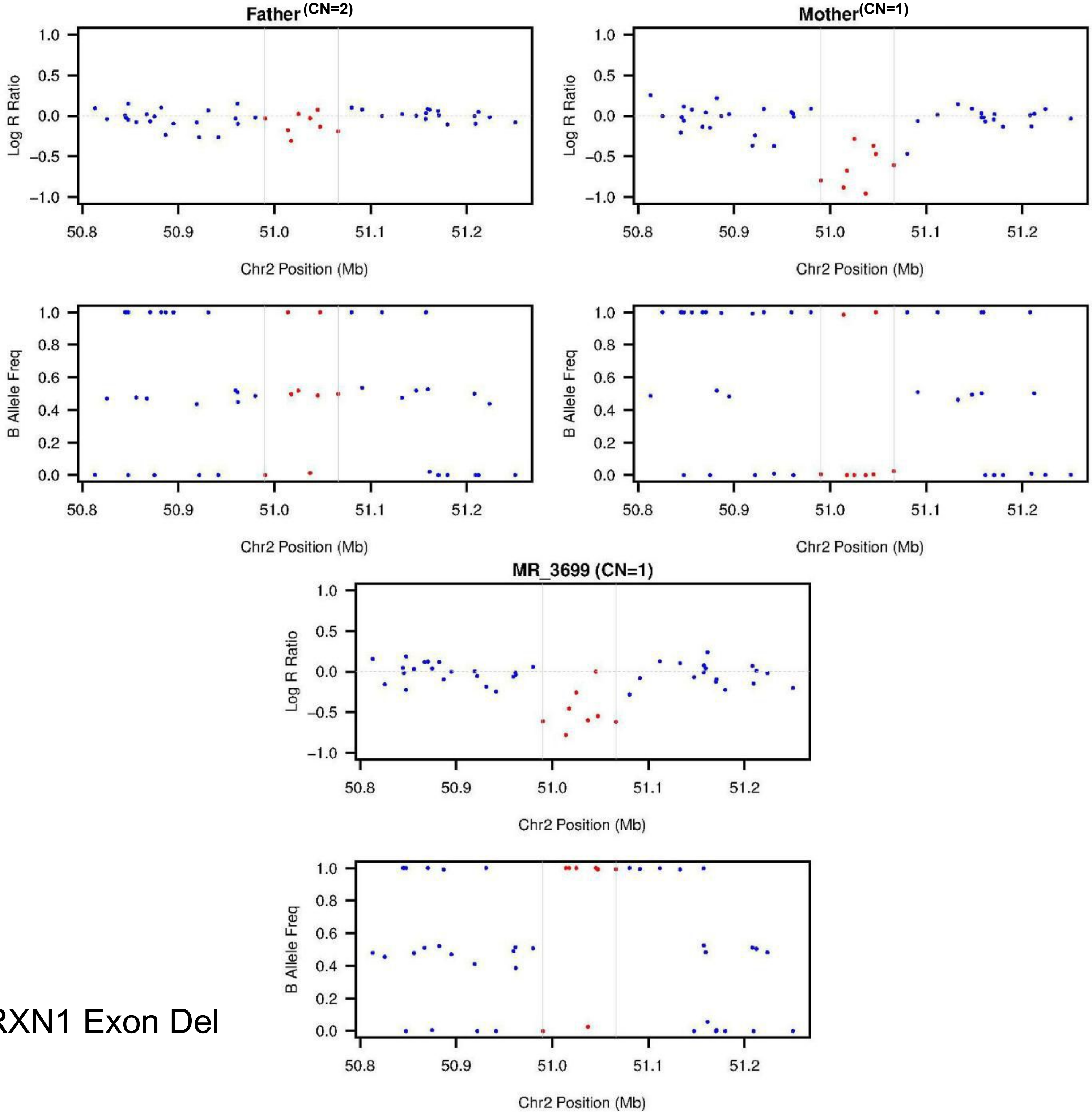

MR_3699: NRXN1 Exon Del

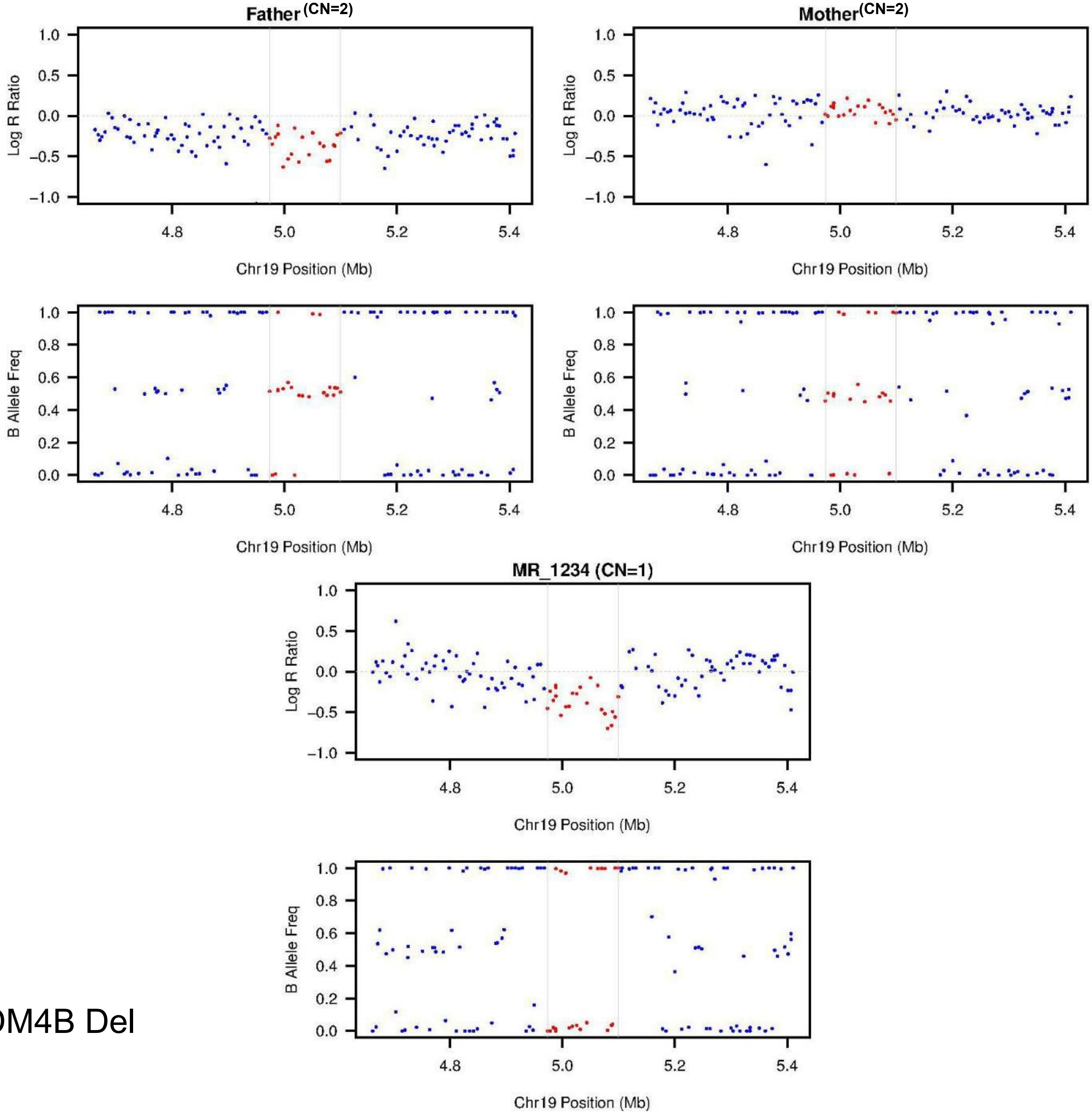

MR_1234: KDM4B Del

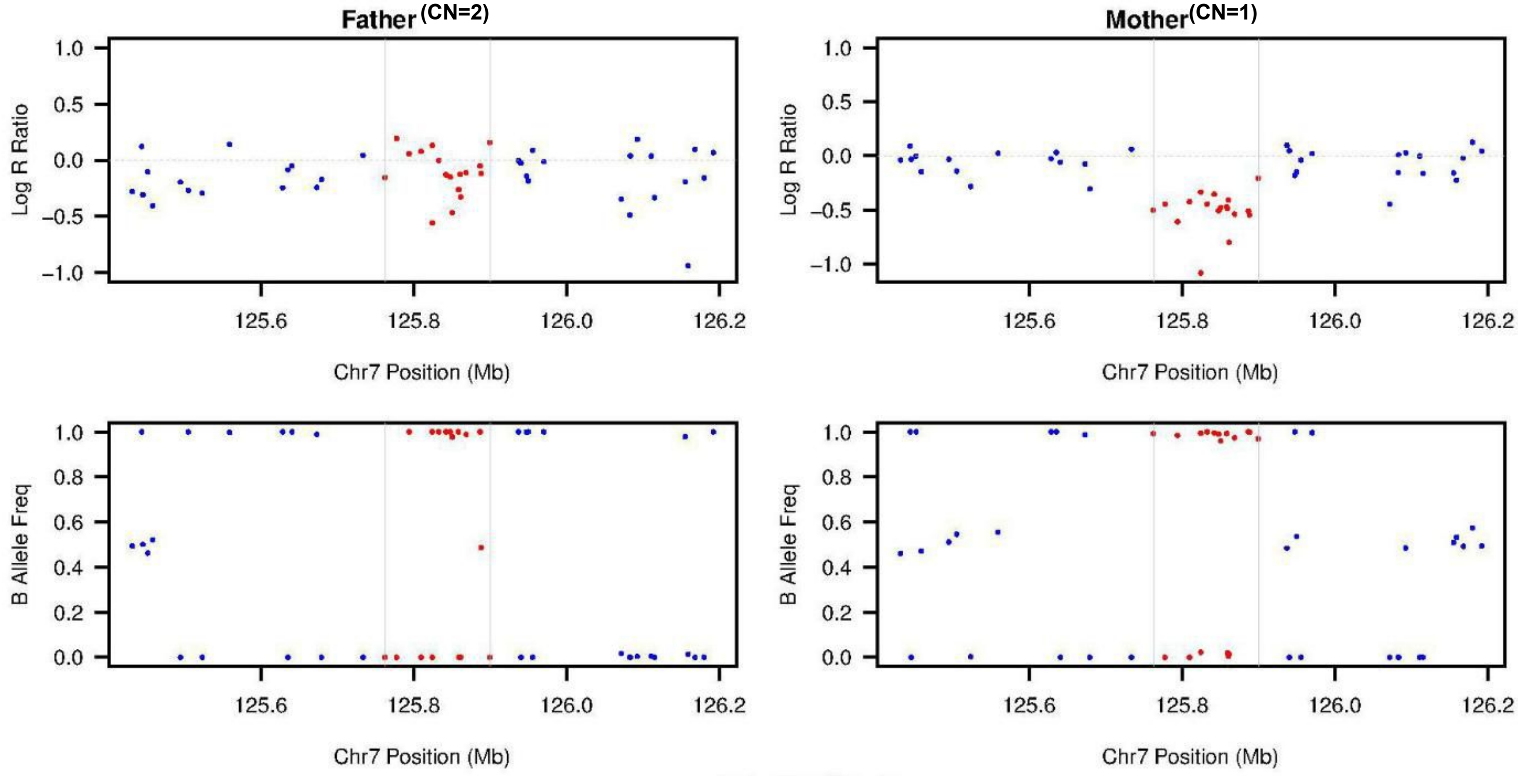

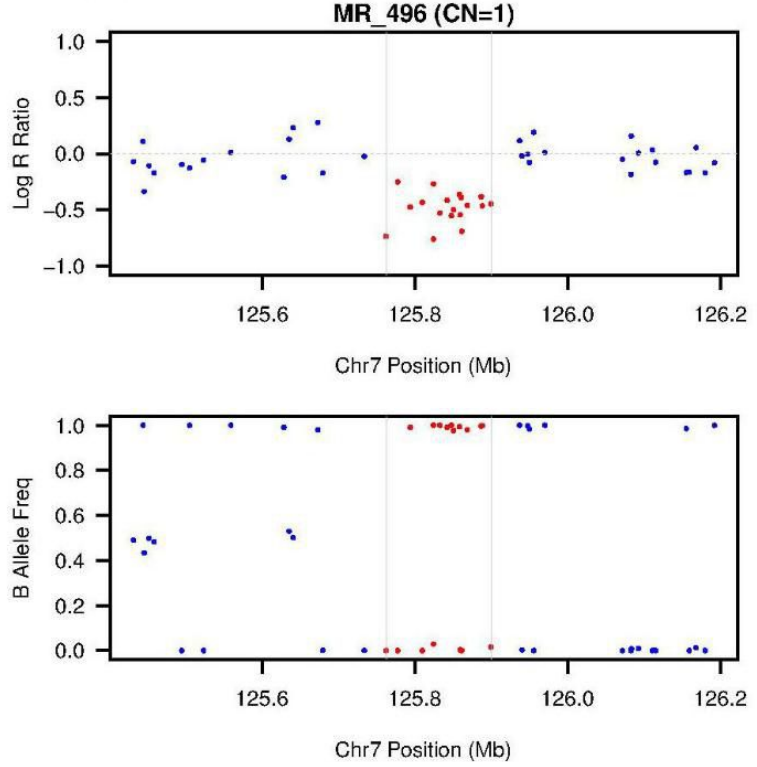

MR_496: GRM8 Exon Del

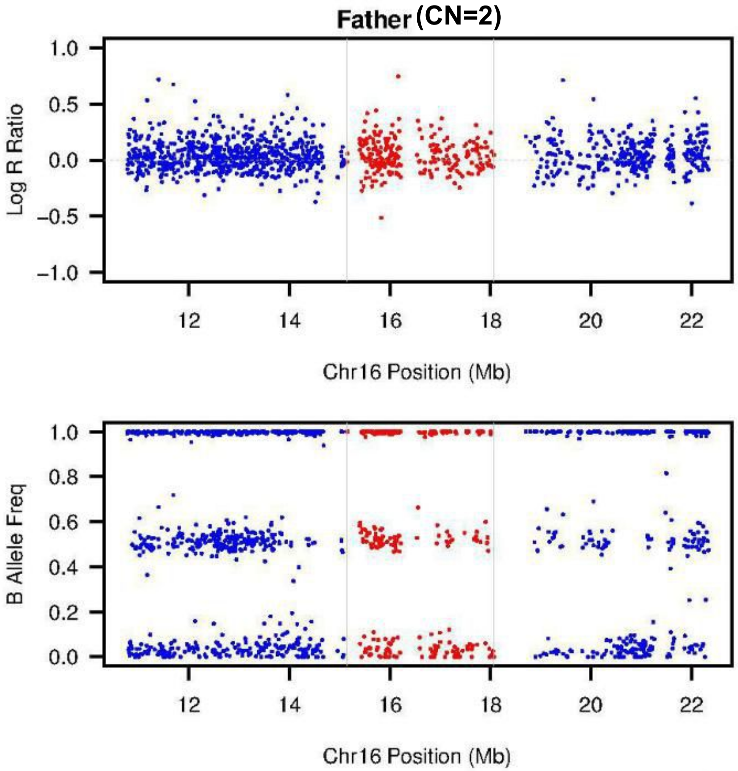

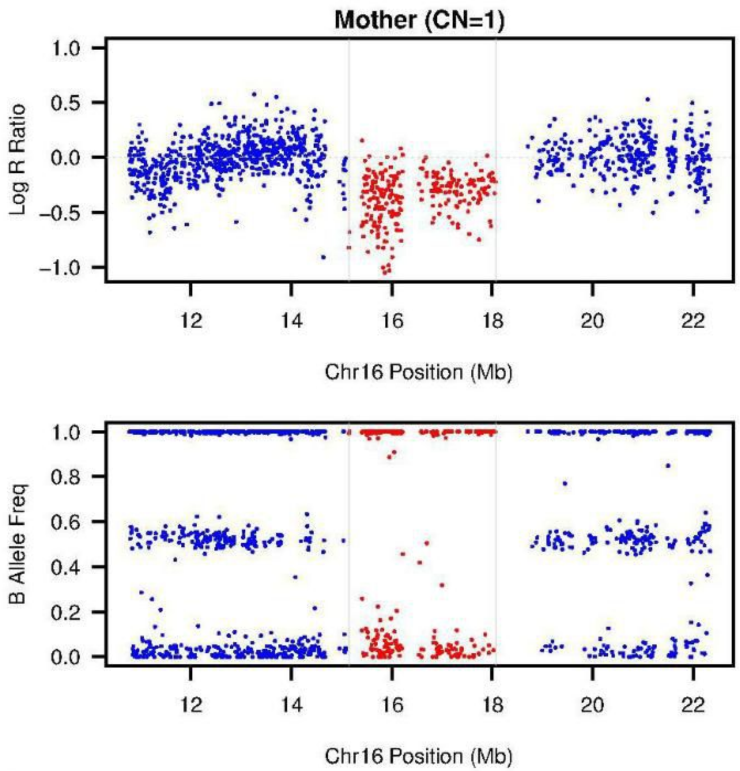

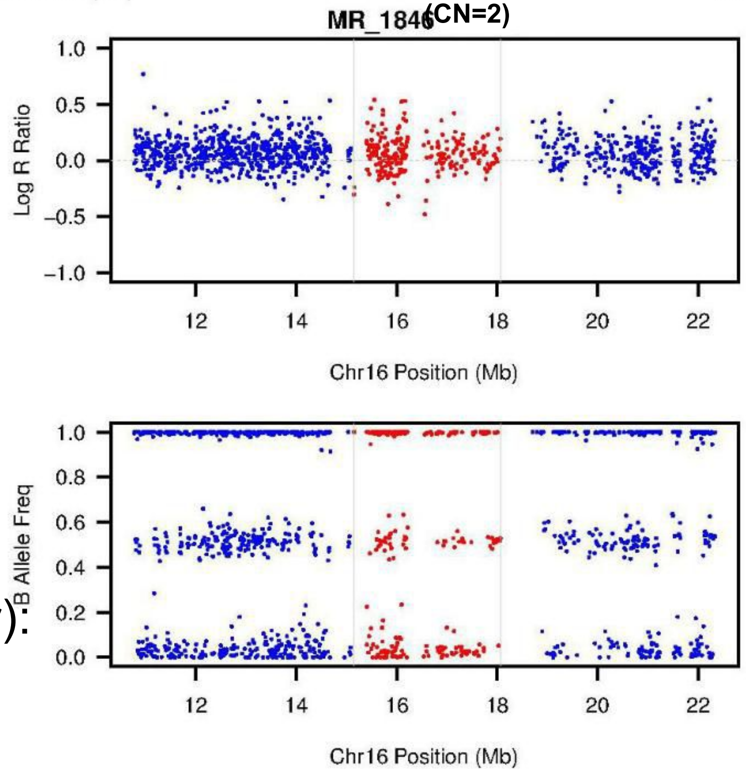

CTRL_1848 (MR_1846's mother): 16p13.3 Del

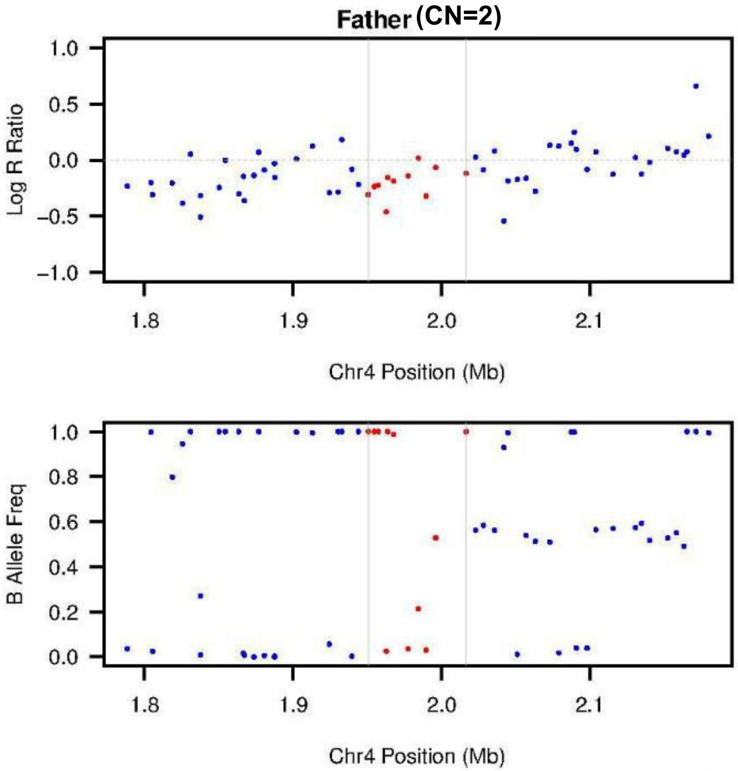

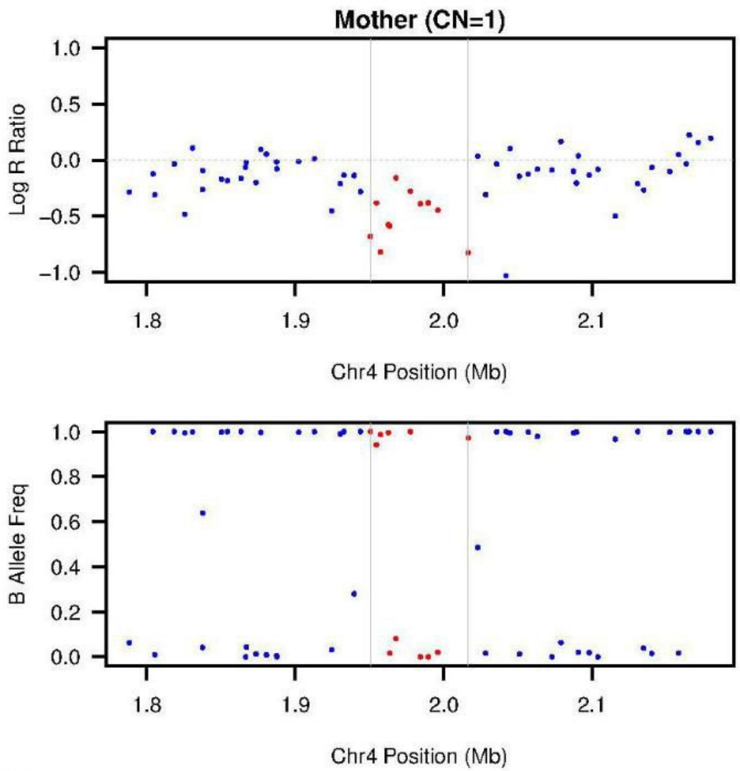

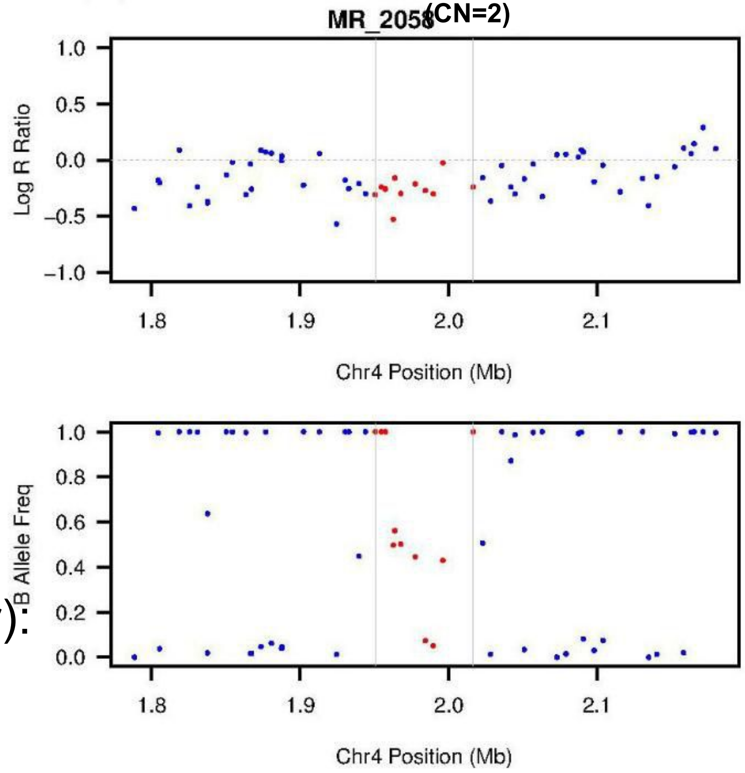

CTRL_2060 (MR_2058's mother):
WHSC1,2 Del

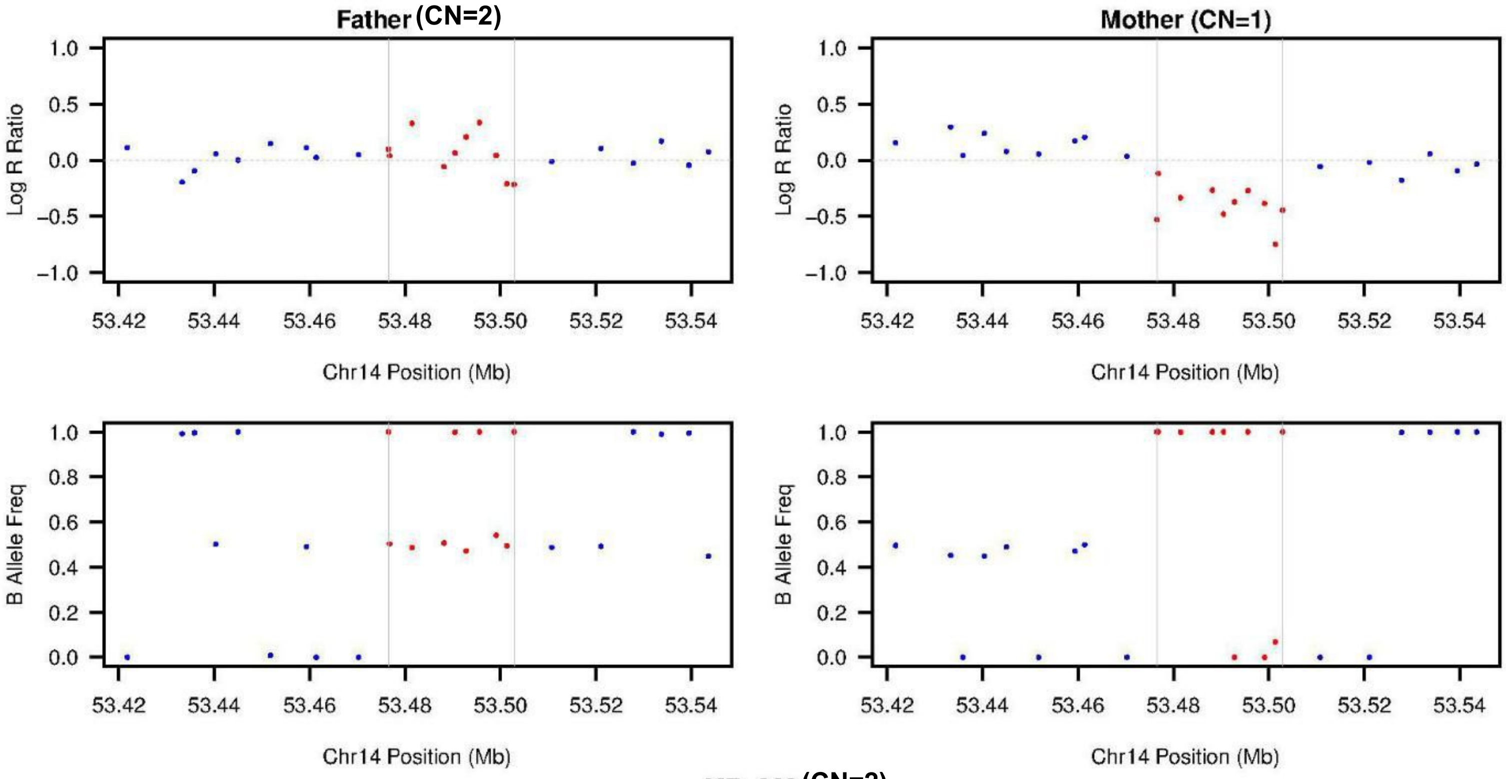

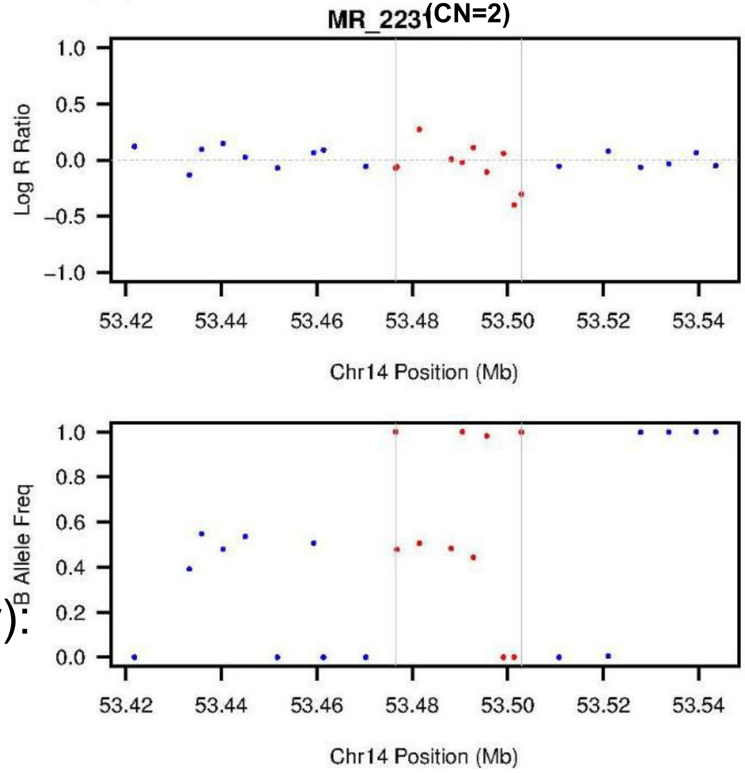

CTRL_2233 (MR_2231’s mother):
BMP4 Del

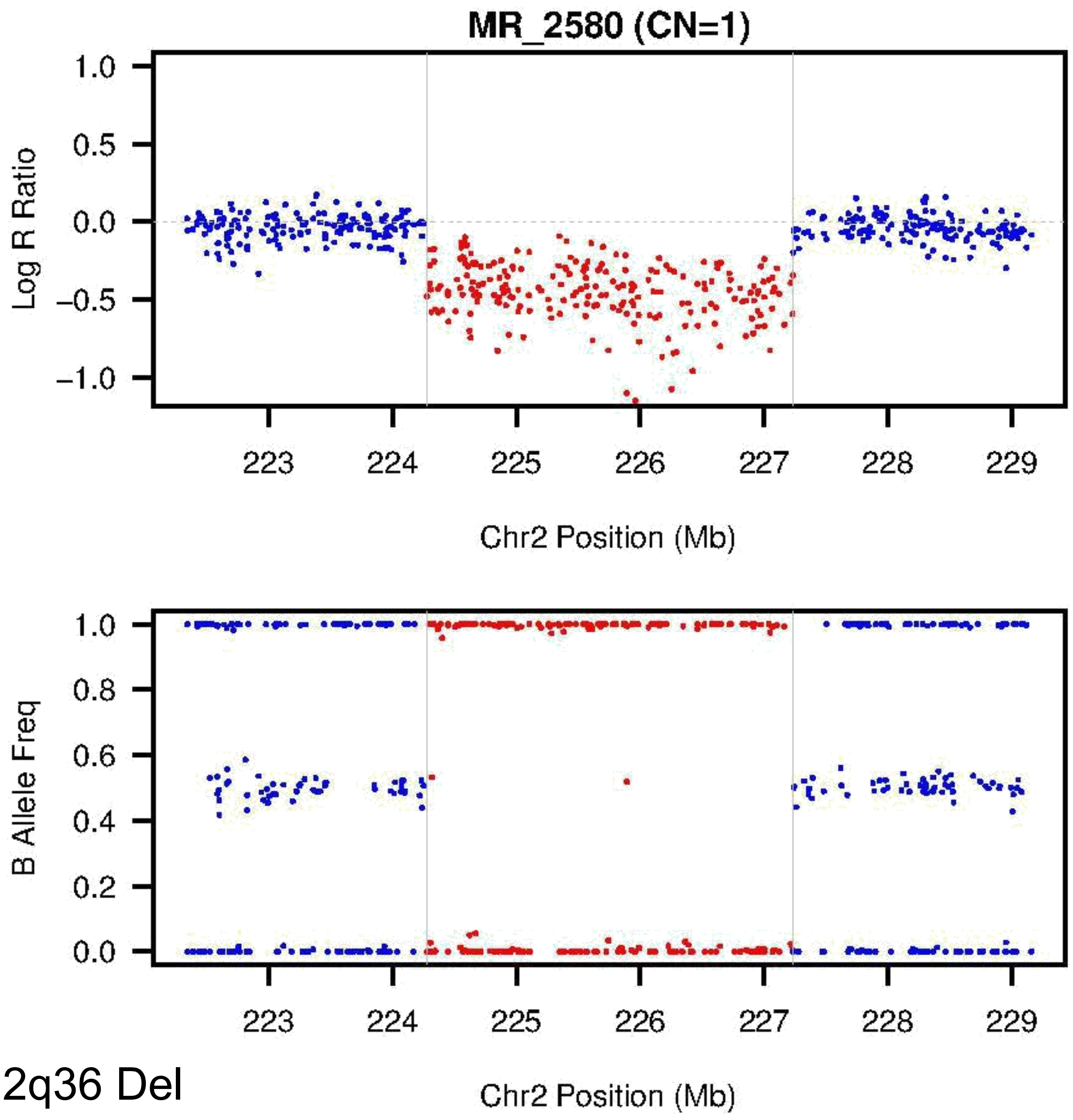

CTRL_2580: 2q36 Del

Figure S6 Cumulative proportion of *de novo* CNVs as a function of gene counts.

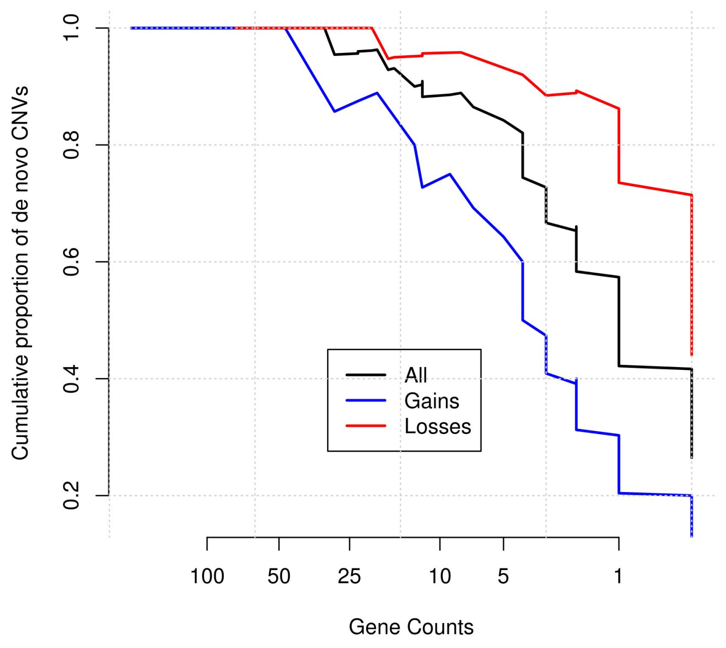

Gene counts are defined as the number of refSeq coding genes whose coding exon sequences overlap with the CNV segment.

Figure S7 Cumulative distribution of gene level statistics for all CNVs detected in population controls. (A) The number of refSeq coding genes whose coding exons overlap the CNV segment. (B) Haplo-insufficiency LOD scores (Huang et al. 2010) of all CNV segments.

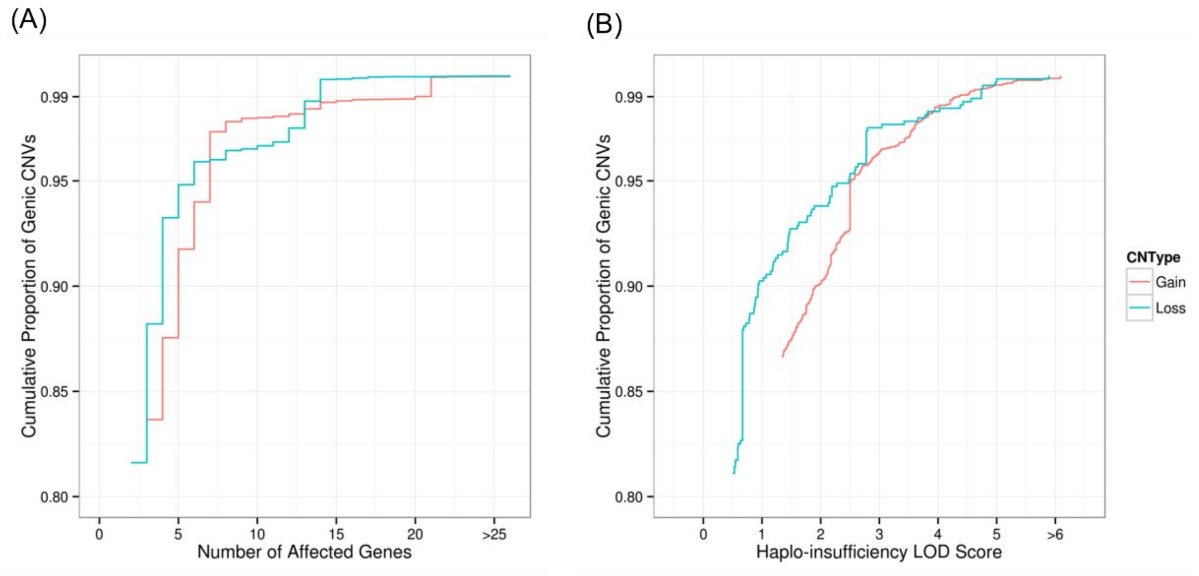

Figure S8 The case of mosaic uniparental disomy (UPD).

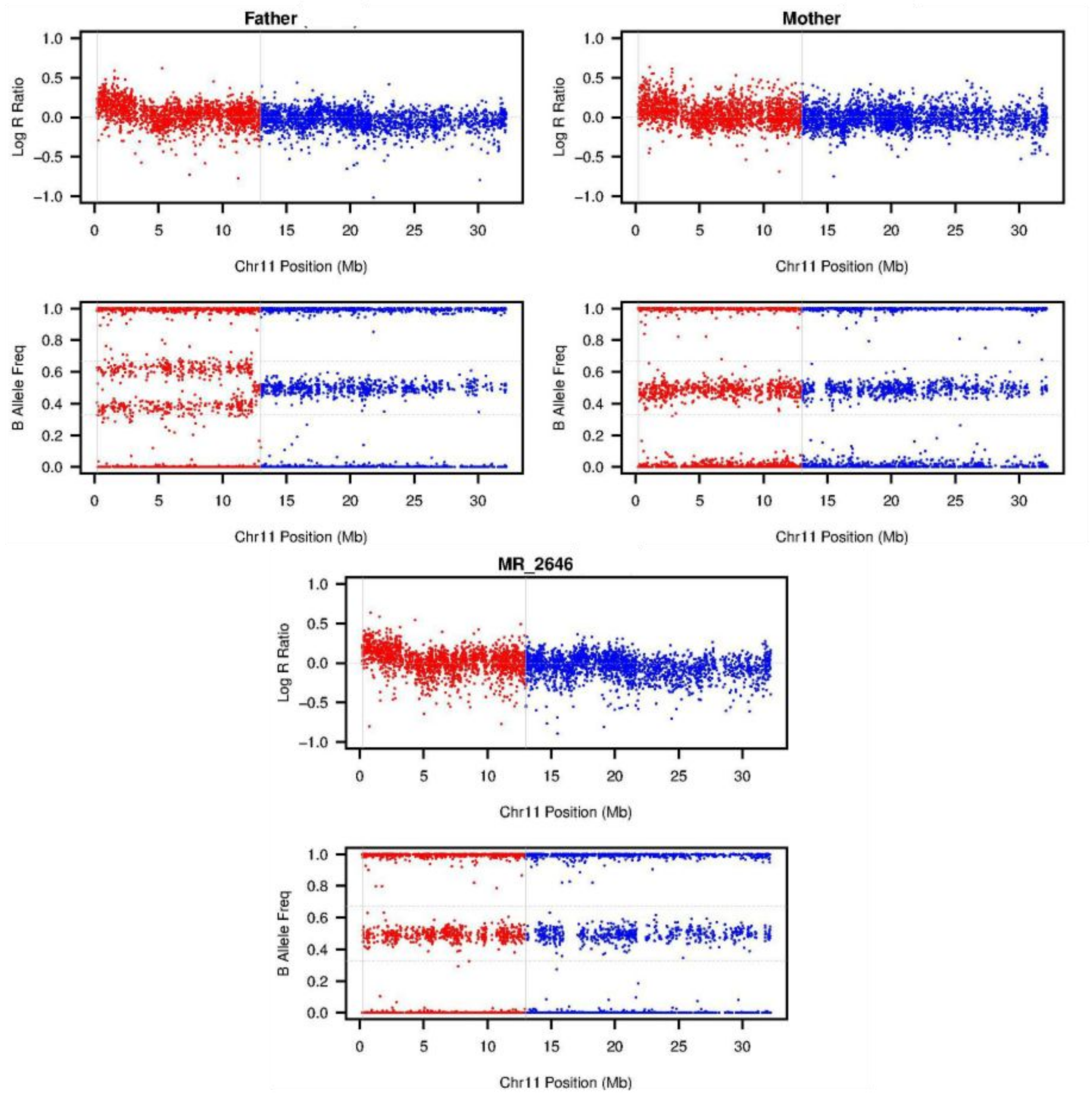

Segmental UPD of 11p15.3 to p-terminus was found in the DNA from MR_2646's father. The figure displays LRR and BAF signals of patient MR_2646 and her parents. All samples had normal LRR; father's BAF shows a split consistent with loss of heterozygosity in about 30% of cells. The elevation of LRR signals at the 11p terminus was likely caused by the assay artifacts.

Figure S9 The case of ultra-rare small exonic deletion identified from SCOUT analysis.

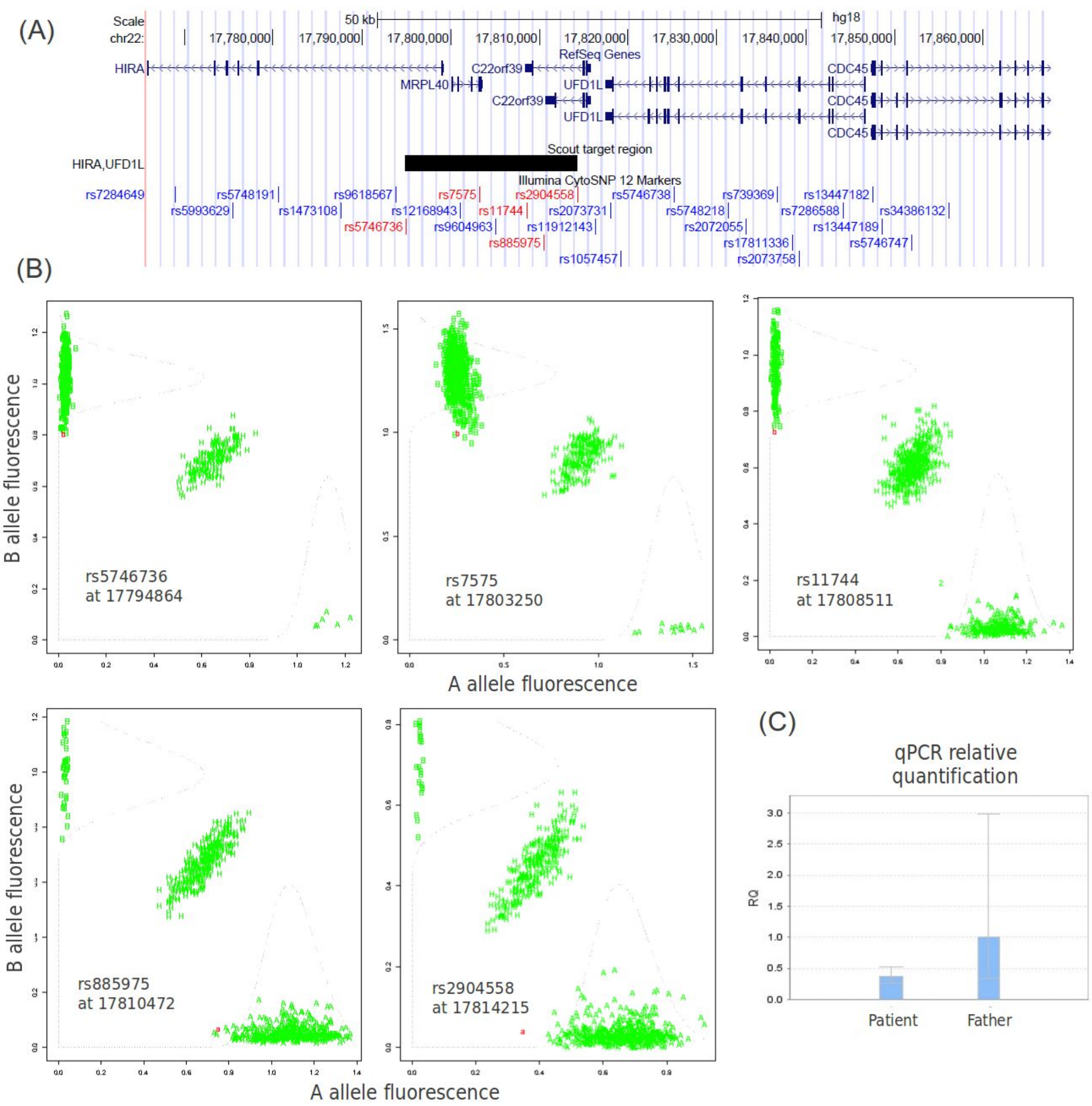

(A) Genome browser tracks show the genomic position of refSeq genes, targeted interval, all SNPs markers on CytoSNP12 array, and markers selected for rare CNV genotyping (red). (B) Allele-specific fluorescent intensities at the five selected markers. The sample carrying the deletion (MR_3590) is shown in red, and exhibited consistent weaker intensities. (C) qRT-PCR relative quantification of the first coding exon of *HIRA* gene. The deletion is not inherited from father (mother not available for testing).

Figure S10 Deletions at 15q11.2-13 locus identified in two patients with Angelman syndrome. Three loci, overlapping segmental duplications and devoid of SNP probes, are commonly referred to as BP1-BP3.

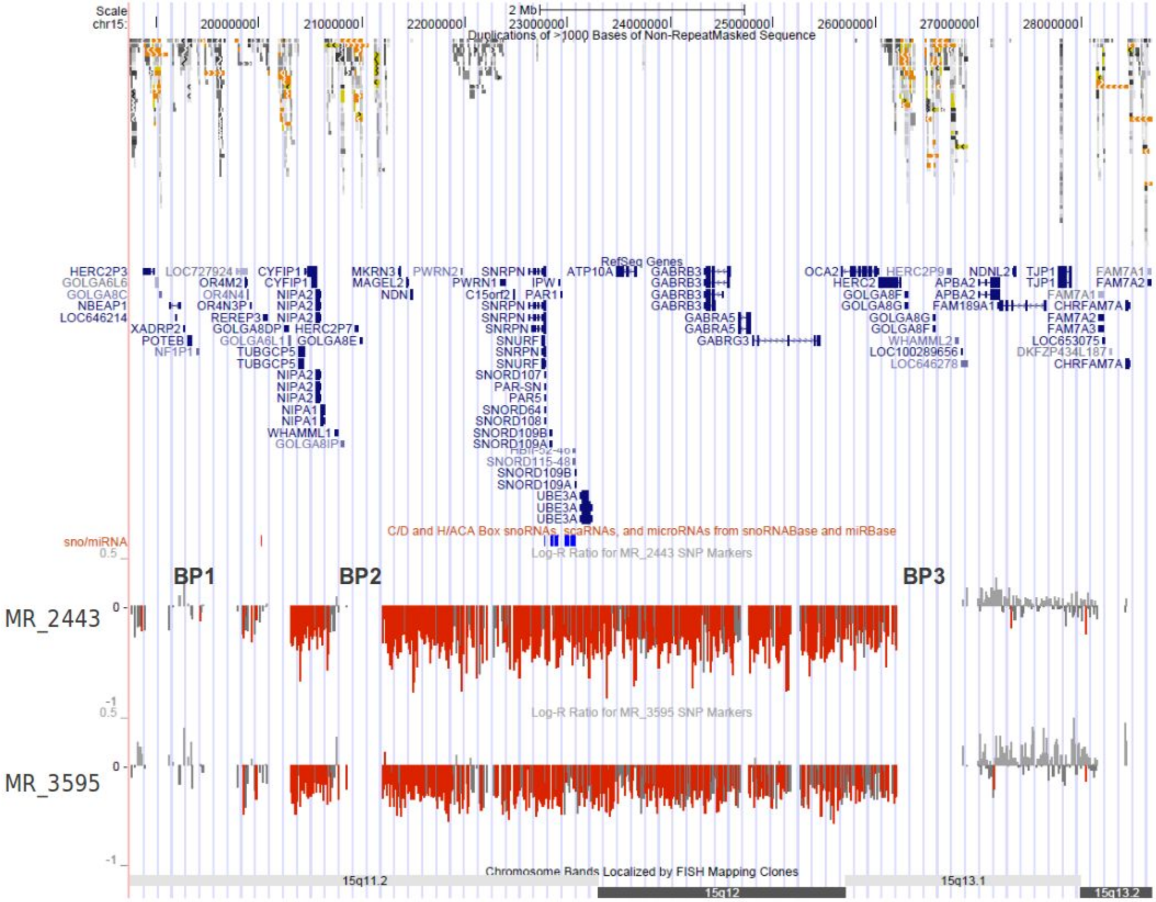

Figure S11 Overlapping ultra-rare CNVs in patients.

We imposed a minimal 5% reciprocal overlap to exclude cases of chance overlapping of several small CNVs with a very large one. Recurrent CNVs in known genomic disorders mediated by NAHR were also excluded. The following cases were identified: (A) Two large *de novo* pathogenic deletions intersect at 18q21 region, including *NEDD4L* gene. (B,C) Two cases of a small deletion embedded within a large pathogenic deletion. Genes disrupted by the small deletions, however, have not been implicated in MR. (D,E) Two cases of overlapping duplications whose common region duplicates a single gene. Both duplications have unknown clinical significance. (F,G) Two cases of small recurrent deletions with almost identical boundary. (F) Deletions shared by MR_557 and MR_144 omit exon 13 of *CTNNA3* gene. The same deletion also appeared in 3 unrelated controls; deletions that disrupt this gene are common in controls. (G) Deletions shared by MR_1234 and MR_1963 omit exon 8 of *ARSF* gene. No deletion is found to disrupt this gene in controls or in public databases.

Supplementary Figure S11

(A)

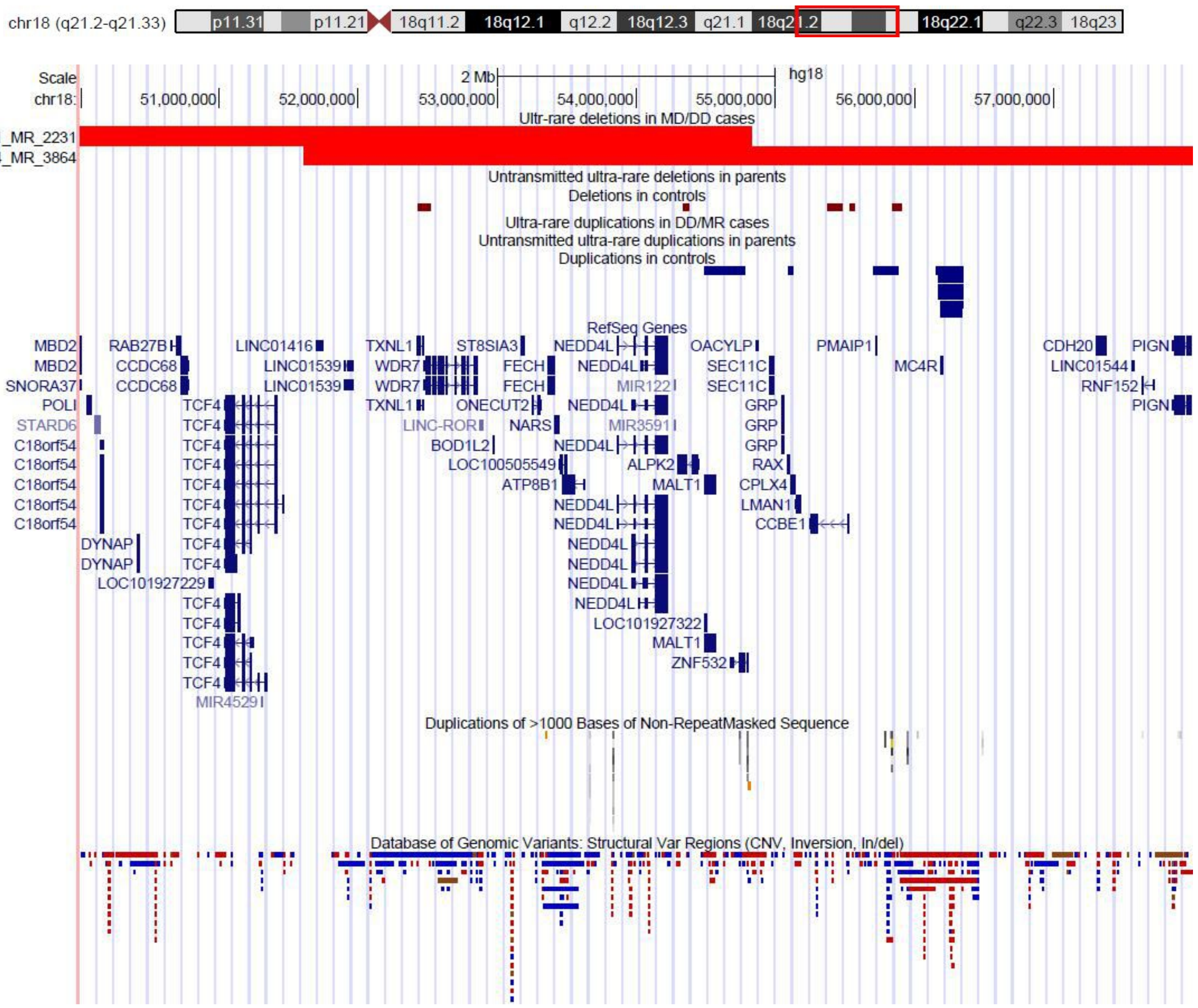

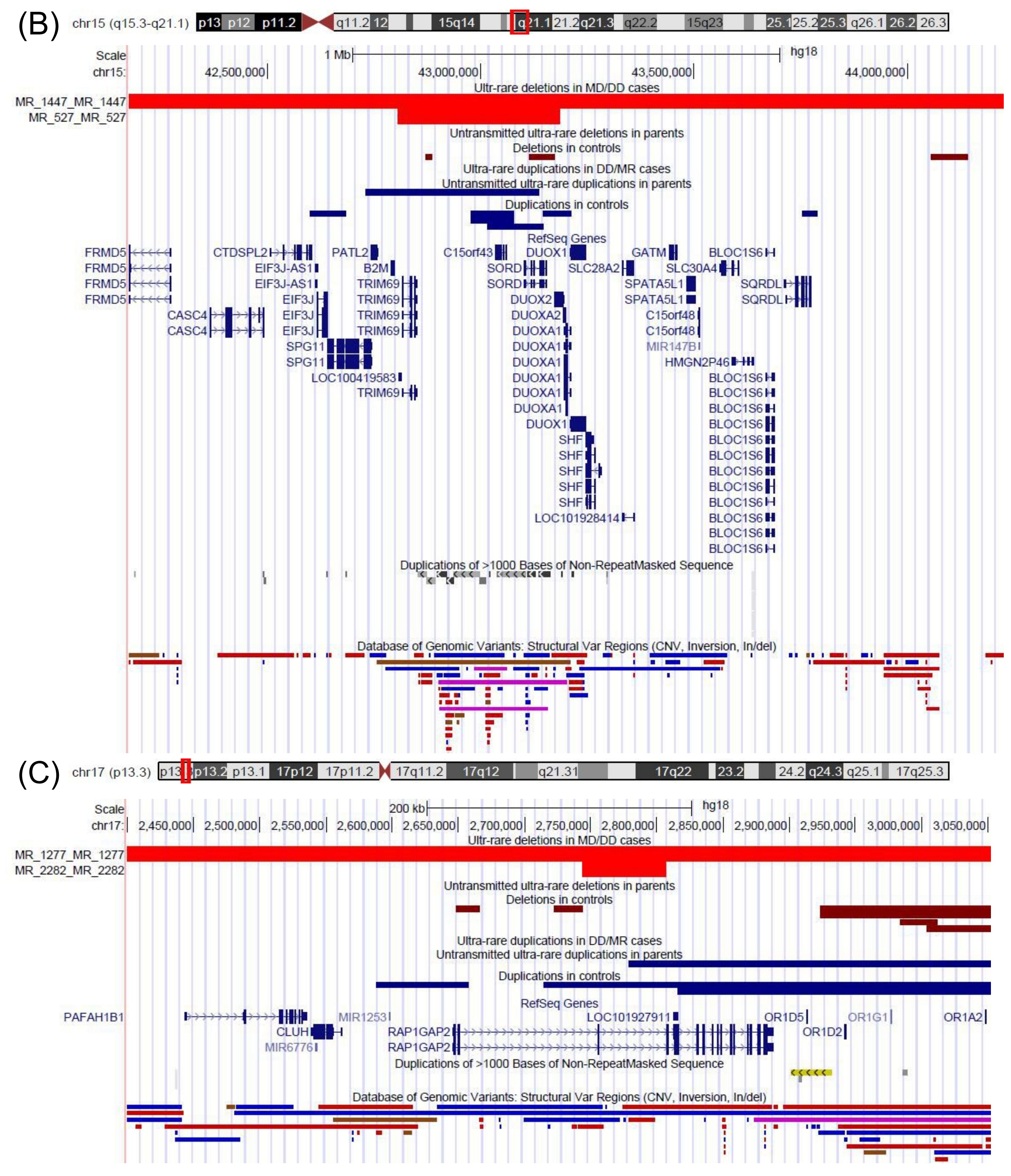

(B)
chr15 (q15.3-q21.1)
p13 p12 p11.2 q11.2 12 15q14 q21.1 21.2 q21.3 q22.2 15q23 25.1 25.2 25.3 q26.1 26.2 26.3
Scale 1 Mb hg18
chr15: 42,500,000 43,000,000 43,500,000 44,000,000
Ultr-rare deletions in MD/DD cases
MR_1447_MR_1447
MR_527_MR_527
Untransmitted ultra-rare deletions in parents
Deletions in controls
Ultra-rare duplications in DD/MR cases
Untransmitted ultra-rare duplications in parents
Duplications in controls
RefSeq Genes
FRMD5
CASC4
CTDSPL2
EIF3J-AS1
EIF3J
SPG11
LOC100419583
PATL2
B2M
TRIM69
C15orf43
SORD
DUOX1
DUOX2
DUOXA2
DUOXA1
SHF
LOC101928414
SLC28A2
GATM
SPATA5L1
C15orf48
MIR147B
SLC30A4
HMGN2P46
BLOC1S6
SQRDL
Duplications of >1000 Bases of Non-RepeatMasked Sequence
Database of Genomic Variants: Structural Var Regions (CNV, Inversion, In/del)
(C)
chr17 (p13.3)
p13 p13.2 p13.1 17p12 17p11.2 17q11.2 17q12 q21.31 17q22 23.2 24.2 q24.3 q25.1 17q25.3
Scale 200 kb hg18
chr17: 2,450,000 2,500,000 2,550,000 2,600,000 2,650,000 2,700,000 2,750,000 2,800,000 2,850,000 2,900,000 2,950,000 3,000,000 3,050,000
Ultr-rare deletions in MD/DD cases
MR_1277_MR_1277
MR_2282_MR_2282
Untransmitted ultra-rare deletions in parents
Deletions in controls
Ultra-rare duplications in DD/MR cases
Untransmitted ultra-rare duplications in parents
Duplications in controls
RefSeq Genes
PAFAH1B1
CLUH
MIR6776
MIR1253
RAP1GAP2
LOC101927911
OR1D5
OR1D2
OR1G1
OR1A2
Duplications of >1000 Bases of Non-RepeatMasked Sequence
Database of Genomic Variants: Structural Var Regions (CNV, Inversion, In/del)

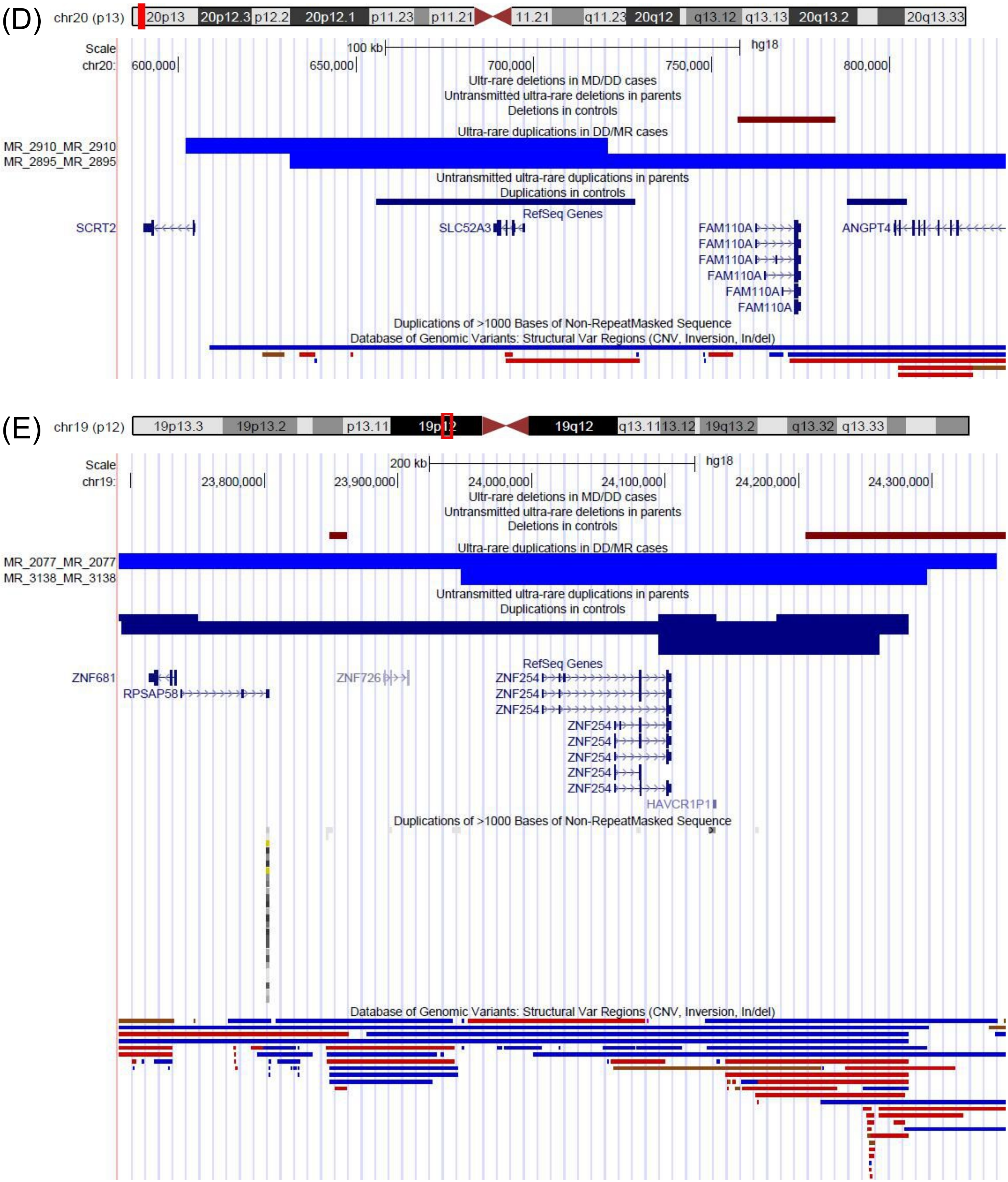
(D)
chr20 (p13)
20p13
20p12.3
p12.2
20p12.1
p11.23
p11.21
11.21
q11.23
20q12
q13.12
q13.13
20q13.2
20q13.33
Scale
100 kb
hg18
chr20:
600,000
650,000
700,000
750,000
800,000
Ultr-rare deletions in MD/DD cases
Untransmitted ultra-rare deletions in parents
Deletions in controls
Ultra-rare duplications in DD/MR cases
MR_2910_MR_2910
MR_2895_MR_2895
Untransmitted ultra-rare duplications in parents
Duplications in controls
RefSeq Genes
SCRT2
SLC52A3
FAM110A
FAM110A
FAM110A
FAM110A
FAM110A
FAM110A
ANGPT4
Duplications of >1000 Bases of Non-RepeatMasked Sequence
Database of Genomic Variants: Structural Var Regions (CNV, Inversion, In/del)
(E)
chr19 (p12)
19p13.3
19p13.2
p13.11
19p12
19q12
q13.11
13.12
19q13.2
q13.32
q13.33
Scale
200 kb
hg18
chr19:
23,800,000
23,900,000
24,000,000
24,100,000
24,200,000
24,300,000
Ultr-rare deletions in MD/DD cases
Untransmitted ultra-rare deletions in parents
Deletions in controls
Ultra-rare duplications in DD/MR cases
MR_2077_MR_2077
MR_3138_MR_3138
Untransmitted ultra-rare duplications in parents
Duplications in controls
RefSeq Genes
ZNF681
RPSAP58
ZNF726
ZNF254
ZNF254
ZNF254
ZNF254
ZNF254
ZNF254
ZNF254
ZNF254
HAVCR1P1
Duplications of >1000 Bases of Non-RepeatMasked Sequence
Database of Genomic Variants: Structural Var Regions (CNV, Inversion, In/del)

(F)

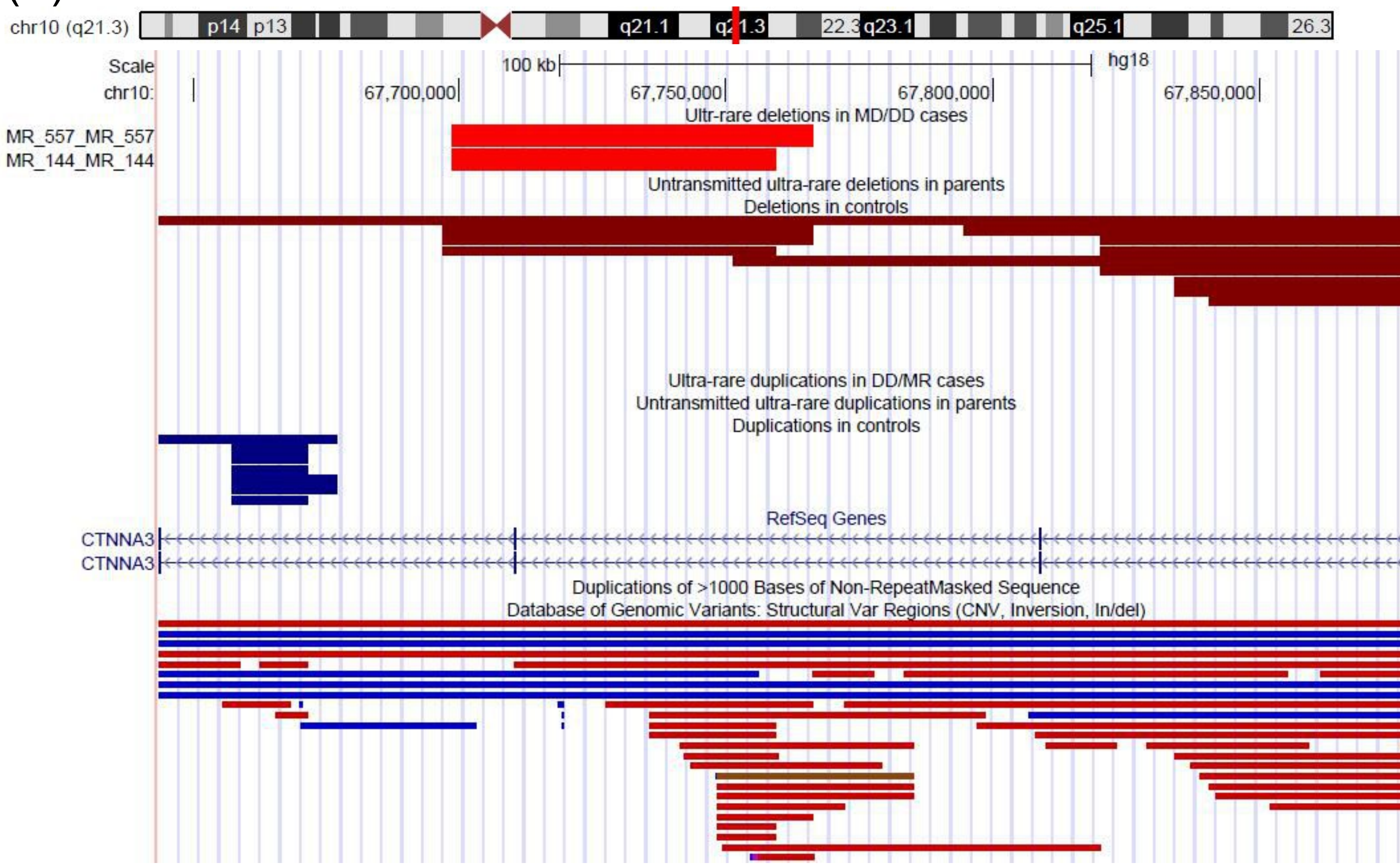
chr10 (q21.3)
p14 p13 q21.1 q21.3 22.3 q23.1 q25.1 26.3
Scale 100 kb hg18
chr10: 67,700,000 67,750,000 67,800,000 67,850,000
Ultr-rare deletions in MD/DD cases
MR_557_MR_557
MR_144_MR_144
Untransmitted ultra-rare deletions in parents
Deletions in controls
Ultra-rare duplications in DD/MR cases
Untransmitted ultra-rare duplications in parents
Duplications in controls
RefSeq Genes
CTNNA3
CTNNA3
Duplications of >1000 Bases of Non-RepeatMasked Sequence
Database of Genomic Variants: Structural Var Regions (CNV, Inversion, In/del)

(G)

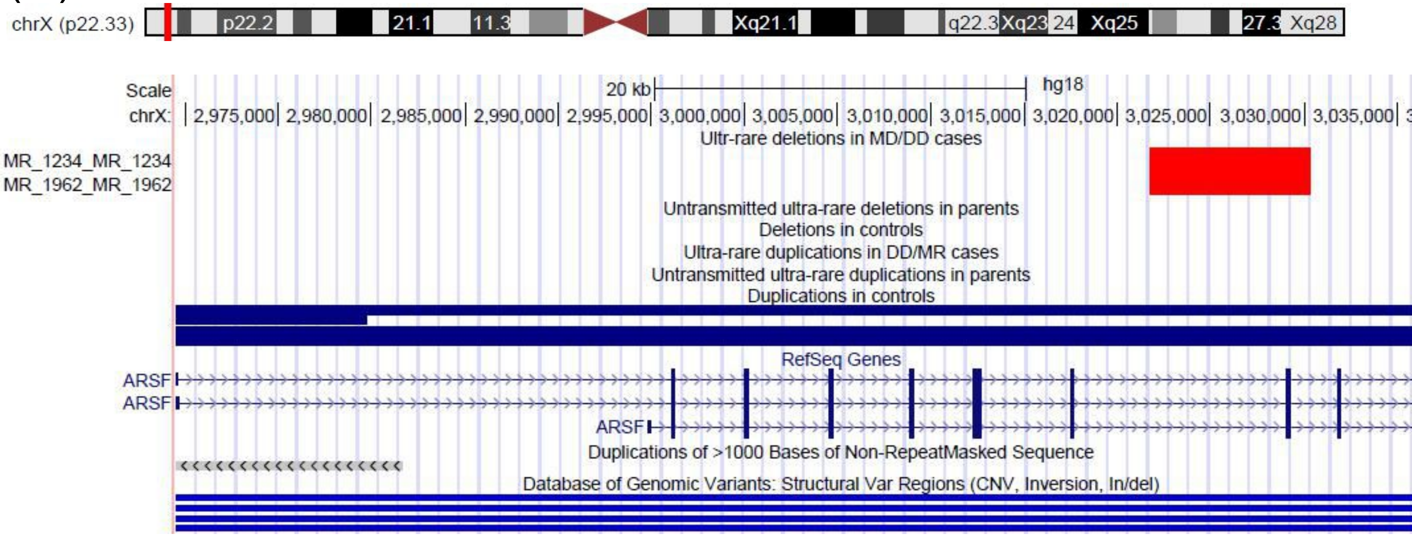
chrX (p22.33)
p22.2 21.1 11.3 Xq21.1 q22.3 Xq23 24 Xq25 27.3 Xq28
Scale 20 kb hg18
chrX: 2,975,000 2,980,000 2,985,000 2,990,000 2,995,000 3,000,000 3,005,000 3,010,000 3,015,000 3,020,000 3,025,000 3,030,000 3,035,000
Ultr-rare deletions in MD/DD cases
MR_1234_MR_1234
MR_1962_MR_1962
Untransmitted ultra-rare deletions in parents
Deletions in controls
Ultra-rare duplications in DD/MR cases
Untransmitted ultra-rare duplications in parents
Duplications in controls
RefSeq Genes
ARSF
ARSF
ARSF
Duplications of >1000 Bases of Non-RepeatMasked Sequence
Database of Genomic Variants: Structural Var Regions (CNV, Inversion, In/del)

Supplementary References

Bailey, J. A., J. M. Kidd, and E. E. Eichler. 2008. "Human copy number polymorphic genes." *Cytogenet Genome Res* 123 (1-4):234-43. doi: 10.1159/000184713.

Conrad, D. F., D. Pinto, R. Redon, L. Feuk, O. Gokcumen, Y. Zhang, . . . M. E. Hurles. 2010. "Origins and functional impact of copy number variation in the human genome." *Nature* 464 (7289):704-12. doi: 10.1038/nature08516.

Diskin, S. J., M. Li, C. Hou, S. Yang, J. Glessner, H. Hakonarson, . . . K. Wang. 2008. "Adjustment of genomic waves in signal intensities from whole-genome SNP genotyping platforms." *Nucleic Acids Res* 36 (19):e126. doi: 10.1093/nar/gkn556.

Gonzalez, J. R., B. Rodriguez-Santiago, A. Caceres, R. Pique-Regi, N. Rothman, S. J. Chanock, . . . L. A. Perez-Jurado. 2011. "A fast and accurate method to detect allelic genomic imbalances underlying mosaic rearrangements using SNP array data." *BMC Bioinformatics* 12:166. doi: 10.1186/1471-2105-12-166.

Huang, N., I. Lee, E. M. Marcotte, and M. E. Hurles. 2010. "Characterising and predicting haploinsufficiency in the human genome." *PLoS Genet* 6 (10):e1001154. doi: 10.1371/journal.pgen.1001154.

International HapMap, Consortium, D. M. Altshuler, R. A. Gibbs, L. Peltonen, D. M. Altshuler, R. A. Gibbs, . . . J. E. McEwen. 2010. "Integrating common and rare genetic variation in diverse human populations." *Nature* 467 (7311):52-8. doi: 10.1038/nature09298.

Itsara, A., G. M. Cooper, C. Baker, S. Girirajan, J. Li, D. Absher, . . . E. E. Eichler. 2009. "Population analysis of large copy number variants and hotspots of human genetic disease." *Am J Hum Genet* 84 (2):148-61. doi: 10.1016/j.ajhg.2008.12.014.

McCarroll, S. A., F. G. Kuruvilla, J. M. Korn, S. Cawley, J. Nemesh, A. Wysoker, . . . D. Altshuler. 2008. "Integrated detection and population-genetic analysis of SNPs and copy number variation." *Nat Genet* 40 (10):1166-74. doi: 10.1038/ng.238.

Mefford, H. C., G. M. Cooper, T. Zerr, J. D. Smith, C. Baker, N. Shafer, . . . E. E. Eichler. 2009. "A method for rapid, targeted CNV genotyping identifies rare variants associated with neurocognitive disease." *Genome Res* 19 (9):1579-85. doi: 10.1101/gr.094987.109.

Myers, S., L. Bottolo, C. Freeman, G. McVean, and P. Donnelly. 2005. "A fine-scale map of recombination rates and hotspots across the human genome." *Science* 310 (5746):321-4. doi: 10.1126/science.1117196.

Need, A. C., D. Ge, M. E. Weale, J. Maia, S. Feng, E. L. Heinzen, . . . D. B. Goldstein. 2009. "A genome-wide investigation of SNPs and CNVs in schizophrenia." *PLoS Genet* 5 (2):e1000373. doi: 10.1371/journal.pgen.1000373.

Park, H., J. I. Kim, Y. S. Ju, O. Gokcumen, R. E. Mills, S. Kim, . . . J. S. Seo. 2010. "Discovery of common Asian copy number variants using integrated high-resolution array CGH and massively parallel DNA sequencing." *Nat Genet* 42 (5):400-5. doi: 10.1038/ng.555.

Patterson, N., A. L. Price, and D. Reich. 2006. "Population structure and eigenanalysis." *PLoS Genet* 2 (12):e190. doi: 10.1371/journal.pgen.0020190.

Purcell, S., B. Neale, K. Todd-Brown, L. Thomas, M. A. Ferreira, D. Bender, . . . P. C. Sham. 2007. "PLINK: a tool set for whole-genome association and population-based linkage analyses." *Am J Hum Genet* 81 (3):559-75. doi: 10.1086/519795.

Sanders, S. J., A. G. Ercan-Sencicek, V. Hus, R. Luo, M. T. Murtha, D. Moreno-De-Luca, . . . M. W. State. 2011. "Multiple recurrent de novo CNVs, including duplications of the 7q11.23 Williams syndrome region, are strongly associated with autism." *Neuron* 70 (5):863-85. doi: 10.1016/j.neuron.2011.05.002.

Shaikh, T. H., X. Gai, J. C. Perin, J. T. Glessner, H. Xie, K. Murphy, . . . H. Hakonarson. 2009. "High-resolution mapping and analysis of copy number variations in the human genome: a

data resource for clinical and research applications." *Genome Res* 19 (9):1682-90. doi: 10.1101/gr.083501.108.

Uddin, M., B. Thiruvahindrapuram, S. Walker, Z. Wang, P. Hu, S. Lamoureux, . . . S. W. Scherer. 2014. "A high-resolution copy-number variation resource for clinical and population genetics." *Genet Med*. doi: 10.1038/gim.2014.178.

Wang, K., M. Li, D. Hadley, R. Liu, J. Glessner, S. F. Grant, . . . M. Bucan. 2007. "PennCNV: an integrated hidden Markov model designed for high-resolution copy number variation detection in whole-genome SNP genotyping data." *Genome Res* 17 (11):1665-74. doi: 10.1101/gr.6861907.

Zerr, T., G. M. Cooper, E. E. Eichler, and D. A. Nickerson. 2010. "Targeted interrogation of copy number variation using SCIMMkit." *Bioinformatics* 26 (1):120-2. doi: 10.1093/bioinformatics/btp606.
